# NEAT1_1 confers gefitinib resistance in lung adenocarcinoma through promoting AKR1C1-mediated ferroptosis defence

**DOI:** 10.1038/s41420-024-01892-w

**Published:** 2024-03-12

**Authors:** Shuman Zhen, Yunlong Jia, Yan Zhao, Jiali Wang, Boyang Zheng, Tianxu Liu, Yuqing Duan, Wei Lv, Jiaqi Wang, Fan Xu, Yueping Liu, Yi Zhang, Lihua Liu

**Affiliations:** 1 Department of Tumor Immunotherapy, Fourth Hospital of Hebei Medical University, Shijiazhuang, 050035 China; 2 China International Cooperation Laboratory of Stem Cell Research, Institute of Medical and Health Science of Hebei Medical University, Shijiazhuang, 050017 China; 3 Department of Radiotherapy, Fourth Hospital of Hebei Medical University, Shijiazhuang, 050017 China; 4 Department of Medical Oncology, Fourth Hospital of Hebei Medical University, Shijiazhuang, 050017 China; 5 Department of Oncology, Affiliated Hospital of Chengde Medical College, Chengde, 067000 China; 6 Department of Pathology, Fourth Hospital of Hebei Medical University, Shijiazhuang, 050017 China; 7 Biotherapy Center, First Affiliated Hospital of Zhengzhou University, Zhengzhou, 450052 China; 8 Cancer Research Institute of Hebei Province, Shijiazhuang, 050017 China

**Keywords:** Non-small-cell lung cancer, Cancer therapeutic resistance

## Abstract

Gefitinib is one of the most extensively utilized epidermal growth factor receptor-tyrosine kinase inhibitors (EGFR-TKIs) for treating advanced lung adenocarcinoma (LUAD) patients harboring EGFR mutation. However, the emergence of drug resistance significantly compromised the clinical efficacy of EGFR-TKIs. Gaining further insights into the molecular mechanisms underlying gefitinib resistance holds promise for developing novel strategies to overcome the resistance and improve the prognosis in LUAD patients. Here, we identified that the inhibitory efficacy of gefitinib on EGFR-mutated LUAD cells was partially dependent on the induction of ferroptosis, and ferroptosis protection resulted in gefitinib resistance. Among the ferroptosis suppressors, aldo-keto reductase family 1 member C1 (AKR1C1) exhibited significant upregulation in gefitinib-resistant strains of LUAD cells and predicted poor progression-free survival (PFS) and overall survival (OS) of LUAD patients who received first-generation EGFR-TKI treatment. Knockdown of AKR1C1 partially reversed drug resistance by re-sensitizing the LUAD cells to gefitinib-mediated ferroptosis. The decreased expression of miR-338-3p contributed to the aberrant upregulation of AKR1C1 in gefitinib-resistant LUAD cells. Furthermore, upregulated long non-coding RNA (lncRNA) nuclear paraspeckle assembly transcript 1_1 (NEAT1_1) sponged miR-338-3p to neutralize its suppression on AKR1C1. Dual-luciferase reporter assay and miRNA rescue experiment confirmed the NEAT1_1/miR-338-3p/AKR1C1 axis in EGFR-mutated LUAD cells. Gain- and loss-of-function assays demonstrated that the NEAT1_1/miR-338-3p/AKR1C1 axis promoted gefitinib resistance, proliferation, migration, and invasion in LUAD cells. This study reveals the effects of NEAT1_1/miR-338-3p/AKR1C1 axis-mediated ferroptosis defence in gefitinib resistance in LUAD. Thus, targeting NEAT1_1/miR-338-3p/AKR1C1 axis might be a novel strategy for overcoming gefitinib resistance in LUAD harboring EGFR mutation.

## Introduction

Lung cancer, one of the most prevalent and lethal malignant tumors, remains the leading cause of cancer-related mortality globally [1]. Lung adenocarcinoma (LUAD) is the most prevalent pathological subtype of lung cancer worldwide, accounting for approximately 40% of lung cancer cases worldwide [2]. LUAD possesses numerous aggressive features, such as rapidly progressing and metastasizing tendencies, leading to poor prognosis [3]. Recently, cancer therapy has entered an era of precision medicine, which benefits from discovering oncogenic driver gene alterations, among which epidermal growth factor receptor (EGFR) mutation is the most frequent targetable mutation type in LUAD [4]. The frequency of EGFR mutation in Chinese LUAD patients is significantly higher than the patients enrolled in the Cancer Genome Atlas Program (TCGA) study (50% *vs*. 14%) [5]. Due to high efficacy and satisfying safety, the first-generation EGFR-tyrosine kinase inhibitors (TKIs) (i.e., gefitinib, erlotinib, and icotinib) significantly improved the prognosis of the unresectable LUAD patients harboring classical EGFR mutation (exon 19 in-frame deletion and exon 20 L858R point mutation) [6]. However, acquired resistance is an inevitable problem in the clinical application of gefitinib. One of the most well-studied mechanisms of gefitinib resistance is the EGFR exon 20 T790M mutation, which was detected in approximately 50% of cases of first-generation TKI-resistant LUAD patients [7]. Nevertheless, the specific mechanisms of gefitinib resistance in some patients are elusive. Therefore, there is an urgent unmet need to identify novel mechanisms for further improving the prognosis of LUAD patients receiving gefitinib treatment.

The developments in broad-range gene profiling methods, such as microarray and next-generation sequencing (NGS), make it possible to overview the gene landscape of cancers [8]. Cumulative studies showed that dysregulation of programmed cell death (PCD) confers resistance to numerous therapeutic methods in cancers [9]. PCD, also known as regulated cell death (RCD), is a kind of autonomous and ordered cell death under the control of a series of genes [10]. The most common types of PCD include apoptosis, pyroptosis, necroptosis, cuproptosis, and ferroptosis, et cetera [11, 12]. Despite apoptosis and pyroptosis have been confirmed to be involved in the effect of gefitinib [13, 14], whether ferroptosis defence participated in gefitinib resistance in LUAD remains to be elucidated. We screened the differential genes between gefitinib-sensitive LUAD cells and their gefitinib-resistant strains. By enriching the genes, we found that some ferroptosis-related genes were dysregulated in gefitinib-resistant LUAD cells, among which aldo-keto reductase family 1 member C1 (AKR1C1) exhibited most significant upregulation. AKR1C1 belongs to the AKR1C family, which suppresses ferroptosis by detoxicating the reactive molecules lipid peroxides [15]. Although AKR1C1-induced ferroptosis defence in lung cancer has been confirmed by Wohlhieter et al. [16], whether it contributed to gefitinib resistance remains elusive.

In this study, we determined that AKR1C1-mediated ferroptosis defence was responsible for the gefitinib-resistance in LUAD. AKR1C1 was significantly correlated with the prognosis of the LUAD patients receiving first-generation EGFR-TKI as initial treatment. Moreover, we verified that AKR1C1 was upregulated by long non-coding RNA (lncRNA) nuclear paraspeckle assembly transcript 1_1 (NEAT1_1, also known as MENepsilon) in LUAD. Overall, we highlighted the important role of AKR1C1 in promoting gefitinib resistance and unveiled its potential upstream regulatory axis in LUAD. Taken together, our findings provide key insights into a novel NEAT1_1/AKR1C1 axis to reveal promising targets for tackling gefitinib resistance and improving the clinical outcomes of LUAD patients.

## Results

### Ferroptosis contributes to the suppressive effect of gefitinib on LUAD cells

First, we conducted a CCK-8 assay to evaluate the IC_50_ of gefitinib against gefitinib-sensitive cells (PC9 and HCC827) and matched gefitinib-resistant strains (PC9/GR and HCC827/GR). The IC_50_s of gefitinib against PC9/GR and HCC827/GR cells were significantly higher than matched parental cells (64.02 nM *vs*. 32.00 nM, 65.69 nM *vs*. 32.16 nM; supplementary Fig. S1A). Meanwhile, we detected the IC_50_ value of erlotinib and icotinib to verify whether PC9/GR and HCC827/GR were also resistant to other first-generation EGFR-TKIs. The IC_50_s of erlotinib and icotinib against PC9/GR and HCC827/GR cells were likewise significantly higher than matched parental cells (erlotinib: 203.60 nM *vs*. 106.10 nM, 155.10 nM *vs*. 84.67 nM; icotinib: 177.00 nM *vs*. 76.26 nM, 150.40 nM *vs*. 76.60 nM), indicating that the PC9/GR and HCC827/GR cells were also resistant to erlotinib and icotinib (supplementary Fig. S1B and C). EGFR T790M mutation is the most frequent mechanisms that lead to acquired resistance to first-generation EGFR-TKIs [17]. Thus, we determined the EGFR T790M mutation by conducting sequencing and found that PC9, HCC827, and their gefitinib-resistant strains did not harbor EGFR T790M mutation (supplementary Fig. S1D). Moreover, we assayed the differences in proliferation, migration, and invasion between gefitinib-resistant strains and matched parental cells. The results showed that the proliferation, migration, and invasion of PC9GR and HCC827GR cells were significantly enhanced compared to matched parental cells (supplementary Fig. S2A to C).

**Fig. 1:**
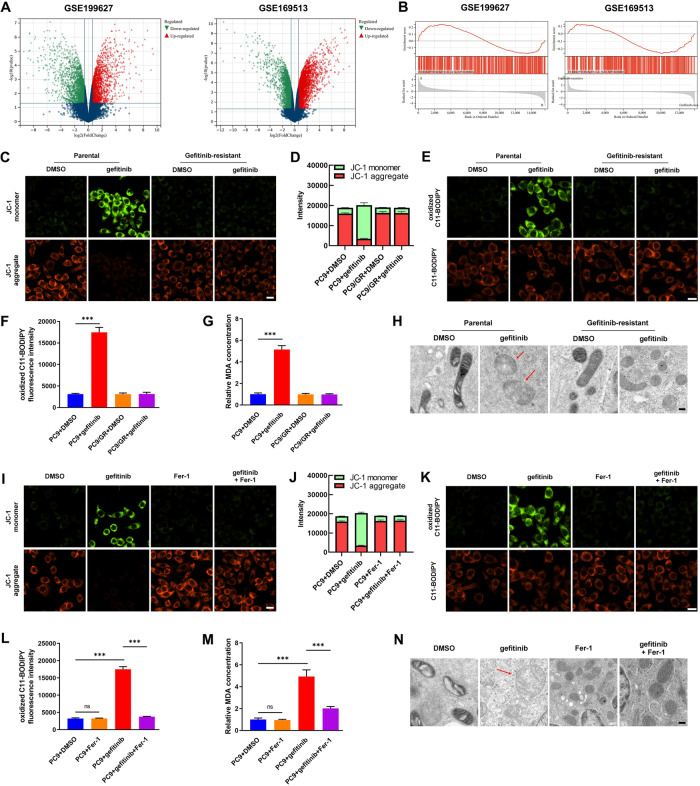
Ferroptosis protection confers gefitinib resistance in LUAD cells harboring EGFR mutation. **A** The differentially expressed protein-coding genes between gefitinib-sensitive LUAD cell line and matched gefitinib-resistant strain in GEO datasets GSE199627 and GSE169513. **B** GSEA analysis of the differentially expressed protein-coding genes between gefitinib-sensitive LUAD cell line and matched gefitinib-resistant strain in GSE199627 and GSE169513. **C–H** Effect of gefitinib on gefitinib-mediated ferroptosis-related phenomena, including JC-1 monomer/aggregate ratio, oxidized C11-BODIPY, MDA products, and mitochondria in PC9 cells. DMSO was used as control. **I–N** Effect of Fer-1 on gefitinib-mediated ferroptosis-related phenomena, including JC-1 monomer/aggregate ratio, oxidized C11-BODIPY, MDA products, and mitochondria in PC9 cells. Scale bars for JC-1 assay, 20 μm. Scale bars for TEM, 0.2 μm. ***P < 0.001.

To determine the key genes involved in gefitinib resistance in LUAD, we analysed the GEO profile GSE169513 and GSE199627, which contained the differently expressed mRNAs between gefitinib-sensitive LUAD cell lines and matched gefitinib-resistant strains. By setting the cut-off value as a fold-change of 2, we found 2545 and 2290 well-characterized differentially expressed protein-coding genes between gefitinib-resistant and gefitinib-sensitive LUAD cells in GSE169513 and GSE199627 profile, respectively (Fig. 1A and supplementary Table S3). Aiming at revealing the critical functions involved in gefitinib resistance, we then conducted a Gene Set Enrichment Analysis (GSEA) based on the protein-coding gene expression signature of the GSE169513 and GSE199627 profiles. By setting FDR < 0.10, we found 3 and 2 enriched processes in GSE199627 and GSE169513, respectively. Among these processes, only ferroptosis exhibited statistical significance in both profiles (Fig. 1B). Thus, we focused on ferroptosis to further study potential mechanisms of EGFR-TKI resistance in LUAD.

Then, we conducted the JC-1 assay, C11-BODIPY staining, MDA concentration quantification, and TEM to verify whether ferroptosis contributed to gefitinib-mediated suppression of LUAD cells. After gefitinib treatment, the specific ferroptosis phenomena, increased JC-1 monomer/aggregate ratio, increased oxidized C11-BODIPY staining, elevated MDA concentration, and swollen mitochondria were observed in PC9 and HCC827 cells instead of PC9/GR and HCC827/GR cells (Fig. 1C to H and supplementary Fig. S3A to F). Furthermore, Ferrostatin-1 (Fer-1), a well-known ferroptosis inhibitor, rescued gefitinib-induced increased JC-1 monomer/aggregate ratio, increased oxidized C11-BODIPY staining, elevated MDA concentration, and swollen mitochondria in PC9 and HCC827 cells (Fig. 1I to N and supplementary Fig. S4G to L). The above results demonstrated that the suppressive effect of gefitinib on LUAD cells was partially dependent on induction of ferroptosis.

### AKR1C1 confers resistance to gefitinib in LUAD by inhibiting ferroptosis

**Fig. 2:**
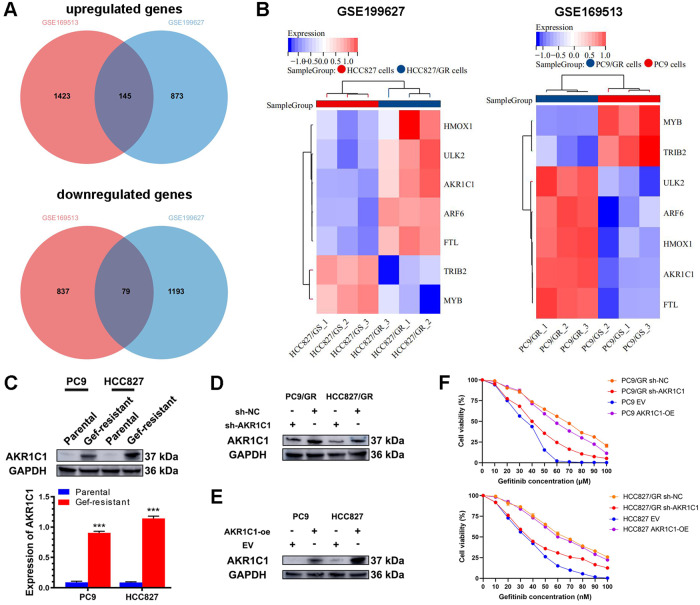
Upregulation of AKR1C1 promotes gefitinib resistance in LUAD cells. **A** Overlapping protein-coding genes of GEO datasets GSE199627 and GSE169513. **B** Heatmap showing the overlapping ferroptosis-related genes in GSE199627 and GSE169513 dataset. **C** The expression of AKR1C1 in EGFR-mutated LUAD cells and matched gefitinib-resistant strains, assayed by western blotting. **D** Effects of sh-AKR1C1 on the expression of AKR1C1 in gefitinib-resistant strains of EGFR-mutated LUAD cells. **E** Effects of AKR1C1-overexpression on the expression of AKR1C1 in gefitinib-sensitive EGFR-mutated LUAD cells. **F** Effects of sh-AKR1C1 on the sensitivity to gefitinib of PC9/GR and HCC827/GR cells, and effects of AKR1C1-overexpression on the sensitivity to gefitinib of PC9 and HCC827 cells. ***P < 0.001.

By overlapping the results from GSE169513 and GSE199627, we identified a set of 224 protein-coding genes exhibiting differential expression, among which 145 genes were upregulated and 79 genes were downregulated in gefitinib-resistant LUAD cells, compared to parental cells (Fig. 2A). Thereinto, 7 genes were ferroptosis-related genes: AKR1C1, ULK2, ARF6, HMOX1, FTL, TRIB2 and MYB (Fig. 2B). The logFCs of AKR1C1, ULK2, ARF6, HMOX1, FTL, TRIB2 and MYB in PC9/GR cells was 3.456, 1.520, 1.095, 1.183, 1.240, -3.310, -2.648, and those in HCC827/GR cells was 2.632, 1.109, 2.132, 1.907, 1.143, -5.788, -1.708, compared to matched parental cells. Notably, AKR1C1 exhibited most significant upregulation in gefitinib-resistant LUAD cells among these ferroptosis-related genes. Consequently, we selected AKR1C1 for further study. Then, we assayed the expression of AKR1C1 in gefitinib-sensitive LUAD cells and their gefitinib-resistant strains to validate the accuracy of the bioinformatic analysis. As a result, the expression of AKR1C1 was significantly higher in PC9/GR and HCC827/GR cells than in their matched parental cells (0.90 ± 0.03 vs. 0.09 ± 0.02, *P* < 0.001; 1.14 ± 0.04 vs. 0.09 ± 0.01, *P* < 0.001; Fig. 2C). For verifying whether upregulated AKR1C1 contributed to gefitinib resistance of LUAD, we knocked down AKR1C1 in gefitinib-resistant LUAD strains and overexpressed AKR1C1 in parental LUAD cells (Fig. 2D and E). Subsequently, we determined the effect of AKR1C1 on gefitinib sensitivity of LUAD cells. Knockdown of AKR1C1 decreased the IC_50_ value of gefitinib for treating PC9/GR and HCC827/GR cells (38.42 nM vs. 63.86 nM, 37.18 nM *vs*. 65.30 nM; Fig. 2F). Likewise, overexpression of AKR1C1 increased the IC_50_ value of gefitinib for treating PC9 and HCC827 cells (57.20 nM *vs*. 31.90 nM, 61.73 nM *vs*. 32.25 nM; Fig. 2F).

**Fig. 3:**
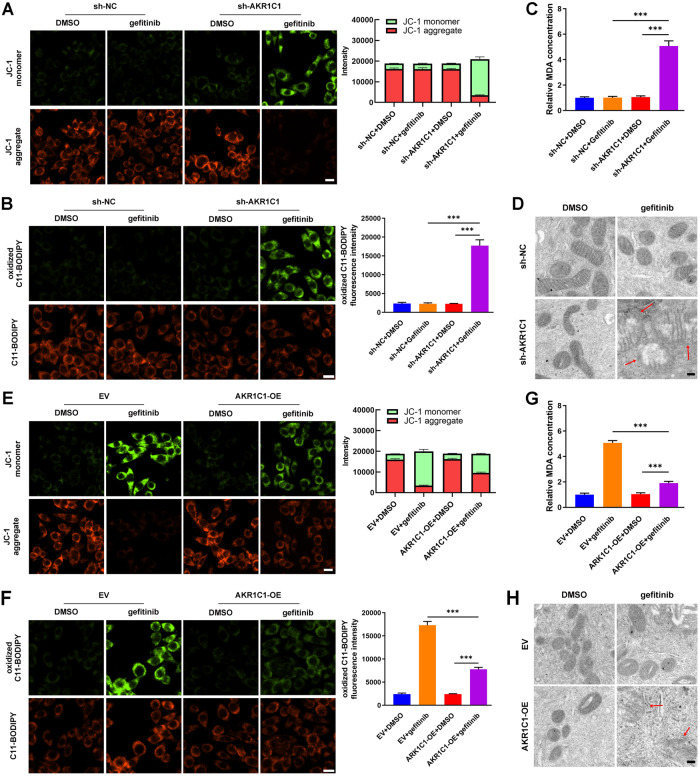
AKR1C1 contributed to ferroptosis protection in LUAD cells in vitro. **A–D** Effects of sh-AKR1C1 on JC-1 monomer/aggregate ratio, oxidized C11-BODIPY, MDA production, and mitochondria in HCC827/GR cells. **E–H** Effects of AKR1C1 overexpression on JC-1 monomer/aggregate ratio, oxidized C11-BODIPY, MDA production, and mitochondria in HCC827 cells. Scale bars for C11-BODIPY staining, 20 μm. Scale bars for TEM, 0.2 μm. ***P < 0.001.

Then, we conducted the JC-1 assay, C11-BODIPY staining, MDA concentration quantification, and TEM to determine the effect of AKR1C1 on the vulnerability of LUAD cells to gefitinib-mediated ferroptosis. Under treatment of gefitinib, the PC9/GR and HCC827/GR cells that was transfected with sh-AKR1C1 had increased JC-1 monomer/aggregate ratio, increased oxidized C11-BODIPY staining, elevated MDA concentration, and swollen mitochondria, compared with the cells transfected with sh-NC (Fig. 3A to D and supplementary Fig. S4E to H). Meanwhile, overexpression of AKR1C1 neutralized gefitinib-mediated ferroptosis in PC9 and HCC827 cells (Fig. 3E to H and supplementary Fig. S4I to L). In summary, AKR1C1 contributed to gefitinib resistance in EGFR-mutated LUAD cells by suppressing ferroptosis.

### High expression of AKR1C1 is associated with first-generation EGFR-TKI-resistance in LUAD patients

**Fig. 4:**
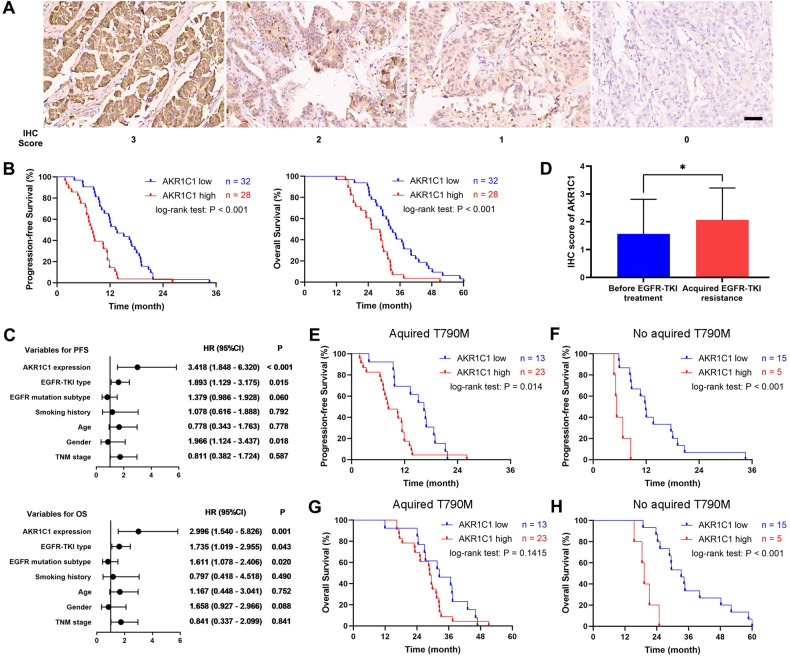
High expression of AKR1C1 predicts poor efficacy of first-generation EGFR-TKIs in advanced LUAD patients harboring EGFR-mutation. **A** Representative IHC image of AKR1C1 (brown) in LUAD tissues. Scale bars, 50 μm. **B** Kaplan-Meier analysis curves showing the difference in PFS and OS between patients with high (*n* = 28) and low *(n* = 32) expression of AKR1C1 in the tumor tissues before initial treatment. **C** Multivariate Cox proportional hazards analysis of prognostic variables for DFS and OS in LUAD patients. **D** IHC staining scores of AKR1C1 in EGFR-TKI-resistant LUAD tissues and the tissues before initial treatment. **E** Kaplan-Meier analysis curves showing the difference in PFS between patients with high (*n* = 23) and low (*n* = 13) expression of AKR1C1 in the LUAD tissues without T790M when gefitinib resistance occurred. **F** Kaplan-Meier analysis curves showing the difference in OS between patients with high (*n* = 5) and low (*n* = 15) expression of AKR1C1 in the LUAD tissues with T790M when gefitinib resistance occurred. **G** Kaplan-Meier analysis curves showing the difference in OS between patients with high (*n* = 23) and low (*n* = 13) expression of AKR1C1 in the LUAD tissues without T790M when gefitinib resistance occurred. **H** Kaplan-Meier analysis curves showing the difference in OS between patients with high (*n* = 5) and low (*n* = 15) expression of AKR1C1 in the LUAD tissues with T790M when gefitinib resistance occurred. *P < 0.05.

Given AKR1C1 conferred resistance to gefitinib in LUAD, we further evaluated the prognostic significance of the expression of AKR1C1 in the advanced-stage LUAD patients who received first-generation EGFR-TKI as first-line treatment. First, we validated the expression of AKR1C1 in the LUAD tissues before treatment and after EGFR-TKI resistance. As Fig. 4A demonstrates, the staining of AKR1C1 was mainly found in the cytoplasm of LUAD cells. Among the 60 LUAD tissues before treatment, 28 cases showed high expression of AKR1C1, while 32 cases showed low expression. Then, we evaluated the correlation between the baseline characteristics of the patients and the expression of AKR1C1 and found that the initial expression of AKR1C1 had no correlation with age, gender, TNM stage, tumor invasion, lymph node metastasis, EGFR mutation subtype or the type of EGFR-TKI they received (supplementary Table S4). Next, we conducted a Kaplan-Meier analysis to discover the prognostic significance of AKR1C1 in predicting the potential effect of EGFR-TKI. The median progression-free survival (PFS) of the patients with high and low expression of AKR1C1 was 7.95 months and 13.45 months, respectively. The PFS of the patients with high expression of AKR1C1 was significantly shorter compared to those with low expression of AKR1C1 (8.73 ± 4.86 months *vs*. 14.42 ± 6.24 months; HR = 2.371, 95% CI = 1.340–4.193; *P* < 0.001, Fig. 4B). Likewise, the OS of the patients with high expression of AKR1C1 was significantly shorter than those with low expression of AKR1C1 (26.34 ± 7.93 months *vs*. 34.73 ± 10.97 months; HR = 2.136, 95% CI = 1.204–3.787; *P* = 0.008, Fig. 4B). Furthermore, we conducted multivariate regression analysis (Cox proportional hazards model) to reveal the potential factors involved in EGFR-TKI resistance. The factors used in the Cox proportional hazards model included gender, age, smoking history, TNM stage, EGFR mutation subtype, EGFR-TKI type, and AKR1C1 expression before treatment. Among these factors, age, TNM stage, and AKR1C1 were significantly correlated with the PFS, and EGFR-TKI type, TNM stage, and AKR1C1 expression were significantly correlated with the OS (Fig. 4C).

Among the 60 patients, 36 cases (60.00%) got T790M mutation, and 4 (6.67%) lost EGFR mutation at the time of initial progression. Due to the results of this study that the upregulated AKR1C1 conferred resistance to EGFR-TKI, we next assayed the expression of AKR1C1 in the LUAD tissues resistant to EGFR-TKI. As Fig. 4D shows, the IHC scores of AKR1C1 in the EGFR-TKI-resistant LUAD tissues were significantly higher than those in the tissues before treatment (2.07 ± 1.15 *vs*. 1.57 ± 1.24, *P* = 0.0247). T790M mutation was the most common and well-known mechanism leading to resistance to first-generation EGFR-TKIs [18], and this study showed that AKR1C1 was a novel mechanism that conferred resistance to EGFR-TKI independent of T790M mutation. Therefore, we divided the patients who still harbored exon 19 del or exon 21 L858R when the disease progressed into two subgroups according to the T790M mutation status to further reveal the potential prognostic significance of AKR1C1. As expected, AKR1C1 expression exhibited significant correlation with PFS in both subgroups (Fig. 4E and F). Besides, the initial AKR1C1 expression status was correlated with the OS in the patients without acquired T790M mutation (Fig. 4G) while not with the OS in those with acquired T790M mutation (Fig. 4H). Taken above, AKR1C1 might be a T790M-independent prognostic biomarker predicting the PFS and OS of the LUAD patients who received EGFR-TKI as first-line treatment.

### LncRNA NEAT1_1 sponges miR-338-3p to upregulate AKR1C1 in LUAD

**Fig. 5:**
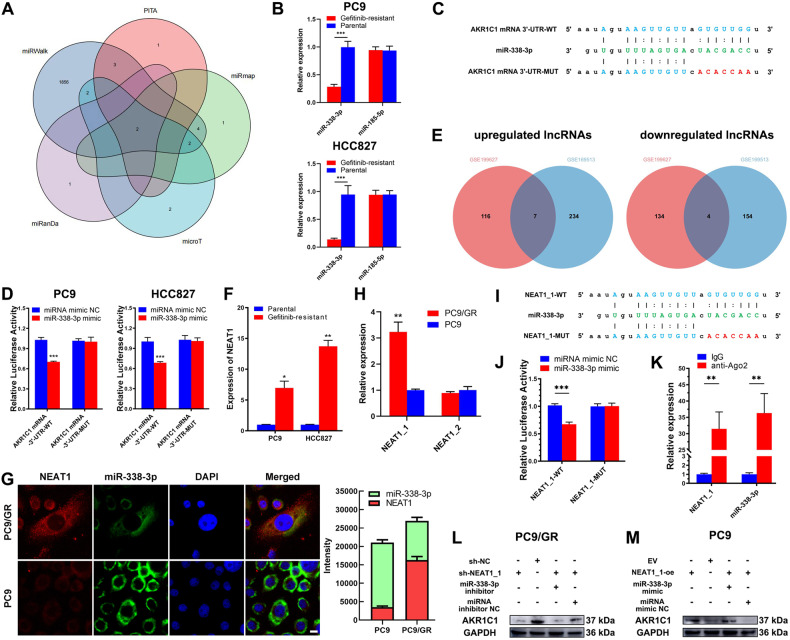
NEAT1_1 upregulates AKR1C1 by sponging miR-338-3p in LUAD cells in vitro. **A** Venn diagram showing the potential miRNAs which regulates the expression of AKRC1, predicted by 4 algorithms (PITA, miRmap, miRanDa, miRWalk, and DIANA microT). **B** The expressions of miR-338-3p and miR-185-5p in gefitinib-resistant and matched parental LUAD cells, normalized to U6. **C** Predicted sites on 3’-UTR of AKR1C1 mRNA to bind with miR-338-3p and matched established mutant sequences. **D** Luciferase reporter assay shows the luciferase activity of the LUAD cells transfected with AKR1C1 mRNA-3’-UTR-WT and AKR1C1 mRNA-3’-UTR-MUT after co-transfected with miR-338-3p mimic or miRNA mimic NC. **E** Overlapping lncRNAs of GEO datasets GSE199627 and GSE169513. **F** The expression of NEAT1 in gefitinib-resistant cell line and matched parental cells. **G** The subcellular location of NEAT1_1 (red) and miR-338-3p (green) in PC9/GR and PC9 cells, assayed by FISH. Scale bars, 10 μm**. H** The expressions of NEAT1_1 and NEAT1_2 in PC9 and PC9/GR cells, detected by qRT-PCR. **I** Predicted sites on NEAT1_1 to bind with miR-338-3p and matched established mutant sequences. **J** Luciferase reporter assay shows the luciferase activity of the PC9 cells transfected with NEAT1_1-WT and NEAT1_1-MUT after co-transfected with miR-338-3p mimic or miRNA mimic NC. **K** Enrichment level of NEAT1_1 and miR-338-3p in the Ago2 and IgG pellets of PC9 cells, respectively. **L, M** NEAT1_1/miR-338-3p/AKR1C1 axis was confirmed by performing miRNA rescue experiments in PC9/GR and PC9 cells. *P < 0.05; **P < 0.01; ***P < 0.001.

KEAP1-Nrf2 axis is the most recognized upstream regulatory mechanism of AKR1C1 in LUAD [19], but both KEAP1 and Nrf2 did not show significant difference between gefitinib-resistant LUAD cells and matched parental cells (supplementary Table S3). Thus, to reveal the potential mechanism of upregulation of AKR1C1, we focused on the miRNAs, one of the most common and well-studied epigenetic mechanisms in cancers. Then, we analysed five bioinformatic algorithms (miRmap, PITA, miRanda, miRWalk, and DIANA microT) to predict the miRNAs which potentially regulated AKR1C1. The results from bioinformatic algorithms were listed in the Supplementary Table S5. By overlapping the results from the bioinformatic algorithms, we found two miRNAs that exhibited enormous possibility to regulate AKR1C1 (Fig. 5A). Based on the above results, we next assayed the expression of these two miRNAs in the gefitinib-resistant LUAD cells and matched parental cells. The expressions of miR-338-3p were significantly lower in PC9/GR and HCC827/GR cells compared to matched parental cells (Fig. 5B). To determine whether miR-338-3p targeted the 3’-UTR of AKR1C1 mRNA in LUAD cells, we constructed luciferase reporters carrying AKR1C1 mRNA with mutation in the predicted binding site (i.e., AKR1C1 mRNA-3’-UTR-MUT) for binding miR-338-3p (Fig. 5C). Then, the reporters carrying AKR1C1 mRNA-3’-UTR-MUT or AKR1C1 mRNA-3’-UTR-wild type (WT) was introduced into PC9 and HCC827 cells, followed by cotransfection with miR-338-3p mimics. Co-transfection of miR-338-3p mimic reduced the luciferase activity in the cells that were transfected with the reporter carrying AKR1C1 mRNA-3’-UTR-WT. In contrast, it did not reduce that in the cells transfected with the reporter carrying AKR1C1 mRNA-3’-UTR-MUT (Fig. 5D).

The suppressive effects of miRNAs on target mRNAs could be neutralized by lncRNAs [20]. Thus, some upregulated lncRNAs might contribute to the loss of function of miR-338-3p in PC9/GR and HCC827/GR cells. Therefore, we used the bioinformatics algorithm miRanDa (http://www.microrna.org/) to screen the lncRNAs that held the potential to bind with miR-338-3p and consequently found 20 well-characterized lncRNAs (supplementary Table S6). Then, we re-analysed the GEO profiles GSE199627 and GSE169513 to screen the differentially expressed lncRNAs between gefitinib-sensitive LUAD cells and their matched gefitinib-resistant strains. By setting fold-change as 2, we found 241 and 123 upregulated well-characterized lncRNAs in PC9/GR and HCC827/GR cells, compared to their matched parental cells, respectively (Fig. 5E and supplementary Table S7). Furthermore, we found 7 overlapped lncRNAs that were upregulated in both PC9/GR and HCC827/GR cells (Fig. 5E), among which only NEAT1 could be found in the results from miRanDa. Subsequently, we analysed the expression of NEAT1 by performing qRT-PCR to determine whether its expression had significance between gefitinib-resistant LUAD cells and matched parental cells (Fig. 5F). Thus, we selected NEAT1 for subsequent study.

We then conducted the FISH to determine the subcellular location of NEAT1 and miR-338-3p in PC9/GR and HCC827/GR cells. We found that the predominant subcellular location of NEAT1 and miR-338-3p was cytoplasm in LUAD cells (Fig. 5G and supplementary Fig. S5A). Besides, the NEAT1 expression was significantly higher and miR-338-3p expression was significantly lower in PC9/GR and HCC827/GR cells, compared to those in matched parental cells (Fig. 5G and supplementary Fig. S5A). There are two transcriptional variants NEAT1, i.e., NEAT1_1 (NR_028272.1, 3.7 kb) and NEAT1_2 (NR_131012.1, 22.7 kb), and NEAT1_1 completely overlapped with the 5′ end of NEAT1_2 but had a shorter 3′ end [21]. The site for binding miR-338-3p was found in both NEAT1_1 and NEAT1_2. Therefore, to elucidate which variant was upregulated in PC9/GR and HCC827/GR cells, we referred to Wen’s study to design the specific primers for detecting NEAT1_1 and NEAT1_2 [22]. As Fig. 5H and supplementary Fig. S5B show, NEAT1_1 exhibited significant upregulation in PC9/GR and HCC827/GR cells (3.23 ± 0.38 vs. 1.00 ± 0.04, *P* = 0.009; 3.45 ± 0.58 vs. 1.03 ± 0.13, *P* = 0.015) instead of NEAT1_2 (0.89 ± 0.06 vs. 1.01 ± 0.13, *P* = 0.233; 1.00 ± 0.18 vs. 1.02 ± 0.22, *P* = 0.916), compared to PC9 and HCC728 cells, respectively. In summary, NEAT1_1 and miR-338-3p were co-located in the cytoplasm of PC9/GR and HCC827/GR cells.

To verify whether NEAT1_1 sponged miR-338-3p in LUAD cells, we constructed luciferase reporters carrying NEAT1_1 with mutation in the predicted binding site (i.e., NEAT1_1-MUT) for binding miR-338-3p (Fig. 5I). Besides, the luciferase reporters carrying wild-type of NEAT1_1 (i.e., NEAT1_1-WT) was constructed as control. Then, the luciferase reporters carrying NEAT1_1-WT or NEAT1_1-MUT were respectively transfected into the PC9 and HCC827 cells, followed by co-transfection of miR-338-3p mimics. The results showed that the luciferase activities of the PC9 and HCC827 cells, which were transfected with NEAT1_1-WT were reduced by miR-338-3p mimics (0.67 ± 0.04 vs. 1.02 ± 0.03, *P* < 0.001; 3.45 ± 0.58 vs. 1.03 ± 0.13, *P* = 0.015), while miR-338-3p mimics did not reduce the luciferase activities of the PC9 and HCC827 cells which were transfected with NEAT1_1-MUT (1.00 ± 0.05 vs. 1.00 ± 0.05, *P* = 0.927; 3.45 ± 0.58 vs. 1.03 ± 0.13, *P* = 0.015) (Fig. 5J and supplementary Fig. S5C). Then, we performed Ago2-RIP and qRT-PCR to verify whether NEAT1_1 and miR-338-3p occupied the same Ago2 protein to form an RNA-induced silencing complex (RISC). Consequently, NEAT1_1 and miR-338-3p were significantly more enriched in Ago2 pellets than in IgG pellets in PC9 and HCC827 cells (Fig. 5K and supplementary Fig. S5D). The above results demonstrated that NEAT1_1 directly bound with miR-338-3p to form a RISC in LUAD.

Next, we aimed to determine whether NEAT1_1 upregulated AKR1C1 by sponging miR-338-3p in LUAD cells. Thus, we established NEAT1_1-knockdown PC9/GR and HCC827/GR cells with shRNA and then cotransfected miR-338-3p into these cells. Knockdown of NEAT1_1 significantly decreased the expression of AKR1C1 in PC9/GR and HCC827/GR cells, and miR-338-3p inhibitor rescued this downregulation (Fig. 5L and supplementary Fig. S5E). Besides, overexpression of NEAT1_1 significantly increased the expression of AKR1C1 in PC9 and HCC827 cells, and miR-338-3p mimic partially rescued this upregulation (Fig. 5M and supplementary Fig. S5F). In conclusion, the above results demonstrated that NEAT1_1 sponged miR-338-3p to upregulate AKR1C1 in LUAD cells.

### NEAT1_1/miR-338-3p/AKR1C1 axis promotes gefitinib resistance and progression in LUAD in vitro and in vivo

**Fig. 6:**
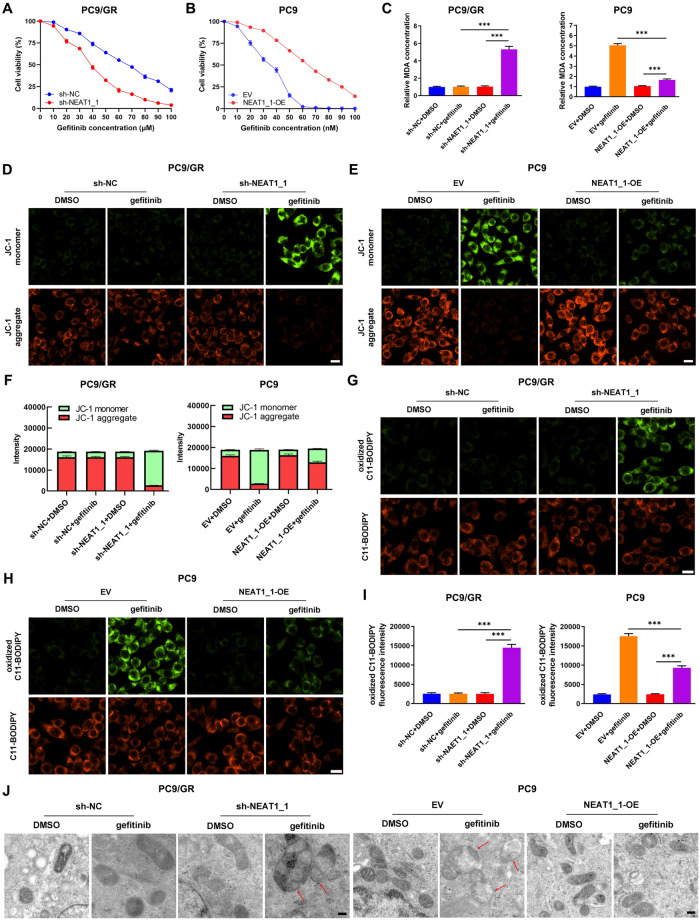
NEAT1_1 induces ferroptosis protection and gefitinib resistance in LUAD in vitro. **A** Effects of sh-NEAT1_1 on the sensitivity to gefitinib of PC9/GR cells. **B** Effects of NEAT1_1-overexpression on the sensitivity to gefitinib of PC9 cells. **C** Effects of NEAT1_1 on MDA production in PC9/GR and PC9 cells. **D, E** Representative fluorescence of JC-1 monomer and aggregate in PC9/GR and PC9 cells. Scale bars, 20 μm. **F** Effect of NEAT1_1 on the JC-1 monomer/aggregate ratio in PC9/GR and PC9 cells. **G, H** Representative fluorescence of C11-BODIPY in PC9/GR and PC9 cells. Scale bars, 20 μm. **I** Effect of NEAT1_1 on oxidized C11-BODIPY in PC9/GR and PC9 cells. **J** Representative TEM image of PC9/GR and PC9 cells treated with DSMO or gefitinib. Scale bars, 0.2 μm. ***P < 0.001.

Given that NEAT1_1 upregulated AKR1C1 by sponging miR-338-3p in LUAD cells, its underlying effect on gefitinib resistance was further examined. First, we performed a CCK-8 assay to study the effect of NEAT1_1 on the gefitinib sensitivity of LUAD cells. Knockdown of NEAT1_1 significantly decreased the IC_50_ value of gefitinib for treating PC9/GR and HCC827/GR cells (37.26 nM *vs*. 63.67 nM, 34.53 nM *vs*. 65.04 nM; Fig. 6A and supplementary Fig. S6A), and overexpression of NEAT1_1 increased the IC_50_ value of gefitinib for treating PC9 and HCC827 cells (32.01 nM *vs*. 62.46 nM, 32.49 nM *vs*. 64.71 nM; Fig. 6B and supplementary Fig. S6B). Besides, sh-NEAT1_1 partially abolished PC9/GR and HCC827/GR cells’ resistance to gefitinib-mediated ferroptosis, and overexpression of NEAT1_1 neutralized gefitinib-mediated ferroptosis in PC9 and HCC827 cells (Fig. 6C to J and supplementary Fig. S6C to J). In summary, NEAT1_1 conferred resistance to gefitinib by suppressing ferroptosis in LUAD.

**Fig. 7:**
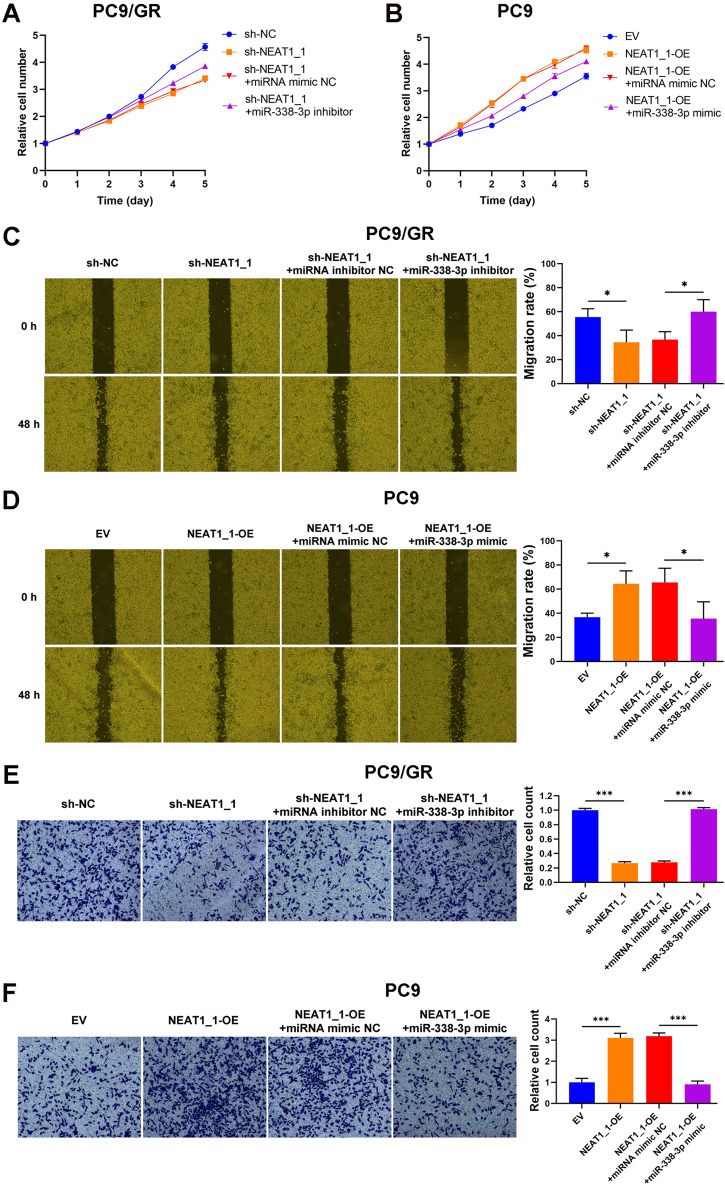
NEAT1_1/miR-338-3p/AKR1C1 axis promotes malignant behaviors of LUAD cells in vitro. **A, B** Effects of NEAT1_1/miR-338-3p/AKR1C1 axis on the proliferation ability of PC9/GR and PC9 cells. **C, D** Effects of NEAT1_1/miR-338-3p/AKR1C1 axis on the migration ability of PC9/GR and PC9 cells. **E, F** Effects of NEAT1_1/miR-338-3p/AKR1C1 axis on the migration ability of PC9/GR and PC9 cells. *P < 0.05; ***P < 0.001.

Since AKR1C1 was reported to promote proliferation, migration, and invasion of NSCLC cells [23, 24], we then studied the effect of NEAT1_1/miR-338-3p/AKR1C1 axis on these malignant behaviors of PC9 and HCC827 cells. Knockdown of NEAT1_1 significantly suppressed the proliferation, migration and invasion in PC9/GR and HCC827/GR cells, and miR-338-3p inhibitor neutralized these suppressive effects (Fig. 7A to F and supplementary Fig. S7A to F). The above results showed that the NEAT1_1/miR-338-3p/AKR1C1 axis promoted proliferation, migration, and invasion in LUAD.

**Fig. 8:**
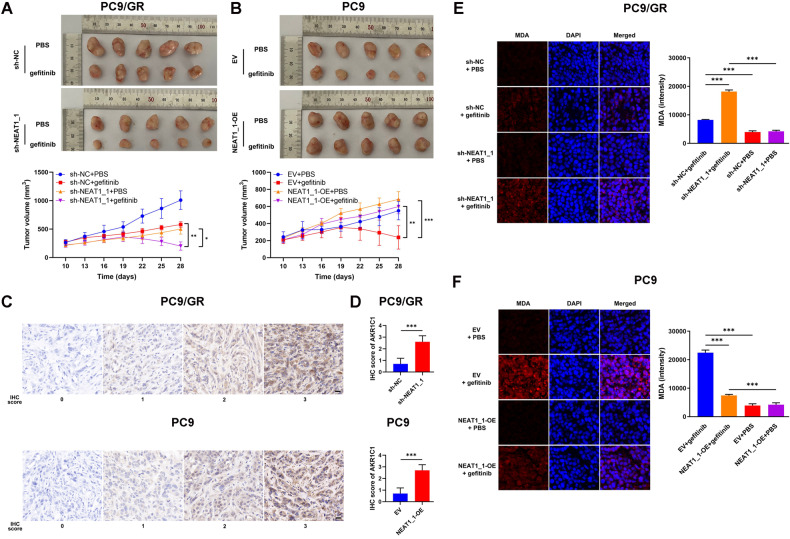
The NEAT1_1/miR-338-3p/AKR1C1 axis contributes to gefitinib resistance of LUAD in vivo. **A** PC9/GR cell-derived xenograft tumors of sacrificed mice with gefitinib or PBS treatment at the end of the experiment and their growth curves. **B** PC9 cell-derived xenograft tumors of sacrificed mice with gefitinib or PBS treatment at the end of the experiment and their growth curves. **C** Representative IHC images of AKR1C1 (brown) in xenograft tumor tissues derived from PC9/GR or PC9 cells. Scale bars, 50 μm. **D** IHC staining scores of AKR1C1 in xenograft tumor tissues derived from PC9/GR or PC9 cells. **E, F** Representative IF images of MDA (red) in xenograft tumor tissues derived from PC9/GR or PC9 cells. **G** Schematic illustration depicting a proposed model of the molecular mechanism of NEAT1_1/miR-338-3p/AKR1C1 axis in initiating gefitinib resistance in LUAD. *P < 0.05; **P < 0.01; ***P < 0.001.

To evaluate the role of NEAT1_1 in gefitinib resistance and progression in LUAD in vivo, we established a xenograft tumor model with PC9 and PC9/GR cells. Although gefitinib suppressed the tumor growth of both groups of PC9/GR cells, only the PC9/GR cells of sh-NEAT1_1 group exhibited tumor regression, indicating that knockdown of NEAT1_1 partially reversed gefitinib resistance in vivo (Fig. 8A). Besides, under-treatment of gefitinib, the PC9 cells of EV group regressed from the 22^nd^ day, but the tumors derived from the PC9 cells of NEAT1_1-OE group kept growing (Fig. 8B). The xenograft tumor volumes of PC9 cells of NEAT1_1-OE group were significantly larger than those of PC9 cells of EV group (Fig. 8B). Then, we assayed the expression of AKR1C1 in xenograft tumors to confirm the existence of NEAT1_1/miR-338-3p/AKR1C1 axis. AKR1C1 staining was predominantly found in the cytoplasm of cancer cells in xenograft tumors (Fig. 8C). The IHC scores of AKR1C1 in tumors derived from PC9/GR cells of sh-NEAT1_1 group were significantly lower than those of sh-NC group (0.70 ± 0.48 vs. 2.60 ± 0.52, *P* < 0.001; Fig. 8D). Likewise, the IHC scores of AKR1C1 in xenograft tumors derived from PC9 cells of NEAT1_1-OE group were significantly higher than those of EV group (0.70 ± 0.48 vs. 2.70 ± 0.48, *P* < 0.001; Fig. 8D). Furthermore, we conducted IF to detect the MDA for determining the effect of NEAT1_1/miR-338-3p/AKR1C1 axis on gefitinib-mediated ferroptosis in vivo. As Fig. 8E shows, MDA could be observed in the cytoplasm of cancer cells of the xenograft tumors. Knockdown of NEAT1_1 further improved the gefitinib-induced increase of intensity of MDA in PC9/GR cells, and overexpression of NEAT1_1 partially neutralized the gefitinib-induced increase of intensity of MDA in PC9 cells (Fig. 8F). The above results indicated that NEAT1_1/miR-338-3p/AKR1C1 axis resistance and progression of LUAD cells in vivo.

## Discussion

Gefitinib, the first approved EGFR-TKI, is still widely used as first-line treatment agent in advanced NSCLC patients harboring EGFR mutations, especially exon 19 deletion and exon 20 L858R point mutation [25]. Gefitinib exhibited outstanding efficacy in improving the PFS in the patients with EGFR-mutated advanced LUAD, but resistance has become an unavoidable obstacle to sustained benefit [26]. One of the most frequent mechanisms of gefitinib resistance is secondary EGFR mutation, such as the exon 20 T790M mutation, which is targeted by the third-generation EGFR-TKI osimertinib [27]. Besides the secondary EGFR mutations, amplification, loss, and mutation of some other cancer-related genes also contribute to gefitinib resistance, such as MET, HER2, BRAF, and PIK3CA [28]. However, the present understanding of the mechanisms of gefitinib resistance still cannot satisfy the clinical demand. Therefore, by clarifying the mechanisms of EGFR-TKI resistance that are independent of the T790M, we can identify novel potential targets for overcoming resistance and further improve the clinical outcome of the patients with advanced LUAD.

In this study, we screened the DEGs between gefitinib-resistant strains and their matched parental LUAD cells and then utilized GSEA analysis to explore the potential functions of these DEGs. We found that ferroptosis was an overlapped biological function of the DEGs in both two PC9/GR and HCC827/GR cells. Ferroptosis is a novel type of PCD that is driven by iron-dependent lipid reactive oxygen species (ROS) accumulation-mediated lipid peroxidation [29]. During ferroptosis, the mitochondria are damaged by lipid peroxidation, and thereby, they are swollen, and their membrane potential is decreased [30]. Besides, ferroptosis is characterized by increased MDA, a featured products of lipid peroxidation [31]. Although gefitinib has been confirmed to induce ROS and increase the oxidative stress [32–34], whether its effects in EGFR-mutated LUAD are dependent on ferroptosis remain elusive. Thus, we assayed the mitochondria and MDA in EGFR-mutated cells to evaluate the effect of gefitinib on ferroptosis in EGFR-mutated LUAD cells. Consequently, gefitinib resulted in increased JC-1 monomer/aggregate ratio, swollen mitochondria and elevated MDA concentration in PC9 and HCC827 cells, which could be neutralized by ferroptosis inhibitor Fer-1. In contrast, gefitinib did not induce increased JC-1 monomer/aggregate ratio, swollen mitochondria and elevated MDA concentration in PC9/GR and HCC827/GR cells. Taken together, our results demonstrated that gefitinib might induce ferroptosis in EGFR-mutated LUAD cells, and ferroptosis defence contributed to gefitinib resistance.

The most well-studied ferroptosis defence systems in cancers include the GPX4-GSH, FSP1-CoQH2, GCH1-BH4 and DHODH-CoQH2 system [35]. The AKR1C family (AKR1C1/2/3) is another ferroptosis defence system which is first defined by Gagliardi et al. [15] AKR1C family catalyzes the conversion of aldehydes and ketones to their corresponding alcohols and thereby detoxicates the reactive molecules lipid peroxides which are essential for mediating ferroptosis [15]. In present study, we confirmed that the expression of AKR1C1 was upregulated in gefitinib-resistant LUAD cells, and knockdown of AKR1C1 partially overcame the resistance by increasing the sensitivity to gefitinib-mediated ferroptosis. In addition, we found that the high expression of AKR1C1 might be a potential biomarker for predicting the therapeutic efficacy of first-generation EGFR-TKIs in advanced stage LUAD patients.

Next, we explored the mechanisms of the upregulation of AKR1C1 in gefitinib-resistant LUAD cells. Competing endogenous RNA (ceRNA) regulatory network is a well-studied and frequent mechanism conferring alteration of gene expression in cancers [36, 37]. In brief, some cytoplasmic lncRNAs and circRNAs are capable to sponge target miRNAs and neutralize their suppressive effects on corresponding mRNAs [37, 38]. Cumulative studies have revealed some ceRNA networks participate in the regulation of gefitinib resistance in LUAD, such as PCAT6/miR-326/IFNAR2, SNHG15/miR-451/MDR1, and UCA1/miR-143/FOSL2 axis [39, 40]. Herein, we identified miR-338-3p was a downregulated miRNA that conferred the upregulated expression of AKR1C1 in gefitinib-resistant LUAD cells. Moreover, we found NEAT1 might act as a ceRNA to sponge miR-338-3p in LUAD. NEAT1 is a highly conserved single exon, intergenic lncRNA frequently upregulated in numerous cancers, including nasopharyngeal carcinoma, esophageal squamous cell carcinoma, and NSCLC, et cetera [41]. NEAT1, also known as linc00084, has two distinct transcripts according to the 3’-end processing pattern: NEAT1_1 and NEAT1_2 [42]. Although the tumor-promoting roles of NEAT1_2 are well-documented, the expression and functions of NEAT1_1 in cancers still need more adequate study [43]. NEAT1_1 was reported to be an oncogene in neuroblastoma, breast cancer and renal cell carcinoma [44–46]. As for NSCLC, although the imbalance of NEAT1_1/NEAT1_2 ratio contributed to invasion and metastasis, the precise role of NEAT1_1 was elusive [47]. Herein, we found that NEAT1_1 exhibited upregulation in gefitinib-resistant strains of PC9 and HCC827 cells. Noguchi et al. reported that gefitinib could stimulate the formation of NACHT, LRR and PYD-containing protein 3 (NLRP3) inflammasome [48]. Besides, Zhang et al. observed that NEAT1_1 was upregulated and translocated from nucleus to cytoplasm during the formation and activation of NLRP3 inflammasome [49]. Thus, the upregulation of NEAT1_1 in the PC9/GR and HCC827/GR cells might be due to the gefitinib-induced formation of NLRP3 inflammasome. Moreover, in this study, we confirmed that NEAT1_1 acted as a ceRNA to upregulate AKR1C1 by sponging miR-338-3p, and thereby promoted ferroptosis defence to confer gefitinib resistance in LUAD cells.

**Fig. 9:**
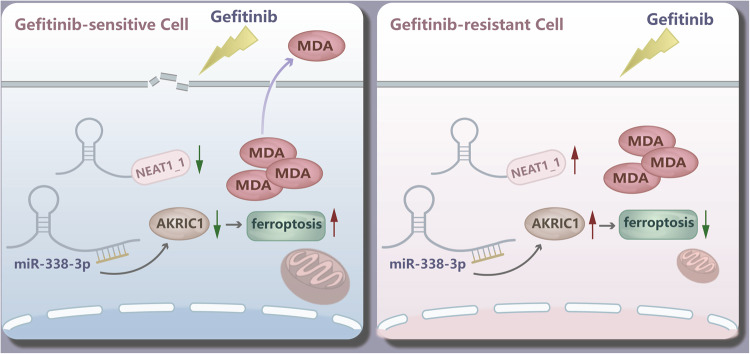
Proposed model of NEAT1_1 conferring gefitinib resistance in LUAD by upregulating AKR1C1. NEAT1_1 sponges miR-338a-3p and upregulates AKR1C1 in LUAD cells harboring classical EGFR mutation. NEAT1_1/miR-338a-3p/AKR1C1 axis drives a ferroptosis protection and thereby promotes gefitinib resistance and malignant behaviors.

In conclusion, our findings showed that AKR1C1 conferred-gefitinib resistance in LUAD cells by suppressing ferroptosis. AKR1C1 might have clinical potential to be a biomarker for predicting gefitinib resistance in LUAD patients with classical EGFR mutation. Moreover, NEAT1_1 contributed to upregulation of AKR1C1 by sponging miR-338-3p in LUAD cells (Fig. 9). Thus, targeting NEAT1_1/miR-338-3p/AKR1C1 axis might be a novel strategy for overcoming gefitinib resistance in LUAD.

## Materials and methods

### Cell culture

Human LUAD cell lines PC9 and HCC827, which harbored EGFR exon 19 in-frame deletion mutation, were purchased from Procell Life Science and Technology (Wuhan, China). The gefitinib-resistant strains of PC9 and HCC827, termed PC9/GR and HCC827/GR, were purchased from CellCook Biotechnology (Guangzhou, China). All cells were maintained in RPMI-1640 medium (Biological Industries, Beit-Haemek, Israel) containing 5% fetal bovine serum (FBS) (VivaCell, Denzlingen, Germany), 100 U/ml penicillin and 100 µg/ml phytomycin (Servicebio, Wuhan, China) in the incubator with 5% CO_2_ at 37 °C. For all in vitro studies, PC9/GR and HCC827/GR cells were cultured in gefitinib-free medium for seven days to eliminate gefitinib before use. To ensure the cells were not contaminated with mycoplasma, the mycoplasma contamination was detected using the Mycoplasma test kit (C0297, Beyotime, Shanghai, China) every 2 months. The cells were authenticated using STR profiling before use.

### Bioinformatic analysis

Microarray data profile GSE169513 and high throughput sequencing profile GSE199627 were downloaded from the Gene Expression Omnibus (GEO) database (http://www.ncbi.nlm.nih.gov/geo/) to study the genes involved in gefitinib resistance in PC9 and HCC827 cells, respectively. We screened the differentially expressed genes (DEGs) using the limma package in R.

### Patients and specimens

60 cases of patients initially diagnosed as advanced stage LUAD between Jan. 2014 and Dec. 2020 in Fourth Hospital of Hebei Medical University were retrospectively studied. All the patients met the following conditions: 1. initially diagnosed as unresectable stage; 2. did not harbor T790M mutation when initially diagnosed and received first-generation EGFR-TKI (gefitinib, erlotinib, and icotinib) as first-line treatment; 3. underwent a secondary biopsy when tumor progressed and the residual paraffin-embedded tissue was enough for sectioning. None of these LUAD patients received antitumor treatment, including radiotherapy, chemotherapy, or immunotherapy, et cetera. All patients harbored EGFR exon 19 in-frame deletion or exon 21 L858R point mutation and no T790M mutation before EGFR-TKI treatment. The clinical stage of patients was determined according to Union for International Cancer Control (UICC) Classification of 2017 (eighth edition). Complete follow-up was updated until death or May 2023. All written informed consents were obtained.

### Cell viability assay

Cell viability was measured using Cell Counting Kit-8 (CCK-8) (Servicebio). In brief, cells were plated in 96-well plates at the density of 1 × 10^4^ cells per well. When cells adhered, different concentrations of gefitinib, erlotinib, or icotinib (Energy Chemical, Shanghai, China) were added into the wells. The same volume of DMSO was added into the control well. After 24 h incubation, 10 μl of CCK-8 reagent was added to each well, and the plates were incubated for an additional 30 min. The absorbance was read at 450 nm. The IC_50_ value was calculated based on the non-linear regression fit method by GraphPad Prism 9.5.1 software (GraphPad Software, San Diego, CA, USA).

### RNA isolation and quantitative RT-PCR (qRT-PCR) assay

In brief, total RNA was extracted using TRIzol reagent (Thermo Fisher Scientific, Waltham, MA, USA). cDNA was synthesized using the RevertAid First Strand cDNA Synthesis Kit (Thermo Fisher Scientific). qRT-PCR was conducted with Hieff qPCR SYBR Green Master Mix (Yeasen, Shanghai, China) on ABI Prism 7900-HT Sequence Detection System (Applied Biosystems, Carlsbad, CA, USA). 2^-△△CT^ method was conducted to calculate the relative gene expression levels [50]. For calculating the relative expression of lncRNAs and microRNAs (miRNAs), GAPDH and U6 small nuclear RNA were used as internal controls, respectively. The primers were synthesized by Sangon Biotech (Shanghai, China), and the sequences were listed in Supplementary Table S1.

### miRNA inhibitor and mimic transfection

The miR-338-3p mimic, miR-338-3p inhibitor, miRNA mimic NC, and miRNA inhibitor NC were purchased from RiboBio (Guangzhou, China). Transfection was carried out using according to the manufacturer’s instructions.

### JC-1 assay

The mitochondrial membrane potential was evaluated using the JC-1 assay kit (Servicebio). The LUAD cells at the logarithmic growth stage were obtained and resuspended using RMPI-1640 medium without FBS. 1×10^5^ cells were plated in the 6-well plate per well. When the cells adhered, gefitinib or DMSO was added into the wells and discarded after 24 h. Then, 1 ml RMPI-1640 medium and 1 ml JC-1 dye working solution were added into each well. After incubation at 37 °C for 20 min, the supernatant was discarded, and the cells were washed using JC-1 dye buffer. The cells were observed with laser confocal microscopy. The experiment was repeated three times.

### C11-BODIPY 581/591 staining

The lipid peroxidation levels were measured using BODIPY 581/591 C11 fluorescent probe (ABclonal, Wuhan, China). The LUAD cells were incubated with 10 μM BODIPY 581/591 C11 dye for 30 min at 37 °C. Then, cells were washed with PBS and observed under confocal microscopy.

### Western blotting

Cells were lysed in lysis buffer, and protein concentrations were measured with the BCA protein assay kit (Report Biotechnology, Shijiazhuang, China). Proteins were separated by 10% SDS-PAGE and transferred electrophoretically onto polyvinylidene difluoride membranes (Millipore, Billerica, MA, USA). The membranes were incubated in PBS containing 5% bovine serum albumin for 2 h at room temperature, followed by overnight incubation at 4 °C with different dilutions of the primary antibodies, including antibodies to AKR1C1 (GTX105620, GeneTex, Alton Pkwy Irvine, CA, USA), MET (#3127, Cell Signaling Technology, Danvers, MA, USA), GAPDH (GB15004, Servicebio). The membranes were developed with the Odyssey infrared imaging system according to the manufacturer’s instructions. The protein levels in each sample were normalized relative to those of GAPDH. Each experiment contained triplicate wells of each sample, and all experiments were repeated three times.

### Transmission electron microscope (TEM)

After treatment, the LUAD cells were fixed with phosphate-buffered glutaraldehyde (2.5%) (Servicebio). Then, the cells were post-fixed in osmium tetroxide (1%) for 2 h away from light. The fixed cells were then dehydrated in acetone solutions at increasing concentrations and embedded in an epoxy resin. Then, the sections (60 - 80 nm) were stained with uranyl acetate and lead citrate. Ultrastructural images were captured with a transmission electron microscope (Hitachi HT7800, Tokyo, Japan).

### Immunohistochemistry (IHC)

IHC staining was performed and analysed according to standard protocols as described in our previous study [51]. The rabbit polyclonal antibody against AKR1C1 (GTX105620, GeneTex) was used for detection. The staining was evaluated using IHC Profiler of Image J (National Institutes of Health, Bethesda, MD, USA). The staining scores of 0 and 1 were regarded as low expression, while 2 and 3 were considered high expression.

### Fluorescence in situ hybridization (FISH) assay

The double FISH assay was performed as previously described [52]. FISH probes were designed and synthesized by Servicebio. Dig-labeled probes specific to NEAT1 and probes biotin-labeled probes specific to miR-338-3p were used in hybridization. Probe sequence: NEAT1: 5’-CAAGTTGAAGATTAGCCCTC-3’, miR-338-3p: 5’-CAACAAAATCACTGATGCTGGA-3’. The samples were counterstained with 6-diamidino-2-phenylindole (DAPI) and observed by confocal microscopy.

### Quantification of malondialdehyde (MDA) level

The lipid peroxidation was assessed using an MDA assay kit (Beyotime,) according to the manufacturer’s instructions. The cells were harvested and lysed to extract the total protein. After quantified using a BCA protein assay kit, 0.1 ml protein was mixed with the 0.2 ml MDA working buffer and heated at 100 °C for 15 min. After cooling to room temperature, 0.2 ml supernatant was added into the 96-well plate, and the absorbance was measured at 532 nm.

### Transfection

The miR-338-3p mimic, miR-338-3p inhibitor, miRNA mimic NC, and miRNA inhibitor NC were purchased from Thermo Fisher Scientific. Short hairpin RNAs (shRNAs) for knocking down AKR1C1 or NEAT1_1 were synthesized and packaged by RiboBio. The vector used for packaging shRNAs was pLVX-shRNA2-Puro. The full length of AKR1C1 or NEAT1_1 was packaged in vector pCDH-CMV-MCS-EF1-Puro, termed as AKR1C1-OE or NEAT1_1-OE, for overexpressing the corresponding gene in LUAD cells. Cells were transfected using HighGene transfection reagent (ABclonal), according to the manufacturer’s instructions. Stable cells were selected by 4 mg/ml puromycin after lentivirus infection. The target sequences of shRNAs are provided in Supplementary Table S2.

### Dual-luciferase reporter assay

The wild-type or mutant NEAT1_1 or 3’-untranslated region (UTR) of AKR1C1 containing the predicted binding sites of miR-338-3p were designed and synthesized by GenePharma (Shanghai, China). The luciferase activity of firefly luciferase and Renilla luciferase was measured using a dual-luciferase reporter assay system (GenePharma) according to the manufacturer’s instructions. The firefly luciferase was used as internal control to normalize the Renilla luciferase. All transfection assays were carried out in triplicate.

### Wound healing assay

For detecting the migration ability, LUAD cells were seeded and cultured with serum-free RMPI-1640 medium in 6-well plates, the monolayer cells were scraped linearly to introduce an artificial wound that was captured at 0 h and 48 h.

### Transwell invasion assay

The inserts of transwell chambers (Corning, New York, NY, USA) were coated with 50 μl of 1 mg/ml Matrigel matrix (Solarbio), according to the manufacturer’s instructions. 5 × 10^4^ LUAD cells in 200 μl of FBS-free medium were plated in the upper chamber, and 600 μl of medium with 10% FBS were added to the lower chamber. After incubation for 24 h at 37 °C with 5% CO_2_, cells that did not penetrate the membrane were removed using a cotton swab, and the invading cells were fixed with 0.1% crystal violet (Report Biotechnology).

### RNA immunoprecipitation (RIP) assay

RIP was conducted using a Magna RIP RNA-Binding Protein Immunoprecipitation kit (Millipore, Billerica, MA, USA), according to the manufacturer’s instructions [52].

### Nude mouse xenograft tumor model

The animal experiment was approved by the Animal Research Committee of the Fourth Hospital of Hebei Medical University. 40 female BALB/c nude mice (4-week old) were purchased from HFK Bioscience (Beijing, China) and maintained under pathogen-free conditions. The 40 nude mice were randomly divided into 8 groups, among which 4 groups were injected with PC9/GR cells, and the other 4 groups were injected with PC9 cells. The PC9/GR cells used for injection were previously treated with sh-NEAT1_1 or sh-NC, and the PC9 cells were previously treated with a NEAT1_1-OE vector or EV. LUAD cells were injected into the dorsal flank of the mice to form xenograft tumors. 1×10^7^ PC9 or PC9/GR cells were subcutaneously injected into the flank of mice to establish a tumor-bearing mouse model. 10 days after injection, the mice were fed with gefitinib (30 mg/kg) or PBS per day. The growth of tumors was monitored at a 3-day interval. The length and width of the tumors were measured every 4 days and the tumor volume was calculated by using the formula: 1/2×L×W^2^. The xenograft tumors were harvested after 4 weeks.

### Immunofluorescence (IF) assay

The xenograft tumors of the mouse model were fixed with formalin and embedded with paraffin. 4-μm sections were obtained from the paraffin-embedded tissues. After warmed at 55 °C for 30 min, the sections were dewaxed and rehydrated in xylene and ethanol, respectively. Then, the sections were boiled in sodium citrate-hydrochloric acid buffer solution for 10 min and blocked with 1% goat serum for 20 min at room temperature. The sections were incubated with mouse monoclonal antibody to MDA antibody (ab243066, Abcam, Cambridge, UK) at a dilution of 1:100 overnight at 4 °C, followed by incubation with a CY3-labeled goat anti-mouse IgG secondary antibody (GB21301, Servicebio) for 30 min at 37 °C. The cell nucleus was stained with DAPI.

### Statistical analysis

Statistical analysis was conducted using IBM SPSS Statistics 26.0 (IBM, Armonk, NY, USA). The statistical graphics were drawn using GraphPad Prism 9.4 (GraphPad Software, Boston, MA, USA). All measurement data are presented as the mean ± SD. Survival analysis was carried out using the log-rank test associated with Kaplan-Meier analysis and the Cox proportional hazards model. Student’s t-test was used to analyse the data with equal variance, and the Mann-Whitney U test was used for those data without equal variance. A *P* value < 0.05 was considered statistically significant, and all P values were two-tailed.

### Supplementary information

Supplementary materials

uncropped original western blots

## Data Availability

All data in our study are available upon reasonable request.
