# Supplementary material for: NEAT1_1 confers gefitinib resistance in lung adenocarcinoma through promoting AKR1C1-mediated ferroptosis defence
Source: Cell Death Discov. 2024 Mar 12;10:131. doi: 10.1038/s41420-024-01892-w (PMC10933475; doi:10.1038/s41420-024-01892-w)
Supplement: Supplementary file 1 — Supplementary materials [file 41420_2024_1892_MOESM1_ESM.pdf]

Supplementary Figures

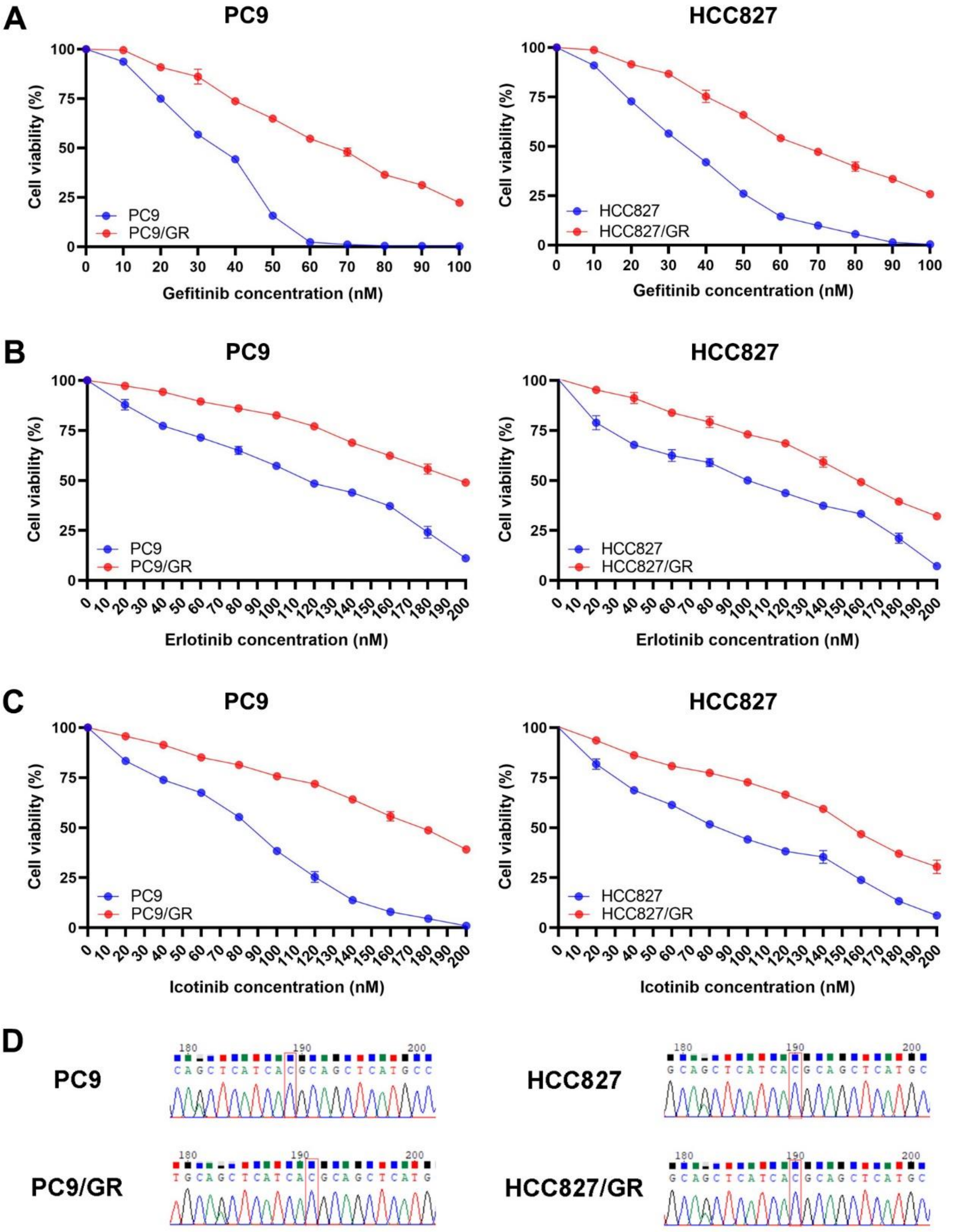

Supplementary Fig. S1

The sensitivity of PC9, HCC827, PC9/GR and HCC827/GR cells to first-generation EGFR-TKIs. A-C The sensitivity of PC9, HCC827, PC9/GR and HCC827/GR cells to gefitinib, erlotinib, and icotinib. **D** The EGFR T790M mutation status of gefitinib-resistant LUAD cells and matched parental cells.

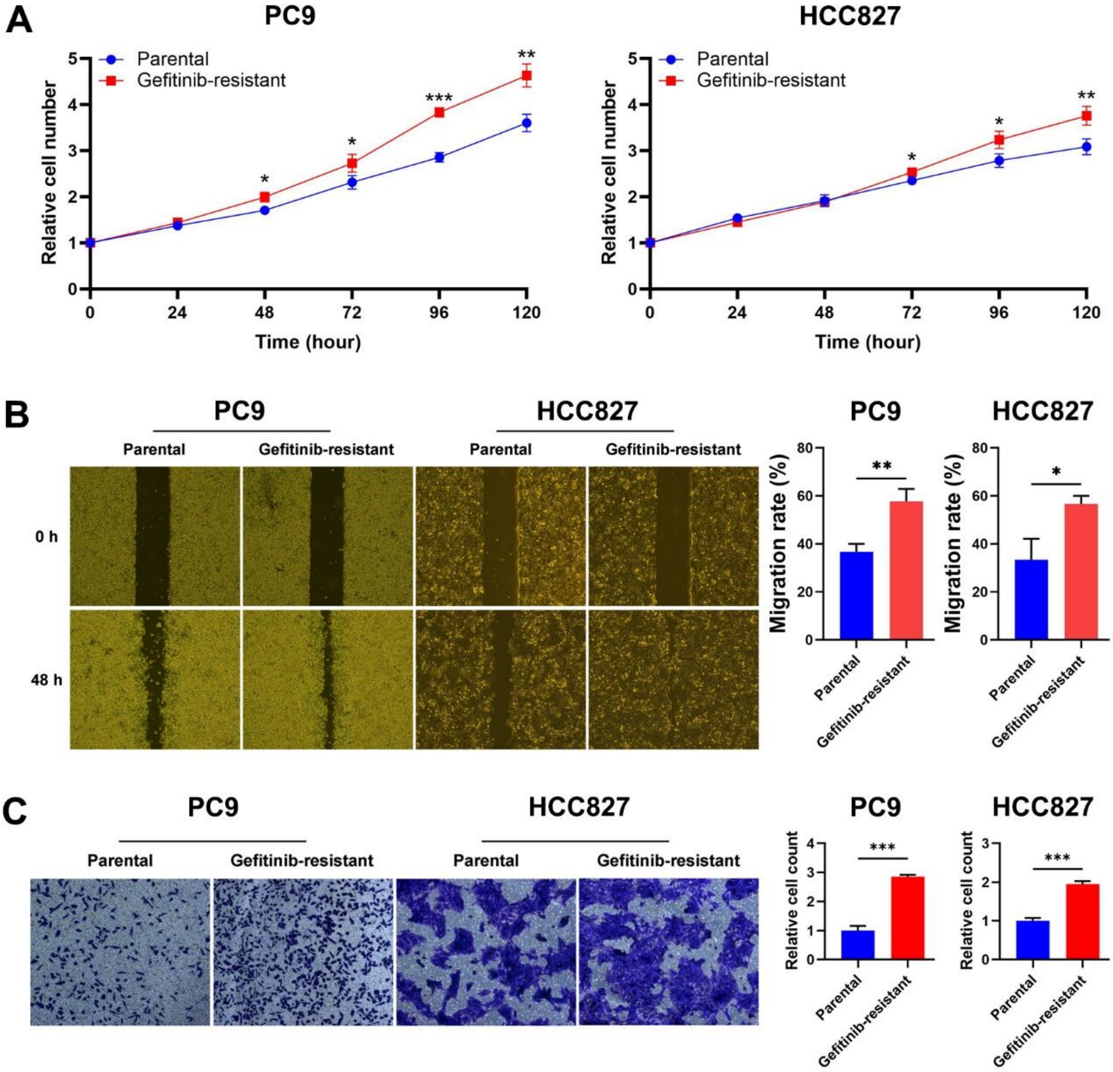

Supplementary Fig. S2

The malignant behaviors and EGFR T790M mutation status of gefitinib-resistant LUAD cells and matched parental cells. A-C The proliferation, migration, and invasion ability of gefitinib-resistant LUAD cells and matched parental cells, assayed by performing CCK-8, wound healing and transwell. Error bars indicate SD. *$P < 0.05$; **$P < 0.01$; ***$P < 0.001$.

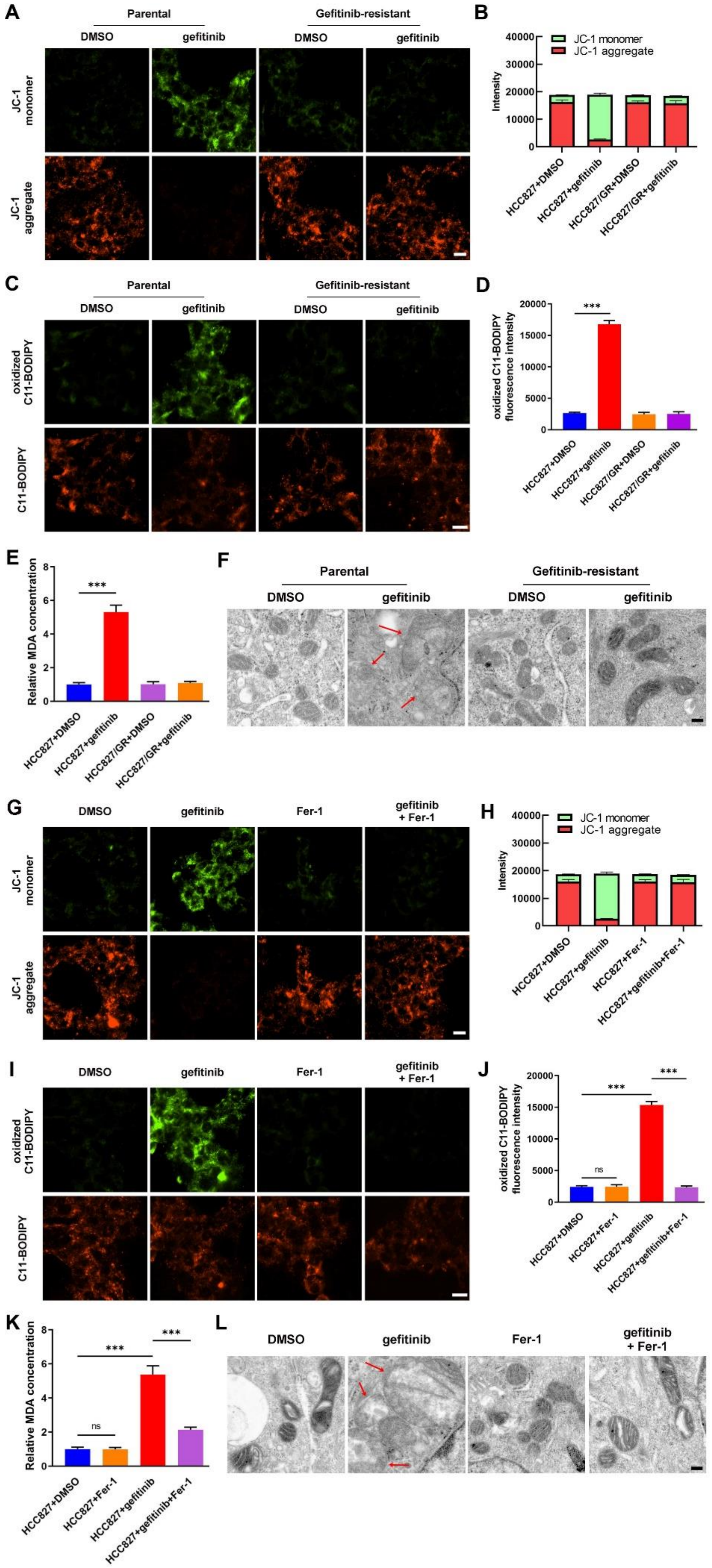
A
Parental
Gefitinib-resistant
DMSO
gefitinib
DMSO
gefitinib
JC-1 monomer
JC-1 aggregate
B
JC-1 monomer
JC-1 aggregate
Intensity
40000
30000
20000
10000
0
HCC827+DMSO
HCC827+gefitinib
HCC827/GR+DMSO
HCC827/GR+gefitinib
C
Parental
Gefitinib-resistant
DMSO
gefitinib
DMSO
gefitinib
oxidized C11-BODIPY
C11-BODIPY
D
***
oxidized C11-BODIPY fluorescence intensity
20000
15000
10000
5000
0
HCC827+DMSO
HCC827+gefitinib
HCC827/GR+DMSO
HCC827/GR+gefitinib
E
***
Relative MDA concentration
8
6
4
2
0
HCC827+DMSO
HCC827+gefitinib
HCC827/GR+DMSO
HCC827/GR+gefitinib
F
Parental
Gefitinib-resistant
DMSO
gefitinib
DMSO
gefitinib
G
DMSO
gefitinib
Fer-1
gefitinib + Fer-1
JC-1 monomer
JC-1 aggregate
H
JC-1 monomer
JC-1 aggregate
Intensity
40000
30000
20000
10000
0
HCC827+DMSO
HCC827+gefitinib
HCC827+Fer-1
HCC827+gefitinib+Fer-1
I
DMSO
gefitinib
Fer-1
gefitinib + Fer-1
oxidized C11-BODIPY
C11-BODIPY
J
***
***
ns
oxidized C11-BODIPY fluorescence intensity
20000
15000
10000
5000
0
HCC827+DMSO
HCC827+Fer-1
HCC827+gefitinib
HCC827+gefitinib+Fer-1
K
***
***
ns
Relative MDA concentration
8
6
4
2
0
HCC827+DMSO
HCC827+Fer-1
HCC827+gefitinib
HCC827+gefitinib+Fer-1
L
DMSO
gefitinib
Fer-1
gefitinib + Fer-1

Supplementary Fig. S3

Ferroptosis protection confers gefitinib resistance in LUAD cells *in vitro*.

A-F Effect of gefitinib on gefitinib-mediated ferroptosis-related phenomena, including JC-1 monomer/aggregate ratio, oxidized C11-BODIPY, MDA products, and mitochondria in HCC827 cells. DMSO was used as control. **G-L** Effect of Fer-1 on gefitinib-mediated ferroptosis-related phenomena, including JC-1 monomer/aggregate ratio, oxidized C11-BODIPY, MDA products, and mitochondria in HCC827 cells. Scale bars for JC-1 assay, 20 μm. Scale bars forC11-BODIPY staining, 20 μm. Scale bars for TEM, 0.2 μm. $^{***}P < 0.001$.

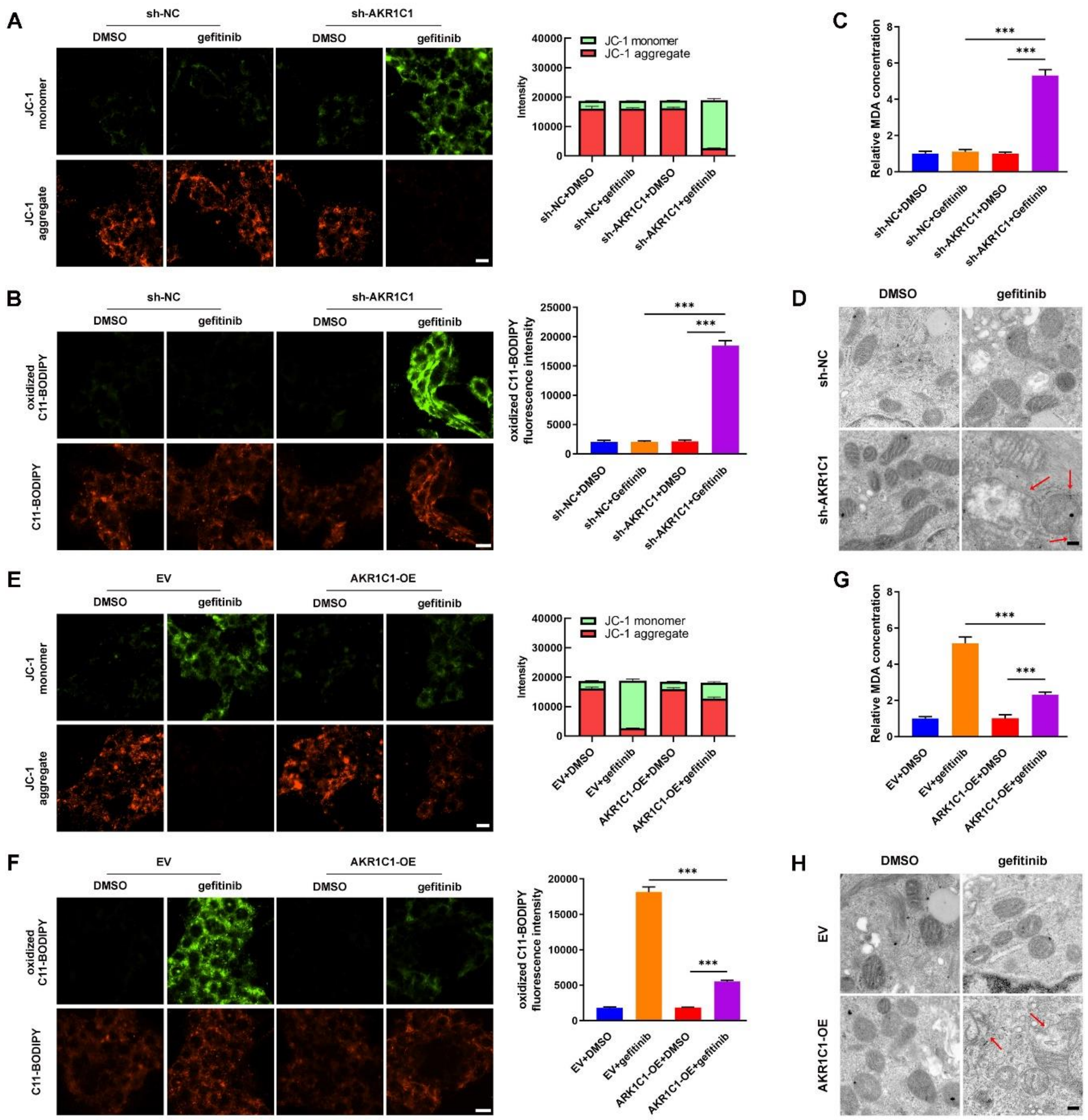

Supplementary Fig. S4

AKR1C1 contributed to ferroptosis protection in LUAD cells *in vitro*. A-D Effects of sh-AKR1C1 on JC-1 monomer/aggregate ratio, oxidized C11-BODIPY, MDA production, and mitochondria in HCC827/GR cells. **E-H** Effects of AKR1C1 overexpression on JC-1 monomer/aggregate ratio, oxidized C11-BODIPY, MDA production, and mitochondria in HCC827 cells. Scale bars forC11-BODIPY staining, 20 μm. Scale bars for TEM, 0.2 μm. Scale bars forC11-BODIPY staining, 20 μm. ***P < 0.001.

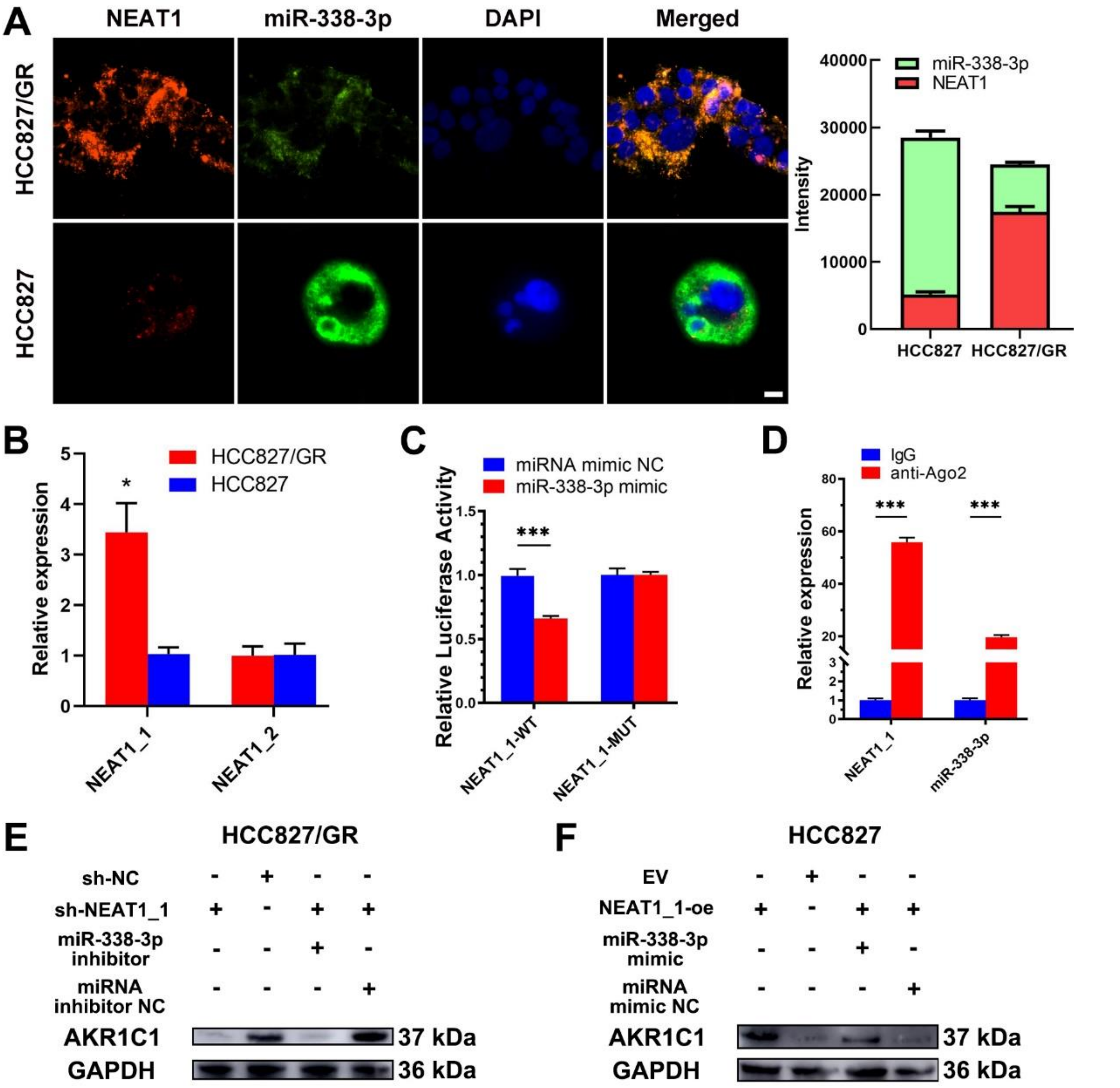

Supplementary Fig. S5

NEAT1_1 upregulates AKR1C1 by sponging miR-338-3p in LUAD cells *in vitro*. A The subcellular location of NEAT1_1 (red) and miR-338-3p (green) in HCC827/GR and HCC827 cells, assayed by FISH. Scale bars, 10 μm. **B** The expressions of NEAT1_1 and NEAT1_2 in HCC827 and HCC827/GR cells, detected by qRT-PCR. **C** Luciferase reporter assay shows the luciferase activity of the HCC827 cells transfected with NEAT1_1-WT and NEAT1_1-MUT after co-transfected with miR-338-3p mimic or miRNA mimic NC. **D** Enrichment level of NEAT1_1 and miR-338-3p in the Ago2 and IgG pellets of HCC827 cells, respectively. **E, F** NEAT1_1/miR-338-3p/AKR1C1 axis was confirmed by performing miRNA rescue experiments in HCC827/GR and HCC827 cells. **P* < 0.05; ****P* < 0.001.

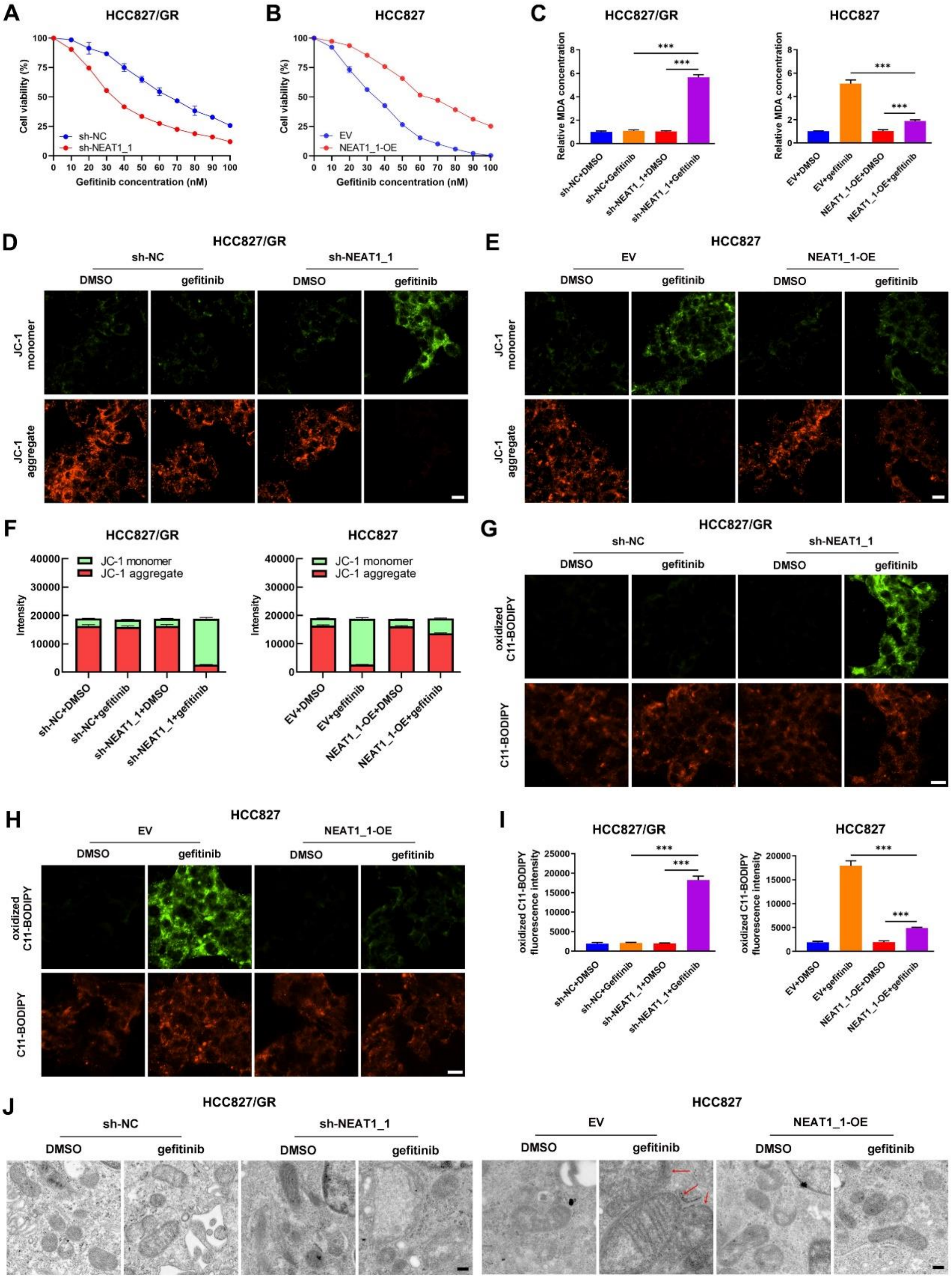

Supplementary Fig. S6

NEAT1_1 induces ferroptosis protection and gefitinib resistance in LUAD *in vitro*. **A** Effects of sh-NEAT1_1 on the sensitivity to gefitinib of HCC827/GR cells. **B** Effects of NEAT1_1-overexpression on the sensitivity to gefitinib of HCC827 cells. **C** Effects of NEAT1_1 on MDA production in HCC827/GR and

HCC827 cells. **D, E** Representative fluorescence of JC-1 monomer and aggregate in HCC827/GR and HCC827 cells. Scale bars, 20 μm. **F** Effect of NEAT1_1 on the JC-1 monomer/aggregate ratio in HCC827/GR and HCC827 cells. **G, H** Representative fluorescence of C11-BODIPY in HCC827/GR and HCC827 cells. Scale bars, 20 μm. **I** Effect of NEAT1_1 on oxidized C11-BODIPY in HCC827/GR and HCC827 cells. **J** Representative TEM image of HCC827/GR and HCC827 cells treated with DSMO or gefitinib. Scale bars, 0.2 μm. *** $P < 0.001$.

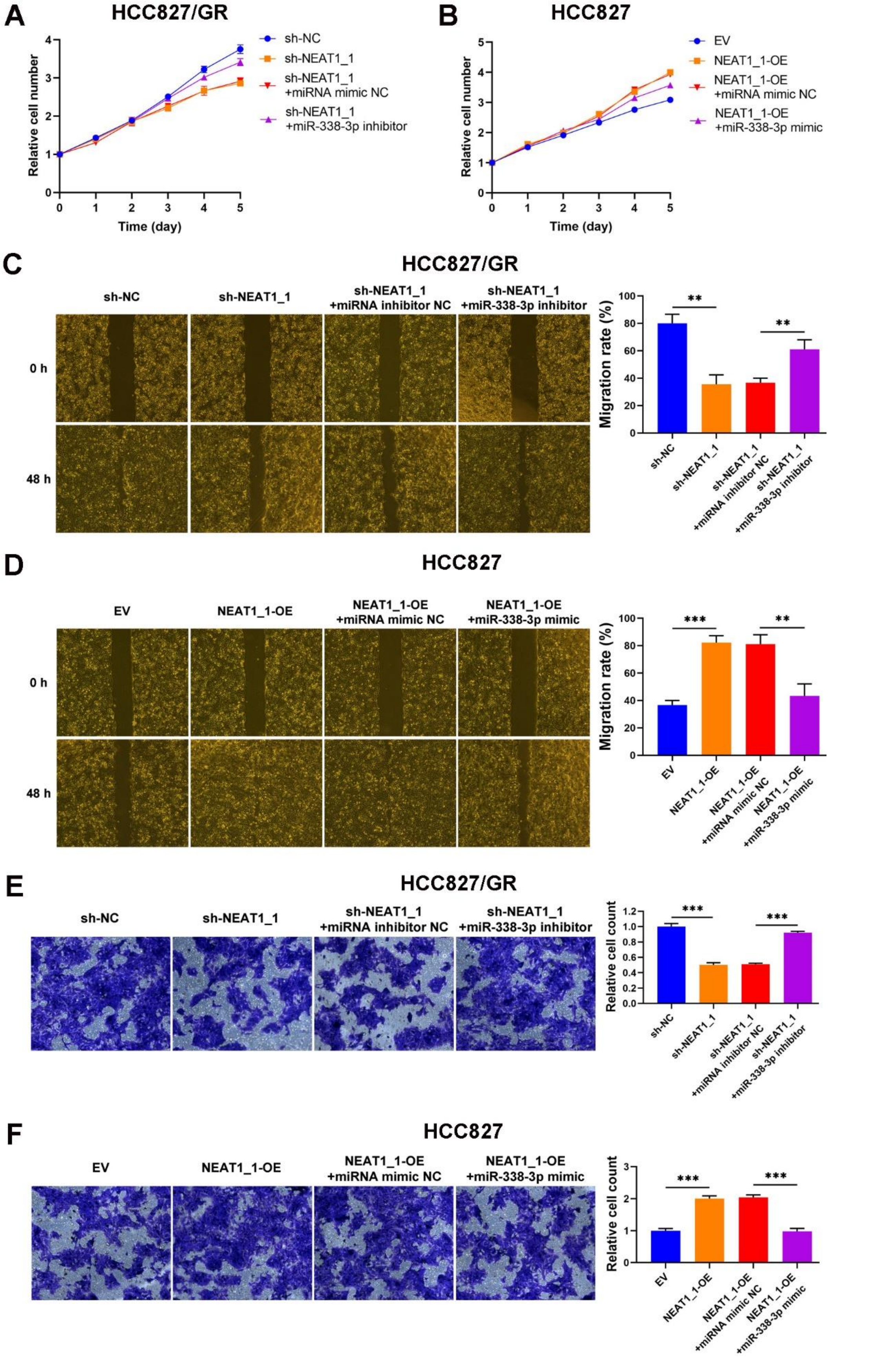

Supplementary Fig. S7

NEAT1_1/miR-338-3p/AKR1C1 axis promotes malignant behaviors of

LUAD cells *in vitro*. A, B Effects of NEAT1_1/miR-338-3p/AKR1C1 axis on the proliferation ability of HCC827/GR and HCC827 cells. **C, D** Effects of NEAT1_1/miR-338-3p/AKR1C1 axis on the migration ability of HCC827/GR and HCC827 cells. **E, F** Effects of NEAT1_1/miR-338-3p/AKR1C1 axis on the migration ability of HCC827/GR and HCC827 cells. ** $P < 0.01$; ***$P < 0.001$.

Supplementary Tables

Table S1 Primer sequences for qRT-PCR

Name	Primer sequence (5'-3')
NEAT1	F: GTGTGATCTGAAAACCCTGCT
	R: CCCAGAAGACAGAAAGATCCCA
NEAT1_2	F: GGGGCTTGAGCAAAGTGGG
	R: CACACAGTCTGAGGACTACCAGG
GAPDH	F: ACAACTTTGGTATCGTGGAAGG
	R: GCCATCACGCCACAGTTTC
miR-338-3p	RT: GTCGTATCCAGTGCGTGTCGTGGAGTCGGCAATTGCACTGGATACGACCAACAAA
	F: ACAACTTTGGTATCGTGGAAGG
	R: TGCTTCCAGCATCAGTGAT
miR-185-5p	RT: GTCGTATCCAGTGCAGGGTCCGAGGTATTCGCACTGGATACGACTCAGGA
	F: CGCGTGGAGAGAAAGGCAGT
	R: AGTGCAGGGTCCGAGGTATT
U6	RT: CGCTTCACGAATTTGCGTGTCAT
	F: GCTTCGGCAGCACATATACTAAAAT
	R: CGCTTCACGAATTTGCGTGTCAT

Table S2 shRNA target sequences

Name	Sequence (5’-3’)
AKR1C1	CCGGAAGCTTTAGAGGCCACCAAATCTCGAGATTTGGTGGCCTCTAAAGCTTTTTTTG
NEAT1_1	CAGGACTAGGTGCGTAGTG

Table S3 Differentially expressed protein-coding genes in GSE199627 and GSE169513

GSE199627		GSE169513	
upregulated genes	downregulated genes	upregulated genes	downregulated genes
ALPK2	SERPINB5	TFPI	COX7B2
PTPRD	TMC1	PYCARD	ZSCAN18
TMEM52B	PTPN20	CSTA	EPDR1
STX11	CD38	LONRF2	MAGEA4
IGFN1	KLK6	NR0B1	MAGEA12
DOCK8	GRHL2	OGDHL	MAGEC1
TMEM178B	RAB25	TRIML2	MAGEA3
ALDH1A1	CEACAM6	CD40	MAGEA1
C11orf71	SFTA2	PIP5K1B	SNCA
BCL2	TRIM55	ALDH1A1	TLR2
SEMA6A	CAPN8	ZMAT4	MAGEA9
DPYSL5	S100A14	IGF2	CYBA
GCKR	ESRP1	BPIFB1	MAGEA2B
NCKAP5	ADGRF1	KCNG1	HIST1H1A
DCDC2	LRCH2	SLC16A4	EMB
SLC17A4	MACC1	PHF21B	ZNF470
PPARGC1A	ST6GALNAC5	RAB6B	FABP5
SCUBE1	BCL11A	IRX4	CSAG1
GPC4	SCEL	SORL1	FAM9B
SHISA2	MUC5B	TDO2	KCNV1
DAW1	TMPRSS4	PLIN2	ACTL8
ACSM2A	AZGP1	NID2	GPC6
TENM3	WDR13	RORC	VCY
MYRIP	MPZL2	MAP2	NELL2
TNFAIP6	KIAA1211L	CFD	NMNAT3
CORO2B	TMPRSS11E	SLC46A3	SLITRK1
RNF182	HOXA11	GPR143	C1orf21
RAB40A	TSPAN8	AADAC	GPRASP2
DOC2B	FAAH2	BLNK	PARM1
ROBO4	LY6D	RHOU	FAM92A
OLR1	NAALADL2	ABCA12	PRICKLE2
PRLR	TMPRSS13	PYDC1	TMEM255A
LZTS1	SLCO2A1	GLI3	ZNF257
FBLN1	PKIA	SYT13	ZFP69
HMGCLL1	ATG9B	DIP2C	GALNT14
LHFPL6	AGR2	MSLN	NECTIN3
MAOA	BARX2	FOLR1	ZNF682
TUBB8	OVOL1	PCDHB9	CPS1
DOCK2	XG	ANXA10	AGMAT
LMNTD1	SLC30A1	RBM24	KCNS1
SERPINE1	C8orf31	HOXB13	EMILIN2
NPNT	PCDH1	BASP1	SNRPN
PARD3B	RASAL1	HOGA1	KCND2
NR1H4	NLRP12	HEYL	LINGO2
MYL3	KDF1	CCL3	GATM
KIF26A	MUC5AC	FOXN3	PCDH20
STUM	TRIB2	NKD2	PTGIS
NKAIN1	WFDC2	LDHD	MEIS1
SDK1	IL1RN	FBXL13	OLFM4
GATA4	TRPC6	BDKRB1	ADAMTS9
CD6	CNTNAP2	GPX2	NOVA2
BMPER	NOS3	ACVRL1	HS6ST3
SLC9C2	TP63	DEFB1	ZNF681
PSG9	CADPS2	CAMP	VIM
CABP1	ANO1	FZD10	LIN28B
PSG5	PLEKHS1	DYNLRB2	SORBS2
TLE4	B4GALNT3	COX8C	IL7
IL32	TMEM30B	RFLNB	KCNMA1
GSTA1	AGMAT	COPZ2	ACSS3
TMEM98	ACPP	CLDN8	SMO
KCNE1	NECTIN4	SLITRK6	CASP14
MAF	CELF2	CBFA2T3	ZNF493
FOXD3	FXYD3	ADRA2C	CYP24A1
ATRNL1	CDH3	NPTX1	ITGBL1
TNFRSF19	GSDMC	SLC47A2	CILP
SLC2A2	NKX1-2	CCL26	MMP7
CHST9	CLEC7A	RASGRP2	PDE3B
PDGFRB	MARK1	ID2	LEF1
MUC2	CFH	CDH2	XPO4
ALPL	FGFBP1	SYNE3	GNB4
UGT1A1	NUPR1	CLIC3	PLAT
THSD7B	FREM1	SLC44A4	ADRB2
UPK1B	CAPN10	TNFRSF10C	PIWIL4
HCN4	EPS8L3	COL4A2	MCTP1
IGFBP7	COL17A1	ST6GALNAC2	BBOX1
PSG8	AP1M2	SERPINE1	RARRES1
PSG3	EHF	CACNA1H	FAXC
UGT2A3	CHI3L2	ATP8A1	ARHGAP22
NPR1	CNTN1	ERO1B	GUCA2B
BEND5	CHI3L1	EFS	MGP
ERO1B	HHLA2	CCN5	IGFBP1
CYP2C9	PLA2G4F	DHX58	SLC24A3
JAKMIP3	PRODH	LY96	TGM5
AKR1C2	DLX3	SLC7A8	RASGEF1B
RFTN1	PALM3	SLC40A1	CHRDL1
RIPOR3	TNS4	DEFB103B	ZNF556
GSTA2	AOC1	ACOT4	PTCHD1
FNDC4	PROM2	MUC15	IL6

CNN1	TMC5	MCMDC2	TMTC1
CD82	PLCB2	KLF13	KCNH8
LAYN	POMGNT1	NLRP10	MCTP2
GSG1	PTAFR	ADAMTSL4	ZNF93
AFP	DAPP1	TMEM200B	ST3GAL6
SLC9A9	PIGR	ARHGAP44	TNFRSF11B
JPH2	TLDC2	C5orf38	COLEC11
CHL1	TMEM239	RNF32	PADI1
PSG2	ATP10A	FOLR3	M1AP
ABCB5	HGD	GDF15	GPR63
CDH2	WNT9A	AK7	MAGEA8
CA4	SH2D1B	PCDHB14	CHRM3
SLC17A3	MDFI	FUT2	FMO4
STOX2	TSSK6	PLPP7	FAM83F
DPY19L2	ADGRV1	RGS7	BHLHB9
N4BP3	SVOPL	EEF1A2	PLBD1
PCYT1B	TJP3	H2AFY2	SIX2
PTH1R	LMO7DN	RTN4RL1	ALOX5AP
ANTXR1	MUC4	TMCC2	TMEM163
PHF21B	ZBED2	ULBP1	KRT1
SERPINB10	MYBPH	SLC30A3	SGPP2
FLT3	ANKRD22	KCNH2	LCP1
ADGRL2	MMP13	GSTO2	ZNF385D
C3	ADAD2	ELOVL3	PLAAT1
PODXL	RASSF9	IL17C	ADGRV1
SLC25A31	C1orf210	ECM2	FAM184A
PSG11	TMEM125	FMO5	CYP7B1
UNC5A	ST14	PLEKHA4	AASS
KCNQ1	ABO	BAIAP3	TNC
VNN2	ACOX2	ZNF610	NRG2
ILDR2	EPHA1	LHX2	TNFRSF8
EBF3	CHIA	DDO	SLC16A9
NTRK1	CD79A	ELL3	CXCL10
TLR2	ZNF705G	PKD1L2	ICAM2
MAP6	NMU	BST2	IL17D
PLCG2	CREG2	LYZL2	CCK
NDRG2	SCNN1A	GYG2	NIBAN1
CYFIP2	BSPRY	NFE2	NNT
TMEM229B	BICDL2	RHBG	SAA1
RBPMS2	DTX4	EGR1	RAMP1
CERS4	DUSP6	ADAP2	ASB2
FGFR1	SAA4	TLE4	GKN1
ANKRD1	ST8SIA4	CES4A	GAL
ERH	ERP27	MORN3	FAR2
SNX10	SEC61G	STARD5	SERPINB3
FGR	EGFR	TRIM50	XAGE2
TNFRSF13C	BAG5	CCBE1	IGFBP4
OSBPL6	ADGRG2	ZNF750	SCNN1G
COL4A1	EGLN3	ZC4H2	TMEM198
SRCIN1	FBN2	SLC23A1	LAPTM5
KRTAP5-1	CFAP47	TRPC4	PDE6H
LRRC32	CYP2C18	GLT8D2	CLMP
PSG1	GPR87	C19orf18	NKX2-1
B3GNT2	HAS2	STAT4	ADAMTS3
SIM2	TMC4	SLC22A11	HMGA2
SIX3	FAP	AKAP14	KRT77
ZNF697	WNT7A	KISS1R	VSIG2
BDKRB1	SLC2A3	PTGIR	TMEM158
VCAM1	IL13RA2	DMBX1	DCDC2
MORN4	TSPAN1	LEMD1	TCP11
PDZK1	PRR15	GPR137B	CCN3
DAB2	RP1	COL9A3	NEK3
CAMK4	TBC1D30	RPS6KL1	TRIB2
THBS1	CD244	MSMB	KL
RNASE3	SYNPO2L	IGDCC4	TNFAIP3
IQCA1L	ARL5C	GNG7	FOXI1
PAPPA	HLA-DRA	PTGS1	ZNF100
OR2G6	MUC16	ZNF214	RNF212
THBS2	MAP7D2	HPGD	SLIT2
PRSS35	WBP2NL	CASC1	LRRTM4
CRYAB	MAFB	ATP2A3	NFAM1
SIX2	CALB1	IFITM1	ZNF354C
EPM2A	PDX1	TENT5C	SFTPB
DBH	FGF9	FAM124A	SOX7
LPAR3	AIM2	SGK2	C6orf223
CREB5	ALOX5	BMP2	SPHK2
PAX2	FAM111B	GLI1	PDLIM4
MATN2	IKZF3	SLC6A14	LRRCC1
ADAMTS7	ADAM28	ASNS	MARCO
HPD	EPS8L1	WDR78	HSPA4L
IL10RA	SHROOM2	NLRP7	ACTG2
FBXL16	GNGT2	BEND7	PLCL2
DACT1	NRXN2	ATP1A2	SESN3
COL12A1	CFHR1	CNNM1	TCF7
SHE	TSHZ1	GLDC	ANKRD7
ANXA6	MKX	CDKN1C	KCNB1
BOC	C1orf21	PLPPR3	ANGPT1
NTSR1	CYP24A1	PRPH	DISC1
PSG4	ZNF467	AGPAT4	CSRP2
RETREG1	GUCA1A	TPPP	CYP3A7
ARHGAP23	IL1R2	ANKRD34B	ACSL5
CECR2	TMEM200C	LRRC32	NR2F1

PRR26
EVI2A
SPNS3
HECW1
MRAS
CA2
FOSB
ATP2A3
PCDHB15
PARP11
PRODH2
ACTR3C
BACE2
PIK3R5
IGDCC3
FAM20C
SARDH
KRT17
SIPA1L2
TSPAN7
GMNC
RENBP
LRIG1
TMEM171
LRRC36
WNT5A
ZSWIM5
LRP3
KCNQ4
GPR171
NPL
PDGFD
SLC4A4
CMTM2
B3GNT7
SLC22A17
NKD1
ERICH5
TSPAN12
SLC2A5
TRPM6
MGAT1
AMOT
ELFN1
C7orf69
PSG6
ZNF704
LIX1L
TMEM121
MUC17
DHRS9
LIMCH1
TMEM163
ELAVL2
SLC22A15
MAPK4
PHOSPHO1
HUNK
FAM78A
SHC4
MDGA2
ROBO3
KIF5C
LPAR1
GYS2
LRRC63
IL7
BCL6B
FOLR3
ACSM1
MYL9
TIE1
SLCO4C1
EBI3
ITIH3
PLA2R1
ELOVL3
FAM124A
PRCD
KCNE4
CYBRD1
ARL5B
HNF4A
ONECUT1
SLC25A27
ANKRD7
AKR1C1
REEP2
MSI1
CYP39A1
CNGB1
SLC7A5

PAPPA2
RSAD2
TCN1
CR2
SDR16C5
EFEMP1
SERPINB4
TXNDC2
LUM
CYP4F3
MAL2
ASB12
SYK
AQP5
EFCAB8
GHRHR
CLIC2
ST8SIA6
OPHN1
CDH17
SLFN11
MAG
COX6B2
EPHB3
VIL1
CTNNA2
SLC22A31
C17orf64
HABP2
CST6
ADH1C
PPP1R14D
MYH16
ENPP2
GDPD2
SGPP2
HCAR1
WFDC10B
KCNG4
ANOS1
MAGEA5
LGALS7B
FOXP2
HCAR2
MERTK
KCNQ3
FAM83F
TUB
CRABP2
MYCL
ADGRF4
TMEM176B
SLC2A7
FBXO27
GFRA2
JPH1
ENPP3
MSLNL
WDR87
KHDRBS3
FOXA2
SLC10A2
LUC7L3
HHIPL2
VOPP1
IQANK1
EPCAM
NCF4
CXCR6
TMEM156
MAGEA6
LPAR5
LRP1B
KLHL11
WNT16
PECAM1
ERMN
OR2AT4
ALOX15
CFAP161
HOXC11
DUSP2
SULT2B1
OLFM4
TNFSF9
KLRC1
IGF1
CD226
RAP2B
AIF1L
GLI3
KRT13

KCNAB2
IL11
GATA5
TMOD2
TMEM98
LY9
ABCA7
OSBPL5
RAB26
IRX2
PODNL1
WNT5A
HPN
ZNF30
AKR1C1
AKR1B10
EFCC1
SLC5A10
ADGRA2
CSRP1
TRPV2
ZNF597
ODF3L1
PAQR9
MAP1LC3C
RPRML
FOXF2
MME
EPSTI1
PCED1B
CATIP
POF1B
ECM1
SPATA6
RGS9
LRRN2
ZNF382
BIN2
RASA4
MPP1
ANXA1
ENDOV
ZNF287
ACSM1
MDGA1
SLC12A5
TTC25
MOGAT1
CRIP1
C9orf24
EGF
PLAC1
FAM133A
ENHO
FAM78A
CCDC74B
RNF183
KCNQ1
CD19
MED12L
RDH12
CHAC1
MAPT
ZSCAN4
CLGN
FAM83E
EPHB3
ADHFE1
PDZD7
TNFRSF14
IRF7
ISL1
FLRT1
EFHD1
SPINK5
P2RX7
RAET1E
GPR20
TNNC1
CBX8
ZNF446
ARC
RPH3AL
CABYR
CYP4F11
FRS3
OLFML2A
HBE1
CATSPER2
EXD3
SARDH
AGBL2

EGR4
THBD
PAG1
EVA1A
TFRC
COL6A1
ZIC5
ZNF385B
LPO
SSTR2
ZFP28
TMPRSS2
COL27A1
CXCL11
DGLUCY
SRSF8
ST3GAL5
NR3C2
MTFR2
SLC16A10
BIRC3
SULT1C2
HOXA2
CIART
WNT11
MYB
CXCL6
SLC35G2
KRT23
DISP2
GUCY1A1
CARD16
KRT5
ADAMTS12
RERG
LYPD1
VIPR1
SLC1A6
CALML5
DHRS2
LRIG1
PI3
EDN2
ADGRF1
EYA1
SAA4
SPRR2B
B3GALT1
PANX1
CDC25A
DSEL
FAM170A
PTPRG
EVC
FDCSP
PIP
ENPP1
SSTR4
GBP1
FKBP7
DACH1
NAT8L
SLCO4A1
PTGER4
GJB2
RAG1
PDE8B
B3GALT6
NRROS
LOX
SPOCD1
RPL27A
KLHDC8B
ANTXR1
TNIP3
GPR31
TPM1
KRTAP3-1
RPL7AP54
SLC15A1
TGM2
ENOX1
FST
MMP16
FSTL1
TRPV4
OXNAD1
CAMK2N2
MFHAS1
DUSP7
KCTD14
LRRC8C

GNG12	IFITM1	TTYH1	PKIA
MMP9	RTL9	DIO2	TRIP13
FILIP1L	VCX3B	EPHX1	ANPEP
SLC7A8	RAB19	ISL2	CST1
RBP7	C1orf116	ZFP3	PDK1
PTPRO	PELI2	SERPIND1	PWWP3B
DPF3	F11R	LST1	GCFC2
SRRM3	PCDH9	BEST1	QPRT
FSTL5	CD164L2	LPAR6	KLK15
ARHGAP31	CACNA1I	PKN3	COL6A2
FAM189A2	LANCL2	FAM167B	RCAN2
FLG	FAM83A	HYAL1	NCAPG
HTRA4	CRYBG1	CCDC170	NOG
DUSP8	PPP1R1C	TUBB8	PIK3R1
ACSM5	DLG3	NOXO1	SYT14
TNFRSF1B	JAZF1	F2RL2	IRAK3
KCTD4	HOXC12	KRT83	VNN1
SLC26A7	NR2F1	BFSP1	TIMP3
FAM78B	SOWAHB	CTAG1A	NRIP3
CLDN2	NT5E	WNT10A	BPGM
IL6	RTL8B	GNGT1	ADAM19
APOC1	RPP25L	MYL9	TRMT10A
FGF22	MARVELD3	IFT46	SLC49A3
FBLN5	LRRC74B	MEIOB	ESS2
PPP1R3A	WNT5B	TRIB3	GUCA2A
NRG2	ACSL5	C1orf115	ZNF665
DAND5	CYP26A1	TNNC2	CIITA
CASQ1	YBX2	RAB3B	COL12A1
DNAJC6	ZNF404	SH2D3C	MPP6
CLMP	B3GNT6	ALDH3B2	ATAD3A
EDN2	KIAA0040	ARHGEF25	DAW1
SH3RF3	IL24	TMEM86A	CNN3
ZEB2	TNFSF8	CYP4B1	ALDH1A3
GSTM1	ERRFI1	FBXO17	CYP7A1
IPCEF1	THBD	SCN1B	NUS1
OGDHL	NKX2-1	RDM1	ERI1
CBX2	SYNE4	MLXIPL	WLS
KAZN	LRRC10B	ABCG1	SLC6A8
CPNE9	DDX11L8	CDKL3	LHFPL6
SPP1	AQP2	AJUBA	ESX1
CD1D	ANKRD60	TNNT2	INA
CPQ	IFI27	RAB9B	PLEKHF1
ABCB1	ZNF627	TCF7L1	NETO2
ALOX5AP	CYP27C1	ADCY5	RAPGEF4
PLA1A	EDAR	PAPLN	TRMT61A
GLP2R	ALDH1L1	SLCO2B1	PARP8
AADAC	USH1G	TMEM187	CPLX1
DIP2C	AMY2A	PCDHB16	LRRK1
GPR179	TENM1	FES	TMEM117
FYN	PIWIL4	SOWAHA	HDX
OLFML2A	LRP10	CNTFR	SH3BGRL
NTNG2	DRP2	SHROOM2	HS3ST1
RAB32	VSX1	TMEM105	EFNB2
C1QTNF4	SLC16A2	ATP6V1B1	TSSK2
ADAMTSL4	PEG13	PSTPIP2	HPDL
MCTP1	TLE2	HBG1	BDH1
SERPING1	AQP6	LRRC56	OR1A2
MDGA1	HLA-DRB5	PELI2	GLYCTK
GLDC	DDX25	KLHDC1	ADORA2A
TLN2	ATP8A1	CHRNA9	CENPJ
DIRAS2	S100P	CELA1	POU3F2
C4BPB	PLCZ1	RIPPLY3	AKAP7
PGRMC2	KLHL6	PCK2	GNAT2
C3orf80	MYO1D	HEATR4	KRT13
EBF4	OR2B2	ZNF528	SLC1A3
PDE7B	DNAJC15	RHOBTB3	NMNAT2
AQP11	ARHGEF38	LCA5L	CYB561D2
CDKN1C	INPP5D	PTGR1	DAPP1
SYNGR1	PTPRU	DIRAS1	MKI67
PTPN7	SPINT1	TSNAXIP1	GCKR
IGFL2	CDH5	PDGFRB	LETM1
GCNT4	GNPTAB	SCEL	EPB41L2
MCFD2	SLCO2B1	GCNT4	PSME4
OLFML1	GJB2	ADAMTS14	HOXB9
UNC13A	LRRC6	TMEM270	CDH23
PDGFB	IL18R1	CRIP2	CSF2
CITED4	CGN	COL4A3	CDCA7
PRB1	GRHL3	ZG16B	CEACAM6
TRAM1L1	MUC22	IL6R	UST
PLK2	ITGA2	SRCIN1	FBN2
TPSG1	FAM49A	YPEL1	CYCS
SLC52A1	ZNF165	LURAP1L	GPAT2
NOD2	ACMSD	BMPR1B	NREP
BVES	ADGRG7	AOC1	XYLB
FAM131B	GALNT5	IGFBP2	TNFSF12
SHC2	WFDC3	PMP22	PDSS1
CCDC170	SPRR3	UPK2	GPC4
ATP6V0A4	CDHR4	KLB	FBP1
KCNN1	MUC13	SAMD9	FAM76B
TCF7L1	RRAGA	NAT16	PLXNA2
SLC46A3	SLCO1C1	NOVA1	ZNF326
MAP1B	LSP1	LTC4S	NT5DC2

GAL3ST1	LCP1	DNAAF3	IL23A
WIPF1	KIAA0319	SNX22	NIM1K
ANKRD62	ACP7	NCF2	ICOSLG
CXCL8	AQP3	GUCY1B1	HSPA8
RHOU	AMY1A	NEK11	MAB21L2
SH3BP4	SERPINB3	KRTDAP	ADIRF
MRPL45	SOCS2	SPATA9	GEN1
CHST2	HRCT1	DTX3	ALG14
KDR	GSDMA	COQ10A	SEH1L
PCDHB11	P2RX7	PRAF2	ATAD1
CYB5R2	ACTRT3	LHX5	HSPH1
ZNF181	HEATR4	SLAMF7	CLSTN2
PPP2R2C	XKRX	F8	DOK3
HOXD9	STRADA	ATF6B	TOMM40
LRFN4	IGFBP3	C5	BRIP1
FAM83C	CMPK2	RHOXF1	A2ML1
GLRB	IFNA1	PRTN3	MAB21L4
TUBB2B	FBN1	TMEM61	CMTM7
ANK2	CACNA1D	CNGA1	FGF12
CYP3A5	TPPP3	GP1BB	EPHB6
SMLR1	PDE6B	GABRB3	TMEM71
HLX	MMP10	NECAB2	SLC2A13
ECM1	LEMD1	SPON2	IHH
RET	ARF4	CDRT1	KRTAP19-2
PGF	SLC22A18AS	GLS2	KIF7
ARHGEF40	WFDC13	CCL28	SQLE
GALNT1	RORC	CYP4F12	PALD1
C2orf16	ADAMTS20	DRC3	TYMS
ARF6	USP44	NAT14	CEACAM7
RASD2	KLHDC7B	TTYH2	SPRY1
SCNN1B	MMP28	SCIN	CMTM3
CLDN1	BMX	HCN4	MPHOSPH8
IQGAP2	NR5A1	SIDT1	TMEM171
SIRPB1	KLRG2	C1S	KRT2
C1orf143	LRAT	NPAS1	LOXL3
ICAM2	AMY1B	COL4A1	AK3
SUGCT	PCDH7	GDAP1L1	FAM136A
MCAM	SULT1B1	CALHM2	LYAR
ACHE	SHC3	CGNL1	ITGB6
DUSP1	GNE	CFHR1	TRMT10C
MIOX	RERGL	SMARCD3	CKAP4
LIMS2	HMGA1	RNF182	RFT1
LACTB	SBK3	PCDHB10	AKAP12
METTL11B	LPCAT2	KMO	NRG4
SRPX	DNAH6	MAPK8IP2	STMN3
ITGAM	CCL26	IFI27	ZGRF1
AK5	FREM2	SLC7A11	OSR1
WIPF3	DAZAP2	ZC3H6	GLUD1
RNF144B	GABRQ	NDUFA4L2	ANKRD49
WNT11	GPR143	SIRT4	CSF1
PTPN12	LURAP1	CD52	MMS22L
LIMD2	OTOGL	KCNJ12	C3orf52
SCD5	KCNF1	SYTL2	SLC18B1
KCP	DGKB	TMC6	PNO1
IGSF10	BLK	FBXO2	RIPK4
SOCS7	C20orf197	ZNF397	GRPEL1
SERPINF2	TMEM70	PDE6A	FAM53A
DDX52	LRP2	PLA2G10	KLHL18
ADPRH	ETV4	PCDHB11	DZIP1L
ACSL6	MILR1	PAGE2	ZNF677
RNF144A	FAM81B	HMGCLL1	MELTF
KRT34	ABCA12	MFSD4A	ARFGEF3
CFAP221	PDCD10	ASAP3	GABRP
DES	TEK	VN1R1	ZNF519
UAP1L1	ANK1	ADPRH	SLC4A7
DYSF	MRGPRX2	IFI27L2	HECA
IL12A	NMNAT2	CTSF	NCBP2
RCN3	LRRIQ1	L3MBTL1	SPG7
FSTL1	B3GNT3	KIAA1324	GAS1
ARHGAP40	BMP4	SCML2	SPDL1
CA12	SAMHD1	PRR16	ZDHHC2
PRKN	SLC25A47	UPK3A	KDM4D
RHOB	PLXDC2	CRLF1	NUDT15
C1orf229	FUT2	GPKOW	HMGCS1
MYOM3	MAN1C1	TRIP11	SHLD2
SERPINE2	IGF2	CA12	MCM10
CRMP1	SLC16A14	CD72	ODC1
TLR4	PLD5	REM2	NANOS1
AKR1B1	S100A1	ELMO3	PECAM1
RBM24	FAIM2	CD36	CST2
IL1A	SPRY4	C8orf31	HDAC2
PNCK	NTNG1	CCDC110	NDUFAF6
ADAM12	NT5DC4	SMIM14	GLUD2
SULT1C2	CCDC187	PNMT	RAI14
ANXA4	TCHH	LETM2	DSCC1
PTX3	FOS	SYT8	KLF9
LIPJ	SLC2A9	SLC4A11	BRCA2
DOK7	BICDL1	METTL27	E2F2
PLA2G4C	F5	SLC9A1	MRM1
RASSF8	EMP1	TSSK3	PRSS36
CYTIP	SH3BGRL	ADGRG7	AASDHPPT
KCTD12	NBPF6	TIMP2	SLC10A2

MPP4	SPAAR	FANK1	GDAP1
HBEGF	RBP1	ARL4D	IFNGR1
RRAGD	BCL2L15	TET1	YKT6
ITPKA	RDH12	DDIT3	MACC1
SMOC2	MBOAT1	DOCK11	OPA1
COLGALT2	MALL	RNF112	SESTD1
TMEM167B	PTPRN2	TMEM121	KIF5B
CSRP3	TMPRSS2	IFITM3	COLGALT2
CD177	PTK2B	GALNT18	TRMT13
SP140	GRB7	ULBP2	FKBP4
GMPR	HOXB9	TMC7	HAUS6
SLC3A1	CDS1	CLEC11A	IL6ST
HMOX1	SLCO1B3	PRRX2	SPNS3
ALX4	SLC5A12	LY6D	IL7R
ANG	FUT3	SGCB	SLC25A32
COL4A2	LHX9	KAT14	PUM3
SIRPA	ETV1	SEPTIN6	CTSC
AVPR2	GJB7	BBS5	CEACAM3
CPA4	FAM178B	CERS4	YES1
S100A3	FOXR1	FOXD2	ZNF71
PIK3CD	PRRT4	DLK2	PAX6
PLEKHH2	HMGN5	TMC4	S1PR2
GOLGA8F	DENND2D	ELMOD1	GNL3
WDR91	MYLK2	PARD6G	CDC7
RUNDC3B	MPZL3	ASPSCR1	SLC35B2
MEFV	P2RY6	TGM1	ZNF429
NUAK2	RNF217	VAMP1	ARMCX2
COL13A1	FAAH	ARL11	MRE11
ASGR1	IRF6	CORO2A	PDE12
GOLGA8M	SCN4B	TTC21A	LCE1E
SPX	ARL4D	HOPX	RFC3
CD83	EOMES	CAMK2D	KRT32
CCIN	PPEF1	RBM43	SIK2
GNG4	SH2D4B	ABCC2	ZDHHC20
SYTL5	DMRT2	CDA	GPR75
COL5A1	TMEM225B	KCNF1	GDPD4
CYP4Z1	BHLHB9	G6PD	KDM1A
B4GALNT1	RNF150	HPD	EREG
GP1BB	STEAP4	KLC3	RAB42
INPP5J	PCSK1N	HIST2H2BE	DCTPP1
NFIX	ITLN2	DCP1B	DCBLD1
EGFL7	PRSS8	FOXN4	CORO2B
KCNMA1	MMP1	TSC22D3	PTRH2
FMNL1	CEACAM16	ONECUT1	HSD17B7
ZNF473	SESN3	CYP4F2	RBFA
DGAT2	VGLL3	GLIS1	ZNF267
FNDC10	EDNRA	LMCD1	SSTR1
GABARAPL1	OAS2	CYP4X1	P2RY6
LRRC61	SCARA3	TRIM46	UBP1
TNFRSF9	LIPG	GAA	CDCP1
EML1	SRPX2	TRIM7	ABCA11P
ADAMTS6	GPX2	LRRC75A	HSPA14
ASPHD2	FAM95B1	KREMEN1	GPX3
PDGFC	ACTN2	ALDOC	CANX
DCLK2	RAB11FIP4	KRCC1	MSX1
IL1B	MAP2	PTPRR	FADS2
PNPLA3	ST6GAL1	RBCK1	MOGS
TSPAN33	IGSF9	PRKAB2	COL13A1
RILP	KRTDAP	HTATIP2	SUV39H2
TRIM36	UNC5C	KNDC1	C1orf174
NECAB2	ISM1	L1CAM	IRAK2
ARID5A	CACNA1A	ZMYND12	PTPRJ
KLHL4	FGD3	DKK3	CENPO
KIAA0930	NDUFA4L2	EPS8L2	NAA38
SHH	LAMC3	JSRP1	ECHDC1
CREB3L1	NLRP2	LRRC36	LOC440864
TRIM9	PPP1R1B	IL36RN	PXYLP1
MEGF6	COLCA1	CLDN3	VSTM1
OLIG2	CAPZA3	ANXA9	ITGA6
FAM221A	SLFN12L	F7	SACS
FGF5	ZNF474	GLP2R	SPANXA1
BMP5	IGHG2	AGR2	AQP2
TRPV4	SLC37A1	MITF	LCTL
RFLNB	PPP1R14C	FAM81B	ZNF254
COL16A1	SIGLEC10	ZFP37	LRCH3
GPR137B	CDC42BPG	TUBAL3	NFE2L3
MAGI2	HPGD	TEX19	AHSA1
SPNS2	HSD17B2	LMO1	MCM3
NNMT	RCOR2	CDKN2C	HDDC2
BRSK2	CKMT1A	CLUAP1	FEN1
HAVCR1	LSR	CCR10	CABLES1
ADAP2	ZC3H12B	FGFR3	SASS6
RASGRP1	DPEP1	ANKRD46	STEAP3
TMEM74	SYNPR	CCDC136	CHORDC1
CACHD1	LGI4	BCL2L14	NCF4
RSPO3	PTPN22	ZNF544	AMD1
KIF3C	CD14	KLF2	MRPS30
DLC1	MROH6	ZFP82	USP14
GPR63	CD96	KCTD6	PIGW
GPRC5B	PADI4	ABCA4	RTP4
ARMC9	SLC6A20	EPOR	SLC5A3
COL6A3	CYP2B6	CLIP3	ZNF488

TPTE2	MAOB	HIPK4	OR2B11	
TAGLN	CHRM1	FUT1	E2F7	
NLRP1	CCNI2	HYI	ZFP91-CNTF	
MYH9	NKX2-8	ABAT	DDX10	
FARP1	B3GAT1	ACACB	ART4	
ERVH-1	RARG	CLU	CACNA1A	
GLIPR2	EXPH5	NUCB2	TTLL12	
PREX1	MST1R	CBX4	TMEM126B	
GGTLC2	COL21A1	ZNF81	SMCO4	
TTLL7	TRPC4	GMPR	ZNF675	
TMEM132A	MISP3	EFCAB6	MANF	
F10	ADCY8	IDNK	ERICH1	
SELENOM	MMP2	CDH15	MRPL57	
RASGEF1C	KCNK3	PBXIP1	GOLIM4	
NTN3	UNG	SCARA3	FFAR4	
FNDC11	KRTCAP3	DAPK2	RNF168	
CCRL2	PDE5A	PREX1	SLC25A15	
GRIK4	C7orf57	CELF5	NEIL2	
RFPL4A	POTEC	HDDC3	SRM	
LMO4	EPB41L4B	SELENOM	RANBP6	
OLFML3	CYP4F12	ZNF613	NUFIP1	
ANO2	TTC6	OR10A6	SRGAP3	
CNKSR3	NUP62CL	WDR45	C11orf45	
SMTNL2	CTNNBIP1	SYNPO2L	OPA3	
JAM2	IGFBP2	ZNF324B	ALDH1B1	
ECM2	PCSK6	RGL1	SVIL	
ELOVL5	TIMM8A	DSTYK	KYAT3	
NPPC	APOL4	EPHX2	PIGG	
HRNR	CDHR2	MOB2	DCUN1D5	
GAREM2	MYH14	FAM189A2	TBC1D21	
AATF	CMTM4	MAN2A2	TMTC2	
DLX1	SIGIRR	SNPH	HNRNPAB	
LHX2	STAB1	CCDC106	PARVB	
GUCA1C	SBSPON	FAAP100	DROSHA	
DYRK3	LAD1	SPEF1	ATAD5	
GSTA4	FGD2	ZNF211	ARHGDIB	
GPR161	ARHGEF15	CABCOCO1	MRVI1	
CADM4	CDH1	BBOF1	PALMD	
C9orf40	SELL	SLC22A18	PCSK5	
SPOCD1	FIBIN	MAFB	OAT	
FABP1	OVOL3	CPXM2	PRPF38B	
PDE1C	SULT1A1	ANKK1	MAD2L1	
P4HA3	ANXA9	GADD45G	OR52I1	
RWDD2A	EIF5AL1	FBXO15	DCAF13	
FN3K	PLA2G10	AGT	SRI	
SFRP4	PTGER4	STARD8	SLC12A2	
TNIK	PLSCR2	NEURL2	FANCA	
ASB18	LCK	DOK7	ZNF143	
WDR63	SPP2	PRX	PMEPA1	
MATN3	CYP1A1	CACNG4	LARS2	
TSPAN2	PID1	TRIM5	CACYBP	
RPS27L	KCNK1	HS1BP3	BLMH	
COL6A2	RRH	LNX1	CD274	
FA2H	JUP	ABCC4	POLR3G	
FSTL3	CASC9	CPVL	GNAI3	
DCLK1	KCNH2	SSBP3	FAM126A	
LCN10	PCSK5	CEBPA	FOXM1	
SLC43A1	SRRM4	TTC23L	ADCY10P1	
DQX1	C9orf106	KLK14	AGPAT5	
ADGRL3	FLRT3	USH1G	SAP18	
ACACA	VIPR1	ASCL2	PGAM5	
NYNRIN	TRIM31	PEX12	PLAGL1	
OXCT1	TSPOAP1	FKBP6	MET	
ANKRD2	KCNK5	ACCS	LRRC8D	
GPRIN2	MYO6	CD200	HEBP2	
ODF3L2	COL3A1	GJC2	SELENOW	
HHIPL1	TNFSF15	SLC35F3	UBE2F	
SLC38A4	RNF224	CPE	KIAA1217	
CAMK1	NPM2	ABCC3	C20orf141	
PPM1K	INTS6L	EHD2	MAT1A	
SCG2	BTC	LAS1L	ANKRD11	
CLYBL	SBSN	DCDC1	BARX2	
GPR153	SLAMF7	PSG1	LAMA3	
SCN9A	SOX2	CFAP43	C11orf1	
MYEOV	SH2D3A	CD70	CMSS1	
ETNK2	IL12RB1	RILP	MMEL1	
TPK1	FCGBP	RGS11	DDX39A	
CDHR3	PLCH1	PDGFA		3-Mar
DUSP9	SH3TC2	PTPRH	PKD1L1	
FGF14	SPIN4	CDSN	KRTAP10-4	
FAM167B	HYDIN	TXLNA	TSPAN5	
ADM	CALHM3	HSD17B4	DHX34	
NCF1	GGT5	ZNF524	HAT1	
SLC4A3	SMCO1	FRMD4A	RRP9	
S1PR5	FN1	C9orf43	SH3BP4	
TCF21	AGAP2	AMDHD1	SUSD4	
MOXD1	C9orf152	TESMIN	EXOSC4	
AKT3	CACFD1	UPK1A	BSN	
XBP1	RAB41	TBC1D8B	THUMPD2	
VLDLR	MANBAL	ZBTB45	OTULIN	
POPDC3	ENTPD8	IQCD	TREM1	
OSR2	SMPDL3B	CGB1	TMEM41A	

NFAM1	GCHFR	SLC25A35	KRTAP1-5
ANKRD55	ISY1	NEK1	DNAJA4
BEND4	POU3F2	LENG9	CDC123
EN2	TRIM46	ESYT3	EGFL8
PLXND1	UBR5	CBLB	SPATA5L1
GPR176	WDYHV1	ABHD1	FUZ
PLEKHG4B	IFI44	PCDHB4	FAM90A1
LTC4S	CYP4F11	RAP1GAP	UBA6
SEMA4D	SLC28A1	EMP3	MCM5
TADA2A	RBM11	S100A3	SPR
SHF	IQCD	GLRX	HIST1H4L
MPC2	PLA2G4D	PRPSAP1	SLC19A1
SHANK1	TRPV3	FAAH2	B3GLCT
FBXO44	RIPK4	TMEM173	CENPC
PPM1D	FAM199X	AK8	NASP
APLP1	TOX3	ZNF821	FAM107B
SLC2A10	SLIT2	BCO1	GGCT
DUSP14	KCNJ8	SLC26A11	SOCS1
ALDH8A1	EDA	SDR42E1	CAPN7
MED12L	PRRG2	C1R	TFF2
TDO2	CRB3	CYP4F3	RNF26
NMT2	SOBP	GULP1	RNASEH1
AGPAT4	RGS7	CCDC191	FKBP14
B3GNT4	CACNA1E	MMP13	CLP1
PLEKHA2	STARD6	FLNC	PRIM2
FAM205A	SLC34A3	TP53INP2	CTPS1
RNASE2	GUCY1B2	COL4A6	MALL
ZIC2	TMCC3	TRIM34	HIST1H4I
DPYSL3	DGKA	NOS3	C6orf15
SCN3B	INA	IRX6	DNAJB1
AIG1	FIGNL1	ARMC9	IL4R
SNX22	PIP5KL1	ANKRD29	TSTA3
SLC22A1	PRSS22	FBXO24	ING3
GABRD	MPP7	PEX6	WAPL
PEAR1	ZFP36L2	NTN1	SRMS
RNF165	LAMB4	AGA	GUCD1
CYP3A7	FAM83D	ATL1	MASTL
LRRC75B	CCDC148	PDLIM1	MGAM
ANXA8	CSF1	GRTP1	MICU2
VPS37C	HPR	SIGIRR	PARPBP
CTHRC1	JDP2	FCSK	MPV17L2
GPR37	ARHGDIB	ADSSL1	RCL1
ASTN2	KLF4	DLL3	PNPT1
TRIM61	SPTB	SPTLC3	SAP30
FRG2B	SLFN13	TMEM44	KRTAP19-5
CCDC85A	MGP	IFT140	HTRA2
ZNF843	FAT2	ZBED2	CCDC134
KIF12	MYB	CALCR	OXSR1
TTL	FOXA1	SPINK2	GRIN2D
C17orf78	EXD1	CCDC22	TRAF2
DDI1	MYO5C	CTSO	WDR1
GREB1	SH3GL3	LRRC75B	CCNA2
LIPC	HSBP1L1	PGLYRP4	CHN1
CYP11A1	ZNF888	CFAP70	SLC25A16
NBPF8	GRIN3B	ADRB1	KRR1
TP53I11	SPRY2	KALRN	IL20RA
BICC1	MYLIP	DCXR	AGPS
COCH	SYTL1	COG2	A2M
NKX3-2	USP17L1	CEP120	FAM3D
CEMIP	HLA-DPA1	LRRC46	AMPD2
EFNA2	RRM2B	NRG1	TELO2
TTYH2	BRWD3	CYSTM1	CAPN6
SLC39A13	GCK	BTBD16	AEN
STEAP3	PMP2	CARF	CCNC
YWHAQ	CHCHD2	ALDH3A1	APOBEC3C
ITGAL	PRRX2	RAB33A	TCF4
TUBB4A	PDXP	RNF113B	CHST4
MRPS21	CDKN1A	VPREB3	OR4C3
HSPG2	STMN3	TNNI2	PCYT1A
VAX2	SAMD9L	CERCAM	MYEOV
ITGB8	EFNB2	SUSD3	CCNE1
PCBP3	ADGRD1	PSAT1	IMPAD1
PRDM7	PSMD10	PRSS3	TMEM108
ANPEP	SLC6A9	ZNF774	CHAF1A
CDKL2	CFAP70	MYOZ1	GCDH
METRNL	NCMAP	PSCA	ACTRT3
BOK	ACTL7A	SNAPIN	NARS2
YWHAB	AOC3	HOMER2	CCR5
ZNF439	DCST2	IFI6	LAP3
FAM43B	PRSS3	MT1E	NDUFAF4
HNF1B	LRRC66	KCNN1	IDI1
LAMA1	PODNL1	DNASE1	HGH1
CLGN	TSTD1	OTUD5	FOSL1
KANK1	NTN4	ACADS	CHRNA5
TENM2	VTCN1	RAB40B	TRIM36
FZD9	TRIM10	PTPN21	MCM4
COL18A1	SYT8	STXBP6	CYP27C1
CD7	ID1	SPA17	QSOX1
PGBD5	VSTM2L	BCL7A	WFDC2
COMMD8	MAPKAPK3	BCAS1	ZWINT
GALNT9	STS	VTCN1	TMEM217
ABCA3	SMC1B	TXNRD2	HSP90AA1

LPO
RND2
SLC39A6
SYBU
CLDN14
SLC26A5
PDIA2
SLC26A9
SH2B3
KHK
OPLAH
C19orf38
FTCDNL1
SLC2A6
RSAD1
ACSL1
FHIT
SLC35E4
EPHA4
MVB12B
ADAMTSL2
RAB9A
TRPC3
PPFIBP2
POLR2J
COQ10B
CRB2
GRM6
FGF8
ZNF114
CENPX
SMTN
BLVRA
ADAMTS18
PDZK1IP1
FGL1
LIPI
NPY4R2
STAT5A
ABLIM2
RNF128
FAM184A
MYOM2
FAHD2B
SLC34A1
EIF4E3
ADGRF5
C2CD4C
TGFBRAP1
ADGRG5
GFI1B
NEXN
C10orf25
DBN1
TRIM24
C2orf27A
SLC25A18
CDK6
SLC25A30
APPBP2
PRKX
ZFHX2
FAM171A2
ARNT2
APBA2
ZNF732
IDH2
MN1
TAS1R1
ECHDC3
C10orf90
COL27A1
BEST3
SLC23A1
RCAN1
PLCB4
VTN
MACROD2
AKR1B10
VCAN
ZBTB46
ISL1
PPP1R9A
NXN
FNDC8
DNAJB5
ARFGEF3
TTC7B
MYRF
ACE2
BEGAIN
KCNIP3
PTGS1
TH
SLC39A11
BBOX1
CCDC196
HFM1
PCDH11Y
HTR6
ZDHHC11B
CNOT6L
C9orf153
SLCO1A2
ARPC5
ANKS4B
DEPTOR
SLC16A9
NLGN3
RIPK3
SMIM24
TMEM45B
ERVK-28
IMPG1
TTLL10
LAMC2
SORBS1
PLA2G2C
KIF4A
FRK
LAMA2
FAM122B
ACSM6
IL13RA1
LMTK3
C1orf105
GIMAP2
PCSK9
ANKDD1B
KPNA7
C1orf61
ASPN
SH3BGRL2
UBE2W
HSD11B2
SMIM22
ERVMER34-1
ZCCHC2
DBP
CHMP4C
MME
KLHDC9
TRNP1
DENND1C
C19orf81
SERPINA3
AWAT2
NEUROD1
DSC2
PKP3
BIK
SUSD2
COL4A6
ITGA10
NRK
TBC1D8
GBP4
PSPH
PLEKHG1
HP
PGK1
BCAS1
SERINC5
GRB14
IL22RA1
SPATA6L
TRIM15
FLRT2
RNF183
KLF5
PGLYRP1
ACADL
TEKT5
KCNC3
KRT19
GFRA1
RGS6
ZNF75D
SMIM10L2A
GRTP1
SORL1
RGS16
FIBCD1
DNAH12
FAHD2A
FAM50A
ZNF669
TRIM52
FLRT3
CHST10
SIRT3
SGSH
S100A14
PSORS1C1
AP4S1
GNG11
HIST3H2BB
DYRK3
FAM131A
TUBGCP3
STARD10
CFL2
TMEM67
IGSF22
USP43
STX2
OPHN1
CCDC57
ZNF674-AS1
ZNF132
GJB4
C1orf216
ACPP
NDRG4
ZNF572
SPINK4
BTG2
ZSCAN21
RAPH1
LYST
DNHD1
HAPLN3
ASGR1
MGLL
S100A1
DEPTOR
MLC1
RGS5
FXYD4
AS3MT
MFSD13A
PRMT2
CCDC151
POMT1
CCDC103
ACTA2
FAM20C
GNAZ
SLC2A9
ZSCAN16
SYT1
MLLT3
AQP11
TMOD1
CDK14
ALDH6A1
PLCD3
WNT6
ACOT13
SELENBP1
ATP2B4
HEXD
TLR1
KLHL41
ANK3
CUX1
SMYD3
TSEN15
C20orf96
C1QTNF3
ALAD
IDH1
MYPN
APH1B
EMC9
ECHDC2
ARMC12
FAP
ADGRB1
FAM210B
NHLRC1
COL4A5
NUAK2
ZNF549
PCBP3
RADX
ITGB1BP1
SUCLA2
PERP
RHPN2
XXYLT1
BAG2
RFC5
TACC3
IPO5
LIG1
MAP3K20
C11orf24
METTL8
PINX1
TPRKB
TCN1
ZNF114
SIPA1L1
POLE4
PRELID1
TMEM30A
CDADC1
EXT1
THBS1
ZNF560
TNIP1
RASSF8
TFCP2L1
MCEE
RAD51
RASL10A
GEMIN5
POLR1B
SUB1
NUDCD1
TRIM24
SLC26A9
NDC80
NUP35
LTV1
RECQL4
AFG3L2
NIT2
GRWD1
POP1
SLC12A7
C8G
SLC39A8
ANAPC4
CCN1
BMPR1A
B3GNT7
ZFP36L2
BCLAF1
JRKL
EXOSC9
OSBPL3
MAP4K4
TMEM97
NFKBIZ
CISD1
GDNF
GLMN
TFAM
TRAF3
ABLIM2
PRKRA
GEMIN4
TRAIP
AUNIP
HPRT1
SLC25A46
CDCA5
GABBR2
SIMC1
DCBLD2
CSGALNACT1
NFKBID
EDEM3
CLTB
DNAJC19
NR2C2AP
ALG3
PUS7
MAP3K9
S100A7
DDX49
HIST1H4D
ANO1
MED4
SORT1
DUSP2

RGS9	STON2	IZUMO1	GJC1
SLC35G2	CD274	ENTPD2	MCAT
PCGF2	FRMPD3	MMP24	EIF5
PRKAR2B	SLC6A11	GSR	DPH2
RBKS	ACVRL1	C15orf40	HIST1H4F
NMB	NLRP9	HLA-DMA	DYNC1LI1
TRAF5	CYP4F2	VPS35L	CDC45
NUAK1	TRMT2B	SNTA1	HSF2
DAGLA	EFNB1	SP110	SLC32A1
DOCK4	SIGLEC15	EPB41L1	DPH3
ST3GAL6	UPF3B	TTBK2	OTUB2
CMPK1	LGALS4	NEBL	NOC2L
GABBR1	LAT2	RIBC2	ACKR3
COL6A1	CCDC194	ALDH3B1	PCM1
TFEB	PLEKHG6	PLXND1	ABHD5
SLC30A4	KCNH7	CXXC5	P2RX5
BRSK1	ANKRD24	ANKFN1	FSD1
ITGA2B	COL28A1	GFOD1	CNIH2
RELN	NOXRED1	AP1G2	DHX37
ASPHD1	GRHL1	ALOX15	SSR3
MMP11	RAB27B	MAFG	DCAF16
PPARG	PLXNA2	KLK5	MFSD14A
SPEG	PYM1	FIBCD1	C12orf4
AKAP7	CDKN2AIP	UGDH	SLC5A6
PIM1	SPATA17	ACAD11	LIF
RPL29	KSR2	LAT2	TMEM35A
PAM	FBP1	VMAC	EEF1AKMT1
NRBP2	TMEM236	MORN4	PHF3
ADAM11	TMEM255A	FAM167A	DEPDC1
FADS3	ZPLD1	FAM160B2	ADI1
KRTAP5-7	ALG13	HAUS4	TRPC3
ANKRD53	TEX35	SPATA7	MDN1
NBEA	THOC2	CCL17	SPC25
BAG4	CKMT1B	ZNF578	SMYD5
TPST1	WDR78	CPSF4	APPL1
SCOC	LRRC19	JAML	GNA15
ANKRD33B	PRB4	METTL7A	POLA2
AMDHD1	ZMYND15	ZNF461	JPH1
ID3	PIWIL2	CAMK2B	TMEM14A
FAM25A	APOLD1	TNFAIP8	NGB
ZNF566	STAT4	ZNF579	ATOX1
IGF1R	MARVELD2	NME3	THNSL1
CPNE4	C10orf95	USP40	PSMA5
CIB2	BTBD19	ANG	BTG3
PHLPP2	PAK3	MICAL1	RAD54L
EPOP	SEMA4B	STX8	EBNA1BP2
KRTAP4-1	AVIL	RNF31	PDS5B
PDE11A	NR4A2	ADGRG1	TCAF2
KIF13A	ARHGEF9	ZKSCAN4	PROSER2
CRYL1	SYNPO2	IL21R	PPIA
CCDC192	WFDC1	REEP2	ATG5
ST3GAL5	MYH15	GAMT	EED
GHDC	FMN1	ISG20	ZADH2
AASS	PCDH12	PRODH	UTP15
RGS11	PIANP	CTF1	PDGFD
LHFPL2	CATSPERG	SH2D6	SS18L2
SOHLH2	MTMR8	UCP2	MYO6
RASSF2	FGF1	SERPINE2	PNP
RUNX2	GVQW2	FZD1	MCFD2
ADAMTS9	FAM95C	NSMCE3	CENPQ
KRBA1	ODF3	PLA2G4D	MSRA
NEO1	FAM237B	PHGDH	HSPA5
DOCK9	SMTNL1	CCDC88B	CPO
JUN	HID1	ST3GAL2	ATP13A3
MAPK11	MAP2K4	CCDC113	CEP76
PHOX2A	SFTPB	TRIM45	ACP1
GATA6	RAB7B	HINT2	KPNA3
WWC1	ICA1L	NINJ1	CYB5R2
SLC38A3	TAOK3	NRTN	DCAF1
INSR	PHACTR3	DHDH	SSU72
GK	OPRM1	HRG	ORC6
ARID5B	NCAN	MYADML2	CLPTM1L
RAPGEF1	ZNF730	TTC5	FOXRED1
CORO6	CIDEC	ANKRD1	LAYN
SYNC	CAB39	ID4	PDGFB
RRAS2	GSTO2	HOXA10	POLR3K
FTL	CLEC18A	CLIP2	TCP1
ADAMTS15	C1QTNF7	KITLG	SLC35C1
PLXNA3	HNF1A	DDIT4	POLE
GNAZ	CNTNAP3B	PTH2	RTKN
ESAM	GALNTL6	ARHGAP33	CHCHD3
ANKRD54	ADRA1D	GPR171	CDC42EP1
SLC5A6	PHLDA2	METRNL	METTL21A
PDLIM4	OAS1	FAM49A	EPB41L4A
STXBP6	ELOC	TEPSIN	TMEM52
GPSM1	ZNF280A	ATP6V0D2	PID1
SLC46A2	SLC25A5	TM7SF2	TIMM8B
TPM1	ADH6	PEPD	GLO1
SNTB1	TERF1	GAB1	SIGMAR1
TTC28	SERPINB1	GATA4	CNOT7
LATS2	FAM71A	SOD3	UBE2E1
LEPR	ACOT4	DOK4	GJB3

ABCA13	CLEC18C	ALDH5A1
PPP1R3E	ASPG	TMEM107
CSF2RA	TIAM1	ATP2C2
FBLIM1	ACTBL2	NQO1
FAXC	ELOVL4	FNDC11
KDM1B	RASIP1	LENG1
ULK2	AQP7	B3GNT9
DNM1	CASP10	TTYH3
MYEF2	DNAH7	KCNK13
C15orf54	ZNF449	MAVS
FAM155A	FAM120C	HAGHL
KDELR3	CA11	IPP
PHYH	RINL	MAP4K2
DYNC1I1	TMPO	CEACAM1
PLD1	TGM4	PKIG
PTP4A3	WNK4	DNMT3B
NACAD	FERMT3	CAP2
NFE2L3	DCP2	PROC
P3H2	MTHFD2L	GLMP
PALM	HAPLN2	USP11
PPM1M	KRT16	DNAAF1
GTF3C4	CCDC39	SNAP47
ST3GAL1	OASL	CDYL
HACD1	SLBP	CEP170
ACTR3B	GUCA1B	CTSH
ADA2	USHBP1	NAP1L5
SEC14L4	FBLN2	OPN3
PPP3CC	APOBEC2	LAMA2
TBC1D9	NR1I2	FMNL1
LIF	FAM186A	ACRBP
ME1	PTCH2	MOSPD1
MLLT6	ENOX2	MYLIP
DISC1	ACOT11	LHX6
FBXW10	NFE4	COL5A2
GUCY1A2	SESN2	SKAP1
GIPR	BTBD11	RIC8A
LDHD	RBM41	LGALS3
RNF19A	NPSR1	RAB29
HEYL	ATP6V1C1	C1RL
ITPR1	CENPP	HOXC8
SOGA3	ANAPC11	HOXC9
GDA	TCEAL1	ULK2
TMOD1	ASB4	NAMPT
SH2D3C	PDZD9	CCDC120
SCCPDH	ABCB4	DDB2
CCDC27	SLC35C1	DUS1L
ITGA11	GIPC3	CGN
PARP3	CALB2	CTSB
CPNE7	MAP1LC3C	FOXQ1
IRGQ	SUMF2	TACC2
GDF7	FDX1	HSPB1
SDR42E1	CCDC62	RPGRIP1
CASZ1	RAB31	R3HDM2
AZIN2	FAM174B	CMBL
SLC3A2	RTL3	ZDHHC11
PLA2G4A	XIAP	SEL1L3
TMEM150C	MEF2C	KIZ
TM6SF2	BMP2	HERPUD1
SEMA6C	MYL2	SLC16A5
ITGAX	MKRN2OS	SSBP2
WNT4	CXorf56	KCTD18
GNA12	ABCA7	BATF2
EFCAB12	TMEM205	DUSP12
MYO7B	BCL2A1	NEIL1
ZNF25	TMTC2	OSCP1
C1QTNF3	SLC6A16	AKNAD1
LAT	ITGB2	SOX15
MAPRE3	VSIG10	GCHFR
HCFC1R1	CEACAM1	CLYBL
PPME1	KPNA5	CPNE2
KIF25	NAT1	ZNF358
PPP2R3A	ARFGEF1	C19orf54
F12	POC1B-GALNT4	C1orf116
CBLB	CXCL16	ZNF573
TRIO	CAAP1	NKX2-8
ADAMTS10	EFCAB5	GRIPAP1
COL4A2-AS2	CNGA1	WDR44
NID1	PYGM	GPS1
TLE1	MISP	DIP2A
HAUS8	ARGLU1	BAIAP2
NF2	MYO1G	MSN
TMOD2	MBP	ZMYM6
PSPN	TAF1	ZNF777
KIFC3	UBALD2	CAT
FZD2	USP6NL	CFAP53
CDR1	VEGFC	NUDT18
SQSTM1	IL12RB2	PPL
CNPPD1	C6orf223	STXBP1
STRADB	XKR5	ACTR1B
IRS2	TMEM238	BORCS6
TREH	SLC25A45	RHCG
DENND5A	OGT	GABRE

RASL11B
LYSMD1
PXDC1
VPS26B
CELF5
PRSS12
CYLD
SLC17A1
RAB13
TLL1

ZNF324B
CSF1R
POU6F1
SYT1
LSM11
C5
ZP3
MCTS1
CAMK1G
ATG4A
NKG7
CISH
ELF4
HSPA1B
CSTF2
CORO1A
LLGL2
ASF1B
MYO18A
PUDP
FAM135A
LAMB3
TPCN1
CCDC102B
PPFIA4
MBOAT2
AZIN1
HAL
ABHD17C
TLE6
CACNG6
PAX9
DDR1
SYTL2
ALS2CL
SPOCK2
LAS1L
NXT2
OAS3
PTK6
NKAP
FAM104B
C3orf20
CSPP1
PRSS16
CHRNA9
SPEF1
TOM1L2
PHKG1
CHSY3
NOTCH3
LRRC34
SLC6A14
CD163
CD9
ANGPT4
KIF9
SYT12
DAPK2
ZNF720
DNAH11
CD70
CAPS
IL18
CSGALNACT1
NAT2
DRGX
RTTN
ANKRD18A
ALDH1L2
ASPH
LMO7
DOCK3
VWA5B2
CASP7
SPIN2A
USP43
CFAP43
PRR18
ELK3
GSDMB
RAD9B
LYNX1
HS3ST1
ZNF532
TCP11L2
PIFO
PPIL6
VSIG1
COX19
WIPF2
PER2

ITLN2
SH3BGRL2
JAK3
BNIP3
FBXO8
ADAP1
ZBTB16
MTHFR
SLC35A1
EPS8L1
PITPNM3
ARL2BP
HDAC6
IL17RC
CNTRL
ACTL7B
RBKS
RYR1
ZKSCAN3
ZNF425
RAB4A
ADAM11
FIZ1
SLURP1
ATXN1
PTHLH
GLB1L
CUL9
PLPP1
PHACTR3
SYNGR3
TOGARAM1
NT5C2
ZNF419
ASIC1
PECR
ANKH
NTF4
CA9
MRAS
CACNG6
VEGFC
ZNF324
ZNF350
ADPRM
CES2
WDR19
GLIS2
RNF122
ADCY9
SPACA9
SPICE1
TIMM9
XRN1
BBS2
ELMOD3
PPM1K
WARS
MAPK8IP3
FBXO4
IFIT2
GCNA
MATN2
TMEM8A
OGFOD3
THBS2
KMT5C
CDH24
ARHGAP45
TP53
LYG2
SSH3
TIGD2
ZMYND10
SIRT2
ST7L
ZNF280D
HPSE
FGFR1
KLHDC2
CCDC92
ABCB7
ECH1
LAMA5
MAP2K6
SEPTIN9
MYO1F
NOXA1
ZNF784
SARM1
ZNF416
ZNF169

AREG
PMEL
GPA33
ABCG2
SLC44A2
ZFAT
TGM5
ZNF584
ALAD
RNF113A
LOXL4
FAM122C
TMC8
ADAT2
EYA4
ABCB9
FHL1
NBDY
NPIPB15
DHX36
STAC
PHKA1
STAG2
TMEFF2
LETMD1
CYP7B1
SLAIN1
CYP2S1
GOT2
ALDH1A3
PLS3
USP25
TSPAN6
YIPF6
PLAUR
FAM110C
DGKI
CFAP53
ITGB1BP2
HIP1R
SLC44A1
ABRA
ANK3
ENC1
DMBX1
UCP3
ATP2B2
NUDT1
PRDM8
RIMBP3
PLLP
RNF222
SAT1
C12orf45
ANGPTL1
COL24A1
CENPI
SYTL4
ZDHHC9
NGEF
LRRC69
TLE3
ITGA7
TGFBI
NIPSNAP2
TRIM2
MANSC1
SEPSECS
CLEC20A
LDLRAP1
ABCB7
PRB3
ATF3
APOL1
SHANK2
FHAD1
PRRG4
DOCK11
ADCY7
DNAAF3
CXCR3
COL4A5
TMEM145
C10orf55
SYT7
INAVA
ZFAND1
TTLL11
WDR86
NTN1
RBP3
ZBTB4

C5orf46
AKR1C3
RNF39
RGS14
PON3
SMIM19
AMIGO2
UCN
RBL2
ZNF787
HDGFL3
DNAL4
C17orf75
CCL22
TXNIP
THG1L
CHMP2A
NLRP2
STPG1
SLC43A2
SNRNP48
TMEM135
SLC16A14
SARAF
ACSF2
TEAD2
ACSS2
RFX2
MMP15
TMEM125
TOB1
ATP6AP1
GAD1
BCAM
UEVLD
SMPD1
BACE1
SYNGR1
LARP6
AMBP
PPP1R3D
SEMA4A
MOB3C
CRABP2
ZNF239
PRAM1
H6PD
FURIN
CREB3L3
MYOF
SLC35D2
44993
MLLT11
PEX13
SOX12
RAPGEF3
PTK6
BEST4
COL9A2
BET1
KLF8
OSGEPL1
SHROOM1
ETNK2
CSNK2A2
NDUFB11
MT1A
BDNF
ENTPD1
HIBADH
IQCE
GOLT1A
NPLOC4
TRIM21
RTN4R
PPP1R35
PLSCR3
AZIN2
CLIC4
ARID3A
KRBOX4
ADARB1
SLC22A15
SLC48A1
UAP1L1
GGTLC1
TESC
RBP4
ZNF160
GSE1
MAP6D1
ADAM22

RAMP1
NPPA
CBX5
ATP9A
SEMA5A
SCYL2
MBD2
SLC4A8
TRIM59
PRICKLE3
KITLG
FUT1
DDN
DNMT3B
SNX12
TMEM40
DNAH2
LILRB5
FAM49B
LIN28A
ANGPTL4
SLCO4A1
MORF4L2
FAM166B
PSD4
IGBP1
PDE9A
P2RX2
HIST1H3C
DEFB109B
TROAP
COMMD2
TUBA1B
PLEKHG3
TMC7
SERPINB7
ETNK1
C2CD4A
CRIP2
GPR19
DDX60
SAC3D1
LAMA3
STEAP1
CNRIP1
RTBDN
IL1RAP
IL20RB
HTATSF1
P2RY4
SMCO2
TTC9
CPXM1
ATP23
UCHL1
HLA-DQA1
SKAP2
LRRC1
MECOM
MUC1
COX7B
CIT
POU5F1B
OVGP1
FIZ1
KCNK7
SETD6
FAM72A
HCFC2
MAGT1
GAS2L3
SMAD9
PLAU
TBK1
SERTAD3
MTFR1
GAB3
ACTA2
LRRC4B
REST
ECI2
ESRP1
DBP
LY6E
TTC9B
DNAJC18
TRIM54
RASGRP1
VEPH1
FRA10AC1
LAGE3
CAMTA2
PYGM
NECAB3
SPAG4
CREBL2
DSP
TTC12
C12orf76
MID2
CHD6
TIMP1
IGBP1
PTPN20
CDK18
ADD3
ZNF277
MRNIP
OSBPL7
PDE5A
TMEM8B
NVL
ZNF785
VPS50
PTGS2
TMEM154
GCH1
C1orf61
VPS72
DCTN6
ZCCHC3
PRICKLE4
STK32A
FTL
ARL3
ACOT8
C11orf65
ST3GAL3
PEX11G
PADI4
FUCA1
SRD5A3
ZBTB46
PQBP1
ACOT11
FAM117A
SHTN1
SNX18
FGF18
CORO6
ATE1
RNF24
CROT
FRMD5
ZNF552
SENP8
PNKD
HIF1AN
RALB
ALOX5
AHNAK
THAP8
FNBP1
SEMA3B
MAPK1IP1L
SFXN3
SLC22A18AS
EEFSEC
SLC25A10
SLC27A5
TK2
SUPT4H1
HRK
TF
DEF8
MSH5
KCNE5
TNFRSF18
JAZF1
WDR25
UTS2
TBC1D31

HKDC1
TLE2
DPYD
MAL
ETFB
RP2
GMFB
EFCAB11
RPS5
ZNF575
TNFSF12-TNFSF13
WDR13
NSUN4
ZNF347
NPR2
HHAT
AGFG2
C1orf35
TRIM68
C10orf95
ARRB1
TMEM59
HDHD3
TMEM139
HMOX1
SEM1
MAGI3
BEX3
PRRT3
S100A6
TMEM205
PXDC1
RBP7
SGSM2
TMEM260
SUCLG2
VPS16
NIPAL3
CREBRF
DOCK8
STON2
TFEB
DEAF1
WDR91
ARSA
STS
ERBB3
LENG8
GALM
AGTPBP1
STEAP2
VASN
S1PR4
PIK3CD
NPHP1
DUSP22
PRKAA2
BAMBI
LOC390638
MMS19
WNK2
INHBB
UHRF1BP1
VAV1
GTF3C3
ZNF395
POR
TAF1B
GDI1
NEDD4
PIGL
MEGF11
NDRG3
FAM120B
ELMOD2
RCOR2
RNASEL
RAB36
DHRS11
PACSIN1
PFKFB2
MFSD6
AREG
PGBD2
SUFU
SETBP1
MUTYH
P4HTM
ZNF671
GPT2
PEX11A
WHRN

ENO2
MST1
CBS
PCSK9
CGRRF1
LAMTOR2
SIPA1L2
GRK5
IL3RA
SHOX2
C18orf25
RHOG
ZNF197
HSD17B1
TDRKH
KMT2E
PPP2R3B
CCDC14
SLC35A2
C4B
RRP8
CTSD
PARP9
ZXDC
LSMEM1
ZNF555
FGGY
OSGIN2
PHF20
TLE5
UBTD1
PRB4
MBOAT7
ELP3
SPHK1
NOL3
ABRAXAS2
MNAT1
ZNF17
ASPH
SOBP
TFAP2C
NPEPL1
SLC12A3
CASP8
CHD9
ZFAND5
EIF2D
RNF41
LDLRAP1
POGLUT1
C14orf93
VILL
ARF6
ZNF302
PLEKHF2
FAM98C
MYORG
NCOR1
ZNF839
KLHL2
HOXA5
EMP2
RND3
PPOX
MACROD1
SLC25A23
UGT1A10
SPRY4
SERINC2
MED12
GDA
BAZ2A
PDE6G
TCP11L2
SDC4
CYP1B1
IFT122
CUEDC2
NXPH4
CUL7
RBBP9
FLOT1
BMF
TTC8
C16orf72
STC2
TMEM164
FBXO3
PCGF5
SNX6
CASP10

AKIP1
ZCCHC24
SEPSECS
SLC25A40
TAOK3
DENND3
TRIM32
CACNB3
GLTP
CDKN2A
TIGD6
CD38
CALCOCO1
RCBTB1
SLC5A11
ARVCF
CASTOR3
EXOSC1
TERF2
TMEM45B
NKAP
MPG
EFNA3
GNB1L
JMY
CHRNB1
TMEM256
NICN1
ZMYM3
SLC39A11
ZNF362
MPI
GMPR2
CREB3L4
PER2
CD163L1
ZNF641
GKAP1
RAB20
GPR37L1
PTPRCAP
ATP6AP2
ROPN1L
ID1
DPP9
C22orf23
NUDT21
C2CD5
MYO5A
MDM4
BCL2L1
DTX2
CHD2
SLC66A3
MAN2B1
MKNK1
DPY19L4
LEAP2
FBXW4
NAB2
B3GNT4
PMAIP1
SH2B2
DNTTIP1
KIF3C
KCTD15
AOX1
ZNF250
EAPP
DYNLRB1
ZNF217
SLC6A7
USP30
ABI2
OLFML3
TRAPPC6A
BSPRY
SLFN12
TMX4
ERCC2
44987
CTSA
VPS54
TPGS1
GABPB2
PGD
MYO18A
ROMO1
DNAJB5
COQ8A
CDKL1
JMJD7-PLA2G4B

RCOR3
ZNF219
MAIP1
RPL10L
CELF6
AP5M1
KRTAP2-4
C2orf74

Table S4 Correlations between AKR1C1 expression with clinical characteristics of advanced-stage LUAD patients

	n (%)			
	Total	High expression of AKR1C1	Low expression of AKR1C1	P
Gender				
Male	24	10 (41.67%)	14 (58.33%)	0.526
Female	36	18 (50.00%)	18 (50.00%)	
Age				
<60	29	14 (48.28%)	15 (51.72%)	0.809
≥60	31	14 (38.71%)	17 (61.29%)	
TNM stage				
IIIB + IIIC	3	1 (33.33%)	2 (66.67%)	1.000
IV	57	27 (47.37%)	30 (52.63%)	
Tumor invasion				
T1+T2	29	15 (51.72%)	14 (48.28%)	0.448
T3+T4	31	13 (41.94%)	18 (58.06%)	
Lymph node metastasis				
Negative	9	5 (55.56%)	4 (44.44%)	0.721
Positive	51	23 (45.10%)	28 (54.90%)	
EGFR mutation subtype				
Exon 19 del	37	16 (43.24%)	21 (56.76%)	0.598
Exon 21 L858R	23	12 (52.17%)	11 (47.83%)	
EGFR-TKI type				
Gefitinib	22	11 (50.00%)	11 (50.00%)	
Erlotinib	13	6 (46.15%)	7 (53.85%)	0.918
Icotinib	25	11 (44.00%)	14 (56.00%)	

Table S5 Predicted miRNAs potentially regulating AKR1C1

miRWalk	PITA	miRmap	microT	miRanDa
hsa-let-7a-5p	hsa-miR-197-3p	hsa-miR-185-5p	hsa-miR-185-5p	hsa-miR-185-5p
hsa-let-7a-2-3p	hsa-miR-145-5p	hsa-miR-338-3p	hsa-miR-338-3p	hsa-miR-338-3p
hsa-let-7b-5p	hsa-miR-185-5p	hsa-miR-324-3p	hsa-miR-4306	hsa-miR-365a-3p
hsa-let-7b-3p	hsa-miR-338-3p	hsa-miR-556-5p	hsa-miR-4644	
hsa-let-7c-5p	hsa-miR-628-5p	hsa-miR-1913	hsa-miR-135a-5p	
hsa-let-7c-3p	hsa-miR-1286	hsa-miR-4306	hsa-miR-135b-5p	
hsa-let-7d-5p		hsa-miR-3918	hsa-miR-944	
hsa-let-7d-3p		hsa-miR-4644	hsa-miR-4731-5p	
hsa-let-7e-5p		hsa-miR-506-5p		
hsa-let-7e-3p				
hsa-let-7f-1-3p				
hsa-miR-15a-3p				
hsa-miR-16-1-3p				
hsa-miR-17-5p				
hsa-miR-17-3p				
hsa-miR-18a-5p				
hsa-miR-18a-3p				
hsa-miR-19a-5p				
hsa-miR-19b-1-5p				
hsa-miR-21-3p				
hsa-miR-22-5p				
hsa-miR-22-3p				
hsa-miR-23a-5p				
hsa-miR-23a-3p				
hsa-miR-24-1-5p				
hsa-miR-24-3p				
hsa-miR-25-3p				
hsa-miR-26b-3p				
hsa-miR-27a-5p				
hsa-miR-28-3p				
hsa-miR-29a-5p				
hsa-miR-29a-3p				
hsa-miR-30a-3p				
hsa-miR-31-5p				
hsa-miR-31-3p				
hsa-miR-32-3p				
hsa-miR-92a-1-5p				
hsa-miR-92a-3p				
hsa-miR-92a-2-5p				
hsa-miR-93-5p				
hsa-miR-93-3p				
hsa-miR-96-5p				
hsa-miR-96-3p				
hsa-miR-98-5p				
hsa-miR-99a-5p				
hsa-miR-99a-3p				
hsa-miR-101-5p				
hsa-miR-29b-1-5p				

hsa-miR-29b-3p
hsa-miR-29b-2-5p
hsa-miR-103a-2-5p
hsa-miR-103a-3p
hsa-miR-103a-1-5p
hsa-miR-105-5p
hsa-miR-105-3p
hsa-miR-106a-5p
hsa-miR-107
hsa-miR-196a-5p
hsa-miR-197-5p
hsa-miR-197-3p
hsa-miR-198
hsa-miR-199a-5p
hsa-miR-199a-3p
hsa-miR-208a-5p
hsa-miR-129-5p
hsa-miR-129-1-3p
hsa-miR-148a-5p
hsa-miR-148a-3p
hsa-miR-30c-2-3p
hsa-miR-139-5p
hsa-miR-139-3p
hsa-miR-147a
hsa-miR-7-5p
hsa-miR-10b-5p
hsa-miR-10b-3p
hsa-miR-34a-5p
hsa-miR-34a-3p
hsa-miR-181b-5p
hsa-miR-181c-5p
hsa-miR-181c-3p
hsa-miR-182-5p
hsa-miR-187-3p
hsa-miR-199b-5p
hsa-miR-199b-3p
hsa-miR-204-5p
hsa-miR-205-3p
hsa-miR-210-5p
hsa-miR-210-3p
hsa-miR-211-5p
hsa-miR-211-3p
hsa-miR-212-5p
hsa-miR-212-3p
hsa-miR-214-5p
hsa-miR-214-3p
hsa-miR-215-5p
hsa-miR-215-3p
hsa-miR-216a-5p
hsa-miR-216a-3p
hsa-miR-217-5p

hsa-miR-218-5p
hsa-miR-218-1-3p
hsa-miR-218-2-3p
hsa-miR-219a-1-3p
hsa-miR-221-5p
hsa-miR-221-3p
hsa-miR-222-5p
hsa-miR-222-3p
hsa-miR-224-5p
hsa-miR-224-3p
hsa-miR-200b-5p
hsa-let-7g-5p
hsa-let-7g-3p
hsa-let-7i-5p
hsa-let-7i-3p
hsa-miR-15b-5p
hsa-miR-23b-5p
hsa-miR-23b-3p
hsa-miR-27b-5p
hsa-miR-30b-3p
hsa-miR-122-5p
hsa-miR-124-5p
hsa-miR-124-3p
hsa-miR-125b-5p
hsa-miR-125b-1-3p
hsa-miR-128-1-5p
hsa-miR-128-3p
hsa-miR-132-5p
hsa-miR-132-3p
hsa-miR-135a-3p
hsa-miR-135a-2-3p
hsa-miR-137-5p
hsa-miR-138-2-3p
hsa-miR-140-5p
hsa-miR-140-3p
hsa-miR-141-5p
hsa-miR-142-3p
hsa-miR-143-5p
hsa-miR-144-3p
hsa-miR-145-5p
hsa-miR-191-3p
hsa-miR-9-5p
hsa-miR-125a-5p
hsa-miR-125a-3p
hsa-miR-127-5p
hsa-miR-129-2-3p
hsa-miR-134-5p
hsa-miR-134-3p
hsa-miR-138-1-3p
hsa-miR-146a-3p
hsa-miR-149-5p

hsa-miR-149-3p
hsa-miR-150-3p
hsa-miR-154-5p
hsa-miR-154-3p
hsa-miR-185-5p
hsa-miR-185-3p
hsa-miR-186-3p
hsa-miR-188-5p
hsa-miR-188-3p
hsa-miR-193a-5p
hsa-miR-193a-3p
hsa-miR-194-5p
hsa-miR-195-5p
hsa-miR-195-3p
hsa-miR-206
hsa-miR-320a-5p
hsa-miR-320a-3p
hsa-miR-200c-5p
hsa-miR-200c-3p
hsa-miR-155-5p
hsa-miR-128-2-5p
hsa-miR-194-3p
hsa-miR-106b-5p
hsa-miR-106b-3p
hsa-miR-29c-5p
hsa-miR-30c-1-3p
hsa-miR-200a-5p
hsa-miR-302a-3p
hsa-miR-101-2-5p
hsa-miR-219a-2-3p
hsa-miR-34b-5p
hsa-miR-34b-3p
hsa-miR-34c-5p
hsa-miR-34c-3p
hsa-miR-299-5p
hsa-miR-299-3p
hsa-miR-301a-5p
hsa-miR-99b-5p
hsa-miR-99b-3p
hsa-miR-296-5p
hsa-miR-296-3p
hsa-miR-130b-5p
hsa-miR-361-5p
hsa-miR-361-3p
hsa-miR-362-5p
hsa-miR-363-5p
hsa-miR-302b-5p
hsa-miR-302c-5p
hsa-miR-302c-3p
hsa-miR-302d-5p
hsa-miR-302d-3p

hsa-miR-370-5p
hsa-miR-370-3p
hsa-miR-371a-5p
hsa-miR-371a-3p
hsa-miR-372-3p
hsa-miR-373-5p
hsa-miR-375-5p
hsa-miR-375-3p
hsa-miR-376a-5p
hsa-miR-377-5p
hsa-miR-378a-5p
hsa-miR-381-5p
hsa-miR-381-3p
hsa-miR-383-5p
hsa-miR-330-5p
hsa-miR-328-5p
hsa-miR-328-3p
hsa-miR-342-5p
hsa-miR-337-3p
hsa-miR-323a-5p
hsa-miR-326
hsa-miR-151a-5p
hsa-miR-135b-5p
hsa-miR-135b-3p
hsa-miR-148b-3p
hsa-miR-331-5p
hsa-miR-324-3p
hsa-miR-338-5p
hsa-miR-338-3p
hsa-miR-339-5p
hsa-miR-339-3p
hsa-miR-335-3p
hsa-miR-325
hsa-miR-345-5p
hsa-miR-345-3p
hsa-miR-346
hsa-miR-196b-3p
hsa-miR-422a
hsa-miR-423-5p
hsa-miR-423-3p
hsa-miR-424-3p
hsa-miR-425-5p
hsa-miR-425-3p
hsa-miR-18b-5p
hsa-miR-18b-3p
hsa-miR-20b-5p
hsa-miR-20b-3p
hsa-miR-448
hsa-miR-449a
hsa-miR-450a-5p
hsa-miR-431-5p

hsa-miR-431-3p
hsa-miR-433-5p
hsa-miR-329-5p
hsa-miR-329-3p
hsa-miR-452-5p
hsa-miR-452-3p
hsa-miR-409-3p
hsa-miR-412-5p
hsa-miR-412-3p
hsa-miR-483-5p
hsa-miR-483-3p
hsa-miR-484
hsa-miR-485-5p
hsa-miR-485-3p
hsa-miR-486-5p
hsa-miR-486-3p
hsa-miR-489-5p
hsa-miR-489-3p
hsa-miR-490-5p
hsa-miR-490-3p
hsa-miR-491-5p
hsa-miR-491-3p
hsa-miR-511-5p
hsa-miR-146b-5p
hsa-miR-146b-3p
hsa-miR-202-3p
hsa-miR-492
hsa-miR-432-5p
hsa-miR-432-3p
hsa-miR-494-5p
hsa-miR-494-3p
hsa-miR-495-5p
hsa-miR-496
hsa-miR-193b-5p
hsa-miR-193b-3p
hsa-miR-497-3p
hsa-miR-181d-3p
hsa-miR-512-5p
hsa-miR-512-3p
hsa-miR-498-5p
hsa-miR-520e-3p
hsa-miR-515-3p
hsa-miR-519e-3p
hsa-miR-520f-5p
hsa-miR-520f-3p
hsa-miR-519c-5p
hsa-miR-520a-5p
hsa-miR-520a-3p
hsa-miR-526b-5p
hsa-miR-526b-3p
hsa-miR-519b-5p

hsa-miR-525-5p
hsa-miR-525-3p
hsa-miR-523-5p
hsa-miR-523-3p
hsa-miR-520b-5p
hsa-miR-520b-3p
hsa-miR-518b
hsa-miR-526a-5p
hsa-miR-520c-5p
hsa-miR-520c-3p
hsa-miR-518c-5p
hsa-miR-518c-3p
hsa-miR-524-3p
hsa-miR-517-5p
hsa-miR-517a-3p
hsa-miR-519d-5p
hsa-miR-519d-3p
hsa-miR-517b-3p
hsa-miR-520g-5p
hsa-miR-520g-3p
hsa-miR-516b-3p
hsa-miR-518e-5p
hsa-miR-518e-3p
hsa-miR-518a-3p
hsa-miR-518d-5p
hsa-miR-518d-3p
hsa-miR-517c-3p
hsa-miR-522-5p
hsa-miR-522-3p
hsa-miR-519a-5p
hsa-miR-516a-3p
hsa-miR-519a-2-5p
hsa-miR-499a-5p
hsa-miR-499a-3p
hsa-miR-500a-3p
hsa-miR-501-5p
hsa-miR-501-3p
hsa-miR-502-5p
hsa-miR-503-5p
hsa-miR-503-3p
hsa-miR-504-5p
hsa-miR-504-3p
hsa-miR-505-3p
hsa-miR-513a-5p
hsa-miR-508-5p
hsa-miR-508-3p
hsa-miR-509-5p
hsa-miR-509-3p
hsa-miR-510-5p
hsa-miR-514a-5p
hsa-miR-532-5p

hsa-miR-532-3p
hsa-miR-455-5p
hsa-miR-455-3p
hsa-miR-376a-2-5p
hsa-miR-487b-5p
hsa-miR-487b-3p
hsa-miR-551a
hsa-miR-552-3p
hsa-miR-554
hsa-miR-92b-5p
hsa-miR-92b-3p
hsa-miR-556-5p
hsa-miR-557
hsa-miR-558
hsa-miR-564
hsa-miR-567
hsa-miR-568
hsa-miR-551b-5p
hsa-miR-551b-3p
hsa-miR-570-5p
hsa-miR-573
hsa-miR-574-5p
hsa-miR-574-3p
hsa-miR-579-5p
hsa-miR-580-5p
hsa-miR-581
hsa-miR-583
hsa-miR-584-5p
hsa-miR-584-3p
hsa-miR-585-5p
hsa-miR-585-3p
hsa-miR-587
hsa-miR-548b-5p
hsa-miR-548b-3p
hsa-miR-588
hsa-miR-589-5p
hsa-miR-550a-5p
hsa-miR-550a-3p
hsa-miR-593-5p
hsa-miR-593-3p
hsa-miR-595
hsa-miR-596
hsa-miR-597-3p
hsa-miR-598-3p
hsa-miR-600
hsa-miR-601
hsa-miR-602
hsa-miR-603
hsa-miR-604
hsa-miR-605-5p
hsa-miR-608

hsa-miR-609
hsa-miR-610
hsa-miR-611
hsa-miR-612
hsa-miR-614
hsa-miR-615-5p
hsa-miR-615-3p
hsa-miR-616-3p
hsa-miR-548c-5p
hsa-miR-618
hsa-miR-619-5p
hsa-miR-619-3p
hsa-miR-622
hsa-miR-623
hsa-miR-624-5p
hsa-miR-624-3p
hsa-miR-625-3p
hsa-miR-627-5p
hsa-miR-627-3p
hsa-miR-629-5p
hsa-miR-629-3p
hsa-miR-630
hsa-miR-631
hsa-miR-33b-3p
hsa-miR-632
hsa-miR-634
hsa-miR-636
hsa-miR-637
hsa-miR-638
hsa-miR-639
hsa-miR-640
hsa-miR-641
hsa-miR-642a-5p
hsa-miR-642a-3p
hsa-miR-643
hsa-miR-645
hsa-miR-646
hsa-miR-647
hsa-miR-648
hsa-miR-649
hsa-miR-650
hsa-miR-652-5p
hsa-miR-652-3p
hsa-miR-548d-5p
hsa-miR-661
hsa-miR-662
hsa-miR-663a
hsa-miR-449b-5p
hsa-miR-449b-3p
hsa-miR-654-5p
hsa-miR-654-3p

hsa-miR-655-5p
hsa-miR-656-5p
hsa-miR-549a-5p
hsa-miR-657
hsa-miR-658
hsa-miR-659-3p
hsa-miR-660-5p
hsa-miR-758-5p
hsa-miR-758-3p
hsa-miR-1264
hsa-miR-671-5p
hsa-miR-671-3p
hsa-miR-668-5p
hsa-miR-668-3p
hsa-miR-550a-3-5p
hsa-miR-767-5p
hsa-miR-1224-5p
hsa-miR-1224-3p
hsa-miR-320b
hsa-miR-320c
hsa-miR-1296-5p
hsa-miR-1296-3p
hsa-miR-1468-5p
hsa-miR-1271-5p
hsa-miR-1271-3p
hsa-miR-1301-3p
hsa-miR-454-3p
hsa-miR-1185-2-3p
hsa-miR-449c-5p
hsa-miR-449c-3p
hsa-miR-769-5p
hsa-miR-766-5p
hsa-miR-766-3p
hsa-miR-378d
hsa-miR-1185-1-3p
hsa-miR-762
hsa-miR-670-5p
hsa-miR-1298-5p
hsa-miR-1298-3p
hsa-miR-2113
hsa-miR-761
hsa-miR-764
hsa-miR-759
hsa-miR-765
hsa-miR-770-5p
hsa-miR-675-5p
hsa-miR-675-3p
hsa-miR-891a-3p
hsa-miR-892a
hsa-miR-874-5p
hsa-miR-874-3p

hsa-miR-890
hsa-miR-888-3p
hsa-miR-892b
hsa-miR-541-5p
hsa-miR-541-3p
hsa-miR-889-5p
hsa-miR-875-5p
hsa-miR-875-3p
hsa-miR-708-5p
hsa-miR-708-3p
hsa-miR-744-5p
hsa-miR-744-3p
hsa-miR-885-5p
hsa-miR-885-3p
hsa-miR-877-5p
hsa-miR-877-3p
hsa-miR-887-5p
hsa-miR-887-3p
hsa-miR-665
hsa-miR-873-5p
hsa-miR-873-3p
hsa-miR-543
hsa-miR-760
hsa-miR-301b-5p
hsa-miR-920
hsa-miR-921
hsa-miR-922
hsa-miR-924
hsa-miR-509-3-5p
hsa-miR-933
hsa-miR-934
hsa-miR-936
hsa-miR-937-5p
hsa-miR-937-3p
hsa-miR-938
hsa-miR-939-5p
hsa-miR-939-3p
hsa-miR-940
hsa-miR-941
hsa-miR-942-5p
hsa-miR-942-3p
hsa-miR-943
hsa-miR-1178-5p
hsa-miR-1178-3p
hsa-miR-1180-5p
hsa-miR-1180-3p
hsa-miR-1181
hsa-miR-1182
hsa-miR-1183
hsa-miR-1225-5p
hsa-miR-1225-3p

hsa-miR-1226-5p
hsa-miR-1226-3p
hsa-miR-1227-5p
hsa-miR-1227-3p
hsa-miR-1228-5p
hsa-miR-1228-3p
hsa-miR-1229-5p
hsa-miR-1229-3p
hsa-miR-1233-3p
hsa-miR-1234-3p
hsa-miR-1236-5p
hsa-miR-1236-3p
hsa-miR-1237-5p
hsa-miR-1237-3p
hsa-miR-1238-5p
hsa-miR-1238-3p
hsa-miR-1200
hsa-miR-1202
hsa-miR-663b
hsa-miR-1204
hsa-miR-1205
hsa-miR-1207-5p
hsa-miR-548j-5p
hsa-miR-1285-5p
hsa-miR-1286
hsa-miR-1287-3p
hsa-miR-1289
hsa-miR-1290
hsa-miR-1291
hsa-miR-548k
hsa-miR-1293
hsa-miR-1294
hsa-miR-1295a
hsa-miR-1299
hsa-miR-1303
hsa-miR-1304-3p
hsa-miR-1305
hsa-miR-1247-5p
hsa-miR-1247-3p
hsa-miR-1249-5p
hsa-miR-1249-3p
hsa-miR-1250-5p
hsa-miR-1250-3p
hsa-miR-1251-5p
hsa-miR-1251-3p
hsa-miR-1253
hsa-miR-1255a
hsa-miR-1258
hsa-miR-548g-5p
hsa-miR-1261
hsa-miR-1262

hsa-miR-1263
hsa-miR-548o-3p
hsa-miR-1266-5p
hsa-miR-1266-3p
hsa-miR-1268a
hsa-miR-1270
hsa-miR-1275
hsa-miR-548i
hsa-miR-1279
hsa-miR-1281
hsa-miR-1282
hsa-miR-1284
hsa-miR-1288-3p
hsa-miR-1292-3p
hsa-miR-1255b-5p
hsa-miR-1255b-2-3p
hsa-miR-664a-3p
hsa-miR-1306-5p
hsa-miR-1306-3p
hsa-miR-1307-5p
hsa-miR-1307-3p
hsa-miR-513b-5p
hsa-miR-513b-3p
hsa-miR-1321
hsa-miR-1322
hsa-miR-1197
hsa-miR-1324
hsa-miR-1469
hsa-miR-1470
hsa-miR-1538
hsa-miR-1539
hsa-miR-103b
hsa-miR-320d
hsa-miR-1825
hsa-miR-1827
hsa-miR-1908-3p
hsa-miR-1909-5p
hsa-miR-1910-5p
hsa-miR-1911-5p
hsa-miR-1911-3p
hsa-miR-1912-5p
hsa-miR-1912-3p
hsa-miR-1913
hsa-miR-1914-5p
hsa-miR-1915-5p
hsa-miR-1915-3p
hsa-miR-1972
hsa-miR-1976
hsa-miR-2110
hsa-miR-2114-5p
hsa-miR-2114-3p

hsa-miR-2115-5p
hsa-miR-2115-3p
hsa-miR-2116-5p
hsa-miR-2116-3p
hsa-miR-2117
hsa-miR-2276-5p
hsa-miR-2276-3p
hsa-miR-2277-5p
hsa-miR-2277-3p
hsa-miR-2681-5p
hsa-miR-2681-3p
hsa-miR-2682-5p
hsa-miR-2682-3p
hsa-miR-711
hsa-miR-718
hsa-miR-2861
hsa-miR-2909
hsa-miR-3115
hsa-miR-3117-3p
hsa-miR-3120-5p
hsa-miR-3121-5p
hsa-miR-3124-5p
hsa-miR-3124-3p
hsa-miR-3125
hsa-miR-3126-5p
hsa-miR-3126-3p
hsa-miR-3127-5p
hsa-miR-3127-3p
hsa-miR-3129-3p
hsa-miR-3130-5p
hsa-miR-3130-3p
hsa-miR-3131
hsa-miR-3132
hsa-miR-378b
hsa-miR-3135a
hsa-miR-466
hsa-miR-3136-5p
hsa-miR-3137
hsa-miR-3138
hsa-miR-3139
hsa-miR-548t-5p
hsa-miR-3141
hsa-miR-3142
hsa-miR-3144-5p
hsa-miR-3144-3p
hsa-miR-3145-3p
hsa-miR-1273c
hsa-miR-3147
hsa-miR-3148
hsa-miR-3150a-5p
hsa-miR-3150a-3p

hsa-miR-3151-5p
hsa-miR-3151-3p
hsa-miR-3152-5p
hsa-miR-3152-3p
hsa-miR-3074-3p
hsa-miR-3154
hsa-miR-3155a
hsa-miR-3156-5p
hsa-miR-3156-3p
hsa-miR-3157-5p
hsa-miR-3157-3p
hsa-miR-3158-3p
hsa-miR-3160-5p
hsa-miR-3161
hsa-miR-3162-5p
hsa-miR-3162-3p
hsa-miR-3165
hsa-miR-1260b
hsa-miR-3167
hsa-miR-3168
hsa-miR-3169
hsa-miR-3170
hsa-miR-3173-5p
hsa-miR-3173-3p
hsa-miR-1193
hsa-miR-323b-5p
hsa-miR-3174
hsa-miR-3175
hsa-miR-3176
hsa-miR-3177-5p
hsa-miR-3177-3p
hsa-miR-3178
hsa-miR-3180-5p
hsa-miR-3181
hsa-miR-3183
hsa-miR-3184-5p
hsa-miR-3184-3p
hsa-miR-3065-3p
hsa-miR-3186-5p
hsa-miR-3186-3p
hsa-miR-3187-5p
hsa-miR-3187-3p
hsa-miR-3188
hsa-miR-3189-5p
hsa-miR-3189-3p
hsa-miR-320e
hsa-miR-3190-5p
hsa-miR-3190-3p
hsa-miR-3191-5p
hsa-miR-3191-3p
hsa-miR-3192-5p

hsa-miR-3192-3p
hsa-miR-3193
hsa-miR-3194-5p
hsa-miR-3194-3p
hsa-miR-3195
hsa-miR-3196
hsa-miR-548x-5p
hsa-miR-3197
hsa-miR-3198
hsa-miR-3199
hsa-miR-3200-5p
hsa-miR-514b-5p
hsa-miR-514b-3p
hsa-miR-3202
hsa-miR-4295
hsa-miR-4296
hsa-miR-4297
hsa-miR-378c
hsa-miR-4294
hsa-miR-4298
hsa-miR-4304
hsa-miR-4302
hsa-miR-4306
hsa-miR-4308
hsa-miR-4311
hsa-miR-4312
hsa-miR-4313
hsa-miR-4315
hsa-miR-4316
hsa-miR-4314
hsa-miR-4320
hsa-miR-4322
hsa-miR-4321
hsa-miR-4323
hsa-miR-4324
hsa-miR-4256
hsa-miR-4257
hsa-miR-4258
hsa-miR-4259
hsa-miR-4260
hsa-miR-4253
hsa-miR-4254
hsa-miR-4252
hsa-miR-4325
hsa-miR-4326
hsa-miR-4327
hsa-miR-4265
hsa-miR-4266
hsa-miR-4267
hsa-miR-4268
hsa-miR-4269

hsa-miR-4263
hsa-miR-4270
hsa-miR-4271
hsa-miR-4274
hsa-miR-4281
hsa-miR-4277
hsa-miR-4279
hsa-miR-4278
hsa-miR-4280
hsa-miR-4285
hsa-miR-4284
hsa-miR-4286
hsa-miR-4287
hsa-miR-4292
hsa-miR-4289
hsa-miR-4290
hsa-miR-4329
hsa-miR-4330
hsa-miR-500b-3p
hsa-miR-4328
hsa-miR-3605-5p
hsa-miR-3605-3p
hsa-miR-3610
hsa-miR-3612
hsa-miR-3613-3p
hsa-miR-3614-5p
hsa-miR-3614-3p
hsa-miR-3615
hsa-miR-3616-3p
hsa-miR-3617-5p
hsa-miR-3617-3p
hsa-miR-3619-5p
hsa-miR-3619-3p
hsa-miR-23c
hsa-miR-3620-5p
hsa-miR-3620-3p
hsa-miR-3621
hsa-miR-3622a-5p
hsa-miR-3622a-3p
hsa-miR-3622b-5p
hsa-miR-3622b-3p
hsa-miR-3648
hsa-miR-3650
hsa-miR-3651
hsa-miR-3652
hsa-miR-3655
hsa-miR-3659
hsa-miR-3660
hsa-miR-3661
hsa-miR-3663-5p
hsa-miR-3663-3p

hsa-miR-3664-5p
hsa-miR-3664-3p
hsa-miR-3665
hsa-miR-3666
hsa-miR-3667-3p
hsa-miR-3668
hsa-miR-3670
hsa-miR-3672
hsa-miR-3675-5p
hsa-miR-3675-3p
hsa-miR-3677-5p
hsa-miR-3677-3p
hsa-miR-3679-5p
hsa-miR-3679-3p
hsa-miR-3680-5p
hsa-miR-3681-5p
hsa-miR-3681-3p
hsa-miR-3682-5p
hsa-miR-3682-3p
hsa-miR-3685
hsa-miR-3688-5p
hsa-miR-3689a-3p
hsa-miR-3690
hsa-miR-3691-5p
hsa-miR-3691-3p
hsa-miR-3692-5p
hsa-miR-3692-3p
hsa-miR-3713
hsa-miR-3714
hsa-miR-3180
hsa-miR-3907
hsa-miR-3689b-3p
hsa-miR-3908
hsa-miR-3909
hsa-miR-3911
hsa-miR-3914
hsa-miR-3916
hsa-miR-3917
hsa-miR-3918
hsa-miR-3919
hsa-miR-3150b-5p
hsa-miR-3150b-3p
hsa-miR-3920
hsa-miR-3921
hsa-miR-3922-5p
hsa-miR-3924
hsa-miR-3925-5p
hsa-miR-3925-3p
hsa-miR-3926
hsa-miR-3927-5p
hsa-miR-3927-3p

hsa-miR-676-5p
hsa-miR-676-3p
hsa-miR-3928-5p
hsa-miR-3929
hsa-miR-3934-5p
hsa-miR-3934-3p
hsa-miR-3935
hsa-miR-3936
hsa-miR-3937
hsa-miR-3938
hsa-miR-3939
hsa-miR-3940-5p
hsa-miR-3940-3p
hsa-miR-3943
hsa-miR-3944-3p
hsa-miR-374c-3p
hsa-miR-642b-5p
hsa-miR-550b-2-5p
hsa-miR-548o-5p
hsa-miR-1268b
hsa-miR-378e
hsa-miR-548ab
hsa-miR-4418
hsa-miR-378f
hsa-miR-4421
hsa-miR-4423-5p
hsa-miR-378g
hsa-miR-4425
hsa-miR-4428
hsa-miR-4429
hsa-miR-4430
hsa-miR-4433a-5p
hsa-miR-4433a-3p
hsa-miR-4435
hsa-miR-4439
hsa-miR-4440
hsa-miR-4441
hsa-miR-4443
hsa-miR-4444
hsa-miR-4445-5p
hsa-miR-4446-5p
hsa-miR-4446-3p
hsa-miR-4447
hsa-miR-4448
hsa-miR-4449
hsa-miR-548ag
hsa-miR-4450
hsa-miR-548ah-5p
hsa-miR-548ah-3p
hsa-miR-4451
hsa-miR-4452

hsa-miR-4453
hsa-miR-4454
hsa-miR-4455
hsa-miR-4456
hsa-miR-4457
hsa-miR-4458
hsa-miR-378h
hsa-miR-3135b
hsa-miR-4462
hsa-miR-548ai
hsa-miR-548aj-5p
hsa-miR-4465
hsa-miR-4466
hsa-miR-4468
hsa-miR-4469
hsa-miR-4470
hsa-miR-4472
hsa-miR-4473
hsa-miR-4474-5p
hsa-miR-4474-3p
hsa-miR-4476
hsa-miR-4477a
hsa-miR-4478
hsa-miR-3689c
hsa-miR-3689d
hsa-miR-3689f
hsa-miR-4479
hsa-miR-3155b
hsa-miR-4481
hsa-miR-4482-5p
hsa-miR-4482-3p
hsa-miR-4484
hsa-miR-4485-5p
hsa-miR-4485-3p
hsa-miR-4486
hsa-miR-4487
hsa-miR-4488
hsa-miR-4489
hsa-miR-4491
hsa-miR-4492
hsa-miR-4493
hsa-miR-4494
hsa-miR-4497
hsa-miR-4498
hsa-miR-4499
hsa-miR-4501
hsa-miR-4502
hsa-miR-4505
hsa-miR-4506
hsa-miR-2392
hsa-miR-4507

hsa-miR-4508
hsa-miR-4510
hsa-miR-4512
hsa-miR-4513
hsa-miR-4514
hsa-miR-4515
hsa-miR-4516
hsa-miR-4518
hsa-miR-4519
hsa-miR-4520-5p
hsa-miR-4520-3p
hsa-miR-4522
hsa-miR-4523
hsa-miR-4524a-5p
hsa-miR-4524a-3p
hsa-miR-4525
hsa-miR-4526
hsa-miR-4527
hsa-miR-4529-5p
hsa-miR-4530
hsa-miR-4533
hsa-miR-4534
hsa-miR-378i
hsa-miR-4535
hsa-miR-548am-5p
hsa-miR-1587
hsa-miR-4536-5p
hsa-miR-548an
hsa-miR-4537
hsa-miR-4538
hsa-miR-4539
hsa-miR-4540
hsa-miR-3960
hsa-miR-3972
hsa-miR-3975
hsa-miR-3977
hsa-miR-3978
hsa-miR-4632-5p
hsa-miR-4632-3p
hsa-miR-4633-5p
hsa-miR-4633-3p
hsa-miR-4634
hsa-miR-4635
hsa-miR-4636
hsa-miR-4638-5p
hsa-miR-4639-5p
hsa-miR-4640-5p
hsa-miR-4640-3p
hsa-miR-4641
hsa-miR-4642
hsa-miR-4643

hsa-miR-4644
hsa-miR-4646-5p
hsa-miR-4646-3p
hsa-miR-4647
hsa-miR-4648
hsa-miR-4649-5p
hsa-miR-4649-3p
hsa-miR-4650-5p
hsa-miR-4650-3p
hsa-miR-4651
hsa-miR-4652-5p
hsa-miR-4652-3p
hsa-miR-4653-5p
hsa-miR-4653-3p
hsa-miR-4655-5p
hsa-miR-4655-3p
hsa-miR-4656
hsa-miR-4657
hsa-miR-4658
hsa-miR-4659a-5p
hsa-miR-4659a-3p
hsa-miR-4660
hsa-miR-4661-5p
hsa-miR-4661-3p
hsa-miR-4662a-5p
hsa-miR-4659b-3p
hsa-miR-4663
hsa-miR-4664-5p
hsa-miR-4664-3p
hsa-miR-4665-5p
hsa-miR-4667-5p
hsa-miR-4667-3p
hsa-miR-4669
hsa-miR-4671-3p
hsa-miR-4672
hsa-miR-4673
hsa-miR-4674
hsa-miR-4675
hsa-miR-4676-5p
hsa-miR-4676-3p
hsa-miR-4677-5p
hsa-miR-4677-3p
hsa-miR-4678
hsa-miR-4680-5p
hsa-miR-4680-3p
hsa-miR-4681
hsa-miR-4682
hsa-miR-4683
hsa-miR-4684-5p
hsa-miR-4684-3p
hsa-miR-4685-5p

hsa-miR-4685-3p
hsa-miR-4686
hsa-miR-4687-5p
hsa-miR-4687-3p
hsa-miR-1343-5p
hsa-miR-1343-3p
hsa-miR-4689
hsa-miR-4690-5p
hsa-miR-4690-3p
hsa-miR-4691-5p
hsa-miR-4691-3p
hsa-miR-4692
hsa-miR-4694-5p
hsa-miR-4695-5p
hsa-miR-4695-3p
hsa-miR-4696
hsa-miR-4697-5p
hsa-miR-4697-3p
hsa-miR-4700-5p
hsa-miR-4701-5p
hsa-miR-4701-3p
hsa-miR-4704-5p
hsa-miR-4706
hsa-miR-4707-3p
hsa-miR-4708-5p
hsa-miR-4708-3p
hsa-miR-4709-3p
hsa-miR-203b-5p
hsa-miR-203b-3p
hsa-miR-4710
hsa-miR-4711-5p
hsa-miR-4711-3p
hsa-miR-4712-5p
hsa-miR-4712-3p
hsa-miR-4713-5p
hsa-miR-4713-3p
hsa-miR-4714-5p
hsa-miR-4714-3p
hsa-miR-4715-5p
hsa-miR-4715-3p
hsa-miR-4716-5p
hsa-miR-4716-3p
hsa-miR-3529-5p
hsa-miR-4717-5p
hsa-miR-4717-3p
hsa-miR-4718
hsa-miR-4721
hsa-miR-4722-5p
hsa-miR-4722-3p
hsa-miR-4723-5p
hsa-miR-4723-3p

hsa-miR-4724-5p
hsa-miR-4725-5p
hsa-miR-4725-3p
hsa-miR-4726-5p
hsa-miR-4726-3p
hsa-miR-4727-5p
hsa-miR-4727-3p
hsa-miR-4728-5p
hsa-miR-4728-3p
hsa-miR-4731-5p
hsa-miR-4731-3p
hsa-miR-4732-3p
hsa-miR-4733-3p
hsa-miR-4734
hsa-miR-4736
hsa-miR-4737
hsa-miR-3064-5p
hsa-miR-3064-3p
hsa-miR-4738-3p
hsa-miR-4739
hsa-miR-4740-5p
hsa-miR-4740-3p
hsa-miR-4741
hsa-miR-4742-5p
hsa-miR-4742-3p
hsa-miR-4743-5p
hsa-miR-4743-3p
hsa-miR-122b-5p
hsa-miR-4745-3p
hsa-miR-4746-5p
hsa-miR-4746-3p
hsa-miR-4747-5p
hsa-miR-4747-3p
hsa-miR-4748
hsa-miR-4749-5p
hsa-miR-4749-3p
hsa-miR-4750-5p
hsa-miR-4750-3p
hsa-miR-4752
hsa-miR-4753-3p
hsa-miR-371b-5p
hsa-miR-371b-3p
hsa-miR-4754
hsa-miR-4755-5p
hsa-miR-499b-5p
hsa-miR-499b-3p
hsa-miR-4756-5p
hsa-miR-4756-3p
hsa-miR-4757-5p
hsa-miR-4757-3p
hsa-miR-4758-5p

hsa-miR-4758-3p
hsa-miR-4761-5p
hsa-miR-4761-3p
hsa-miR-4762-3p
hsa-miR-4763-5p
hsa-miR-4764-5p
hsa-miR-4764-3p
hsa-miR-4767
hsa-miR-4769-5p
hsa-miR-4769-3p
hsa-miR-4772-3p
hsa-miR-4773
hsa-miR-4774-5p
hsa-miR-4774-3p
hsa-miR-4776-5p
hsa-miR-4776-3p
hsa-miR-4777-5p
hsa-miR-4778-3p
hsa-miR-4780
hsa-miR-4436b-5p
hsa-miR-4436b-3p
hsa-miR-4783-3p
hsa-miR-4784
hsa-miR-1245b-5p
hsa-miR-2467-3p
hsa-miR-4786-3p
hsa-miR-4787-5p
hsa-miR-4788
hsa-miR-4789-3p
hsa-miR-4793-5p
hsa-miR-4793-3p
hsa-miR-4794
hsa-miR-4795-3p
hsa-miR-4799-3p
hsa-miR-4800-5p
hsa-miR-4800-3p
hsa-miR-4804-5p
hsa-miR-4804-3p
hsa-miR-4999-5p
hsa-miR-5000-5p
hsa-miR-5001-5p
hsa-miR-5001-3p
hsa-miR-5002-5p
hsa-miR-5002-3p
hsa-miR-5003-3p
hsa-miR-5004-3p
hsa-miR-548ao-3p
hsa-miR-5006-5p
hsa-miR-5006-3p
hsa-miR-5007-5p
hsa-miR-5008-5p

hsa-miR-5008-3p
hsa-miR-5009-5p
hsa-miR-5010-5p
hsa-miR-5010-3p
hsa-miR-5011-5p
hsa-miR-5011-3p
hsa-miR-5047
hsa-miR-5087
hsa-miR-5088-5p
hsa-miR-5088-3p
hsa-miR-5089-3p
hsa-miR-5090
hsa-miR-5091
hsa-miR-5092
hsa-miR-5093
hsa-miR-5094
hsa-miR-5187-5p
hsa-miR-5187-3p
hsa-miR-5189-5p
hsa-miR-5189-3p
hsa-miR-5190
hsa-miR-5192
hsa-miR-5193
hsa-miR-5194
hsa-miR-5195-5p
hsa-miR-5195-3p
hsa-miR-5196-5p
hsa-miR-5196-3p
hsa-miR-5197-5p
hsa-miR-4524b-5p
hsa-miR-4524b-3p
hsa-miR-5571-5p
hsa-miR-5100
hsa-miR-5572
hsa-miR-548aq-5p
hsa-miR-548ar-5p
hsa-miR-548as-5p
hsa-miR-5579-3p
hsa-miR-664b-5p
hsa-miR-664b-3p
hsa-miR-5581-5p
hsa-miR-5581-3p
hsa-miR-548at-5p
hsa-miR-5584-5p
hsa-miR-5584-3p
hsa-miR-5585-5p
hsa-miR-5587-3p
hsa-miR-548au-5p
hsa-miR-1295b-3p
hsa-miR-5588-5p
hsa-miR-5588-3p

hsa-miR-5589-5p
hsa-miR-5589-3p
hsa-miR-5590-3p
hsa-miR-5591-5p
hsa-miR-5591-3p
hsa-miR-5682
hsa-miR-5684
hsa-miR-548ax
hsa-miR-5685
hsa-miR-5681b
hsa-miR-5689
hsa-miR-5692a
hsa-miR-4666b
hsa-miR-5693
hsa-miR-5694
hsa-miR-5696
hsa-miR-5697
hsa-miR-5698
hsa-miR-5699-5p
hsa-miR-5699-3p
hsa-miR-5704
hsa-miR-5705
hsa-miR-5706
hsa-miR-5708
hsa-miR-5739
hsa-miR-5787
hsa-miR-1199-5p
hsa-miR-1199-3p
hsa-miR-6068
hsa-miR-6069
hsa-miR-6070
hsa-miR-6071
hsa-miR-6072
hsa-miR-6073
hsa-miR-6075
hsa-miR-6076
hsa-miR-6078
hsa-miR-6079
hsa-miR-6080
hsa-miR-6081
hsa-miR-6083
hsa-miR-6084
hsa-miR-6085
hsa-miR-6086
hsa-miR-6088
hsa-miR-6089
hsa-miR-6090
hsa-miR-6124
hsa-miR-6125
hsa-miR-6126
hsa-miR-6127

hsa-miR-6129
hsa-miR-6130
hsa-miR-6131
hsa-miR-6133
hsa-miR-6134
hsa-miR-6499-5p
hsa-miR-6499-3p
hsa-miR-6500-5p
hsa-miR-6500-3p
hsa-miR-6501-5p
hsa-miR-6501-3p
hsa-miR-6502-5p
hsa-miR-6503-5p
hsa-miR-6503-3p
hsa-miR-6504-5p
hsa-miR-6504-3p
hsa-miR-6505-3p
hsa-miR-6506-3p
hsa-miR-6507-5p
hsa-miR-6507-3p
hsa-miR-6508-5p
hsa-miR-6508-3p
hsa-miR-6509-5p
hsa-miR-6509-3p
hsa-miR-6510-5p
hsa-miR-6511a-5p
hsa-miR-6511a-3p
hsa-miR-6512-3p
hsa-miR-6513-3p
hsa-miR-6514-5p
hsa-miR-6514-3p
hsa-miR-6515-5p
hsa-miR-6515-3p
hsa-miR-6715a-3p
hsa-miR-6715b-5p
hsa-miR-6715b-3p
hsa-miR-6716-5p
hsa-miR-6716-3p
hsa-miR-6717-5p
hsa-miR-6511b-5p
hsa-miR-6511b-3p
hsa-miR-6718-5p
hsa-miR-6719-3p
hsa-miR-6720-5p
hsa-miR-6720-3p
hsa-miR-6721-5p
hsa-miR-6722-3p
hsa-miR-892c-3p
hsa-miR-6726-5p
hsa-miR-6726-3p
hsa-miR-6727-3p

hsa-miR-6728-5p
hsa-miR-6728-3p
hsa-miR-6729-5p
hsa-miR-6729-3p
hsa-miR-6730-5p
hsa-miR-6730-3p
hsa-miR-6731-5p
hsa-miR-6731-3p
hsa-miR-6732-5p
hsa-miR-6732-3p
hsa-miR-6733-5p
hsa-miR-6734-5p
hsa-miR-6734-3p
hsa-miR-6735-5p
hsa-miR-6735-3p
hsa-miR-6736-5p
hsa-miR-6736-3p
hsa-miR-6737-5p
hsa-miR-6737-3p
hsa-miR-6738-5p
hsa-miR-6738-3p
hsa-miR-6739-5p
hsa-miR-6739-3p
hsa-miR-6740-5p
hsa-miR-6740-3p
hsa-miR-6741-5p
hsa-miR-6741-3p
hsa-miR-6742-5p
hsa-miR-6742-3p
hsa-miR-6743-5p
hsa-miR-6743-3p
hsa-miR-6744-5p
hsa-miR-6745
hsa-miR-6746-5p
hsa-miR-6746-3p
hsa-miR-6747-5p
hsa-miR-6747-3p
hsa-miR-6748-5p
hsa-miR-6748-3p
hsa-miR-6749-5p
hsa-miR-6749-3p
hsa-miR-6750-5p
hsa-miR-6750-3p
hsa-miR-6751-5p
hsa-miR-6751-3p
hsa-miR-6752-5p
hsa-miR-6752-3p
hsa-miR-6753-5p
hsa-miR-6753-3p
hsa-miR-6754-5p
hsa-miR-6754-3p

hsa-miR-6755-3p
hsa-miR-6756-5p
hsa-miR-6756-3p
hsa-miR-6757-5p
hsa-miR-6757-3p
hsa-miR-6758-5p
hsa-miR-6758-3p
hsa-miR-6759-5p
hsa-miR-6759-3p
hsa-miR-6760-5p
hsa-miR-6760-3p
hsa-miR-6761-5p
hsa-miR-6761-3p
hsa-miR-6762-5p
hsa-miR-6762-3p
hsa-miR-6763-5p
hsa-miR-6763-3p
hsa-miR-6764-5p
hsa-miR-6764-3p
hsa-miR-6765-5p
hsa-miR-6765-3p
hsa-miR-6766-5p
hsa-miR-6766-3p
hsa-miR-6767-3p
hsa-miR-6768-3p
hsa-miR-6769a-3p
hsa-miR-6770-5p
hsa-miR-6770-3p
hsa-miR-6771-5p
hsa-miR-6771-3p
hsa-miR-6772-5p
hsa-miR-6772-3p
hsa-miR-6773-3p
hsa-miR-6774-5p
hsa-miR-6774-3p
hsa-miR-6775-5p
hsa-miR-6775-3p
hsa-miR-6776-5p
hsa-miR-6776-3p
hsa-miR-6777-5p
hsa-miR-6777-3p
hsa-miR-6778-5p
hsa-miR-6778-3p
hsa-miR-6779-5p
hsa-miR-6779-3p
hsa-miR-6780a-5p
hsa-miR-6780a-3p
hsa-miR-6781-5p
hsa-miR-6781-3p
hsa-miR-6782-5p
hsa-miR-6782-3p

hsa-miR-6783-3p
hsa-miR-6784-5p
hsa-miR-6784-3p
hsa-miR-6785-5p
hsa-miR-6785-3p
hsa-miR-6786-5p
hsa-miR-6786-3p
hsa-miR-6787-5p
hsa-miR-6787-3p
hsa-miR-6788-5p
hsa-miR-6788-3p
hsa-miR-6789-3p
hsa-miR-6790-5p
hsa-miR-6790-3p
hsa-miR-6791-5p
hsa-miR-6791-3p
hsa-miR-6792-3p
hsa-miR-6793-5p
hsa-miR-6793-3p
hsa-miR-6794-5p
hsa-miR-6794-3p
hsa-miR-6795-5p
hsa-miR-6795-3p
hsa-miR-6796-5p
hsa-miR-6796-3p
hsa-miR-6797-5p
hsa-miR-6797-3p
hsa-miR-6798-3p
hsa-miR-6799-5p
hsa-miR-6799-3p
hsa-miR-6800-5p
hsa-miR-6800-3p
hsa-miR-6801-5p
hsa-miR-6801-3p
hsa-miR-6802-5p
hsa-miR-6802-3p
hsa-miR-6803-5p
hsa-miR-6803-3p
hsa-miR-6804-3p
hsa-miR-6805-5p
hsa-miR-6805-3p
hsa-miR-6806-5p
hsa-miR-6806-3p
hsa-miR-6807-5p
hsa-miR-6807-3p
hsa-miR-6808-5p
hsa-miR-6808-3p
hsa-miR-6809-5p
hsa-miR-6809-3p
hsa-miR-6810-5p
hsa-miR-6810-3p

hsa-miR-6811-5p
hsa-miR-6811-3p
hsa-miR-6812-5p
hsa-miR-6812-3p
hsa-miR-6813-5p
hsa-miR-6813-3p
hsa-miR-6814-5p
hsa-miR-6814-3p
hsa-miR-6815-5p
hsa-miR-6815-3p
hsa-miR-6816-5p
hsa-miR-6817-3p
hsa-miR-6818-5p
hsa-miR-6818-3p
hsa-miR-6819-5p
hsa-miR-6819-3p
hsa-miR-6820-3p
hsa-miR-6821-5p
hsa-miR-6821-3p
hsa-miR-6822-5p
hsa-miR-6822-3p
hsa-miR-6823-5p
hsa-miR-6823-3p
hsa-miR-6824-5p
hsa-miR-6825-5p
hsa-miR-6825-3p
hsa-miR-6826-5p
hsa-miR-6826-3p
hsa-miR-6827-5p
hsa-miR-6827-3p
hsa-miR-6828-5p
hsa-miR-6828-3p
hsa-miR-6829-5p
hsa-miR-6829-3p
hsa-miR-6830-5p
hsa-miR-6830-3p
hsa-miR-6831-5p
hsa-miR-6831-3p
hsa-miR-6832-5p
hsa-miR-6832-3p
hsa-miR-6833-5p
hsa-miR-6833-3p
hsa-miR-6834-5p
hsa-miR-6834-3p
hsa-miR-6835-5p
hsa-miR-6835-3p
hsa-miR-6780b-5p
hsa-miR-6780b-3p
hsa-miR-6836-5p
hsa-miR-6836-3p
hsa-miR-6837-3p

hsa-miR-6838-5p
hsa-miR-6838-3p
hsa-miR-6839-3p
hsa-miR-6840-3p
hsa-miR-6841-3p
hsa-miR-6842-5p
hsa-miR-6842-3p
hsa-miR-6843-3p
hsa-miR-6845-5p
hsa-miR-6845-3p
hsa-miR-6846-5p
hsa-miR-6846-3p
hsa-miR-6847-5p
hsa-miR-6848-5p
hsa-miR-6848-3p
hsa-miR-6849-3p
hsa-miR-6850-5p
hsa-miR-6850-3p
hsa-miR-6851-5p
hsa-miR-6851-3p
hsa-miR-6852-5p
hsa-miR-6852-3p
hsa-miR-6853-5p
hsa-miR-6854-5p
hsa-miR-6854-3p
hsa-miR-6855-5p
hsa-miR-6856-5p
hsa-miR-6856-3p
hsa-miR-6857-5p
hsa-miR-6857-3p
hsa-miR-6858-5p
hsa-miR-6858-3p
hsa-miR-6859-5p
hsa-miR-6859-3p
hsa-miR-6769b-5p
hsa-miR-6769b-3p
hsa-miR-6860
hsa-miR-6861-5p
hsa-miR-6861-3p
hsa-miR-6862-5p
hsa-miR-6862-3p
hsa-miR-6863
hsa-miR-6864-3p
hsa-miR-6865-5p
hsa-miR-6865-3p
hsa-miR-6866-3p
hsa-miR-6867-5p
hsa-miR-6867-3p
hsa-miR-6868-5p
hsa-miR-6868-3p
hsa-miR-6869-5p

hsa-miR-6869-3p
hsa-miR-6870-5p
hsa-miR-6870-3p
hsa-miR-6871-5p
hsa-miR-6871-3p
hsa-miR-6872-5p
hsa-miR-6872-3p
hsa-miR-6873-5p
hsa-miR-6873-3p
hsa-miR-6874-5p
hsa-miR-6874-3p
hsa-miR-6875-5p
hsa-miR-6875-3p
hsa-miR-6876-5p
hsa-miR-6876-3p
hsa-miR-6877-5p
hsa-miR-6877-3p
hsa-miR-6878-5p
hsa-miR-6878-3p
hsa-miR-6879-5p
hsa-miR-6879-3p
hsa-miR-6880-5p
hsa-miR-6880-3p
hsa-miR-6881-5p
hsa-miR-6881-3p
hsa-miR-6882-3p
hsa-miR-6883-5p
hsa-miR-6883-3p
hsa-miR-6884-5p
hsa-miR-6884-3p
hsa-miR-6885-5p
hsa-miR-6885-3p
hsa-miR-6886-3p
hsa-miR-6887-5p
hsa-miR-6887-3p
hsa-miR-6888-5p
hsa-miR-6888-3p
hsa-miR-6889-3p
hsa-miR-6890-5p
hsa-miR-6890-3p
hsa-miR-6891-5p
hsa-miR-6891-3p
hsa-miR-6892-5p
hsa-miR-6892-3p
hsa-miR-6893-5p
hsa-miR-6893-3p
hsa-miR-6894-5p
hsa-miR-6894-3p
hsa-miR-6895-3p
hsa-miR-7106-5p
hsa-miR-7106-3p

hsa-miR-7107-5p
hsa-miR-7107-3p
hsa-miR-7108-5p
hsa-miR-7108-3p
hsa-miR-7109-5p
hsa-miR-7109-3p
hsa-miR-7110-5p
hsa-miR-7110-3p
hsa-miR-7111-5p
hsa-miR-7111-3p
hsa-miR-7112-5p
hsa-miR-7112-3p
hsa-miR-7113-5p
hsa-miR-7113-3p
hsa-miR-7114-5p
hsa-miR-7114-3p
hsa-miR-7150
hsa-miR-7151-3p
hsa-miR-7152-5p
hsa-miR-7152-3p
hsa-miR-7154-3p
hsa-miR-7155-5p
hsa-miR-7155-3p
hsa-miR-7156-3p
hsa-miR-7157-3p
hsa-miR-7158-5p
hsa-miR-7158-3p
hsa-miR-7161-3p
hsa-miR-7159-5p
hsa-miR-7160-5p
hsa-miR-7162-5p
hsa-miR-7162-3p
hsa-miR-7702
hsa-miR-7703
hsa-miR-7704
hsa-miR-7706
hsa-miR-7843-5p
hsa-miR-7843-3p
hsa-miR-4433b-5p
hsa-miR-4433b-3p
hsa-miR-1273h-5p
hsa-miR-1273h-3p
hsa-miR-6516-5p
hsa-miR-7844-5p
hsa-miR-7845-5p
hsa-miR-7846-3p
hsa-miR-7847-3p
hsa-miR-7848-3p
hsa-miR-7849-3p
hsa-miR-7850-5p
hsa-miR-7851-3p

hsa-miR-7854-3p
hsa-miR-7974
hsa-miR-7975
hsa-miR-7976
hsa-miR-7977
hsa-miR-8052
hsa-miR-8053
hsa-miR-8055
hsa-miR-8056
hsa-miR-8057
hsa-miR-8058
hsa-miR-8060
hsa-miR-8064
hsa-miR-8070
hsa-miR-8071
hsa-miR-8072
hsa-miR-8073
hsa-miR-8074
hsa-miR-8075
hsa-miR-8077
hsa-miR-8078
hsa-miR-8080
hsa-miR-8081
hsa-miR-8082
hsa-miR-8083
hsa-miR-8085
hsa-miR-8087
hsa-miR-8088
hsa-miR-8089
hsa-miR-8485
hsa-miR-9500
hsa-miR-548bb-5p
hsa-miR-9898
hsa-miR-9899
hsa-miR-9902
hsa-miR-9903
hsa-miR-1843
hsa-miR-9986
hsa-miR-10226
hsa-miR-10392-5p
hsa-miR-10392-3p
hsa-miR-10394-5p
hsa-miR-10394-3p
hsa-miR-10395-5p
hsa-miR-10396a-3p
hsa-miR-10397-5p
hsa-miR-10398-5p
hsa-miR-10399-5p
hsa-miR-10399-3p
hsa-miR-10400-5p
hsa-miR-10400-3p

hsa-miR-10401-5p
hsa-miR-10401-3p
hsa-miR-10396b-3p
hsa-miR-10522-5p
hsa-miR-10523-5p
hsa-miR-10524-5p
hsa-miR-10526-3p
hsa-miR-11181-5p
hsa-miR-11181-3p
hsa-miR-11399
hsa-miR-11400
hsa-miR-11401
hsa-miR-3059-5p
hsa-miR-3059-3p
hsa-miR-3085-5p
hsa-miR-3085-3p
hsa-miR-6529-5p
hsa-miR-6529-3p
hsa-miR-9851-5p
hsa-miR-9851-3p
hsa-miR-12113
hsa-miR-12114
hsa-miR-12115
hsa-miR-12116
hsa-miR-12119
hsa-miR-12120
hsa-miR-12121
hsa-miR-12122
hsa-miR-12124
hsa-miR-12125
hsa-miR-12127
hsa-miR-12128
hsa-miR-12129
hsa-miR-12130
hsa-miR-12131
hsa-miR-12136

Table S6 Predicted lncRNAs potentially sponging miR-338-3p

FOXD2-AS1
LINC01963
DANCR
TMEM161B-AS1
LINC01446
LINC02641
KCNQ1OT1
NEAT1
MALAT1
OIP5-AS1
GABPB1-AS1
SLX1B-SULT1A4
SLX1A-SULT1A3
CRNDE
LINC02693
DSCAM-AS1
LINC01694
MIAT
MIRLET7BHG
XIST

Table S7 Differentially expressed lncRNAs in GSE199627 and GSE169513

GSE199627		GSE169513	
upregulated genes	downregulated genes	upregulated genes	downregulated genes
ST3GAL6-AS1	MIR194-2HG	LINC00341	LINC00221
LINC01638	LINC00678	LINC00942	LINC01980
PSG8-AS1	TCF7L1-IT1	C1QTNF1-AS1	CSAG4
LINC00595	LINC01792	KYNU	PQLC2L
LINC01914	LINC01556	LINC00862	TP73-AS1
SEMA6A-AS1	LINC00654	CATIP-AS2	LINC00470
LINC00622	LINC01559	PCOLCE-AS1	LINC02551
LINC02015	FENDRR	TCERG1L-AS1	PCSK6-AS1
LUARIS	LINC01204	GPR137B	RARA-AS1
AQP4-AS1	SOX21-AS1	LINC02560	TPM1-AS
AGAP2-AS1	TCEAL3-AS1	PABPC1L	LINC02341
LINC02105	EGFR-AS1	SLC25A21-AS1	FARP1-AS1
TMPO-AS1	CLDN10-AS1	NEK11	ZNF90
MEIS1-AS3	LINC01768	NAGPA-AS1	GRM7-AS1
LINC01880	FAM222A-AS1	HIPK1-AS1	PRDM5
LINC01238	HOXC-AS3	RDM1	LINC00869
LINC01694	LINC02259	WNT5A-AS1	GLRA3
LINC00486	LINC00698	SSBP3-AS1	NALCN-AS1
LINC02198	LINC01116	SPIN4-AS1	SH3RF3-AS1
MAGI2-AS3	FOXG1-AS1	LINC01290	LINC00857
ADORA2A-AS1	LINC01767	SPRY4-AS1	TNIK
LINC00867	LINC01213	ZSCAN4	MYH16
SH3RF3-AS1	NKX2-1-AS1	TNRC6C-AS1	SH3YL1
LINC00926	PHACTR2-AS1	AZIN1-AS1	FGD2
LINC01006	LINC01804	MDH1B	LINC02012
LINC01234	SPINT1-AS1	RASA4CP	SLC6A10PB
LINC02188	GRTP1-AS1	LINC01198	PSPC1-AS2
LINC02532	PHEX-AS1	HCG26	HCG21
PAPPA-AS1	LHFPL3-AS1	C21orf62-AS1	LINC01362
NEAT1	ARHGEF38-IT1	LIX1L-AS1	EPHA6
TDRKH-AS1	CASC8	LINC00243	FAM221A
SPANXA2-OT1	ARHGEF26-AS1	LYST	SAMD12
LINC00304	FAM83A-AS1	RHBG	NR2F1-AS1
FOXD3-AS1	SPRY4-IT1	FBXL13	MRE11A
LINC01366	LINC00623	PAXIP1-AS2	SHROOM4
SNHG10	RNF217-AS1	ZNF594	NCBP2-AS2
BACH1-IT3	LINC02288	TMC6	BDH1
LINC01594	LINC02516	PLS3-AS1	VPS9D1-AS1
OSMR-AS1	LINC01910	LIPE-AS1	FBXL19-AS1
SACS-AS1	LINC00431	C20orf194	MFHAS1
DISC1-IT1	STAU2-AS1	KCNK4-TEX40	C1QTNF9-AS1
LINC01315	CASC19	PTGR1	RNF144A-AS1
LINC02427	SAMD12-AS1	ZBED3-AS1	TPRXL
LINC02365	LINC01222	LINC00615	MIRLET7I
C1QTNF1-AS1	FAM230B	ZBTB20-AS2	LINC00623
LINC00211	PCOLCE-AS1	LINC00312	XXYLT1
LINC02274	NR2F1-AS1	SDCBP2-AS1	MEIS1-AS2
LINC00472	HAS2-AS1	NDUFA10	SNRK-AS1
SUCLG2-AS1	RAET1E	ATG9B	RUNX1-IT1
ITPK1-AS1	VIPR1-AS1	LURAP1L	CCSER1
LHFPL3-AS2	REV3L-IT1	ZNF571-AS1	NPTN-IT1
IL12A-AS1	LINC02042	TMC4	SIGLEC16
WDR11-AS1	INE1	LINC01556	BPTF
MIR137HG	LINC01803	TMEM44	DHRS2

THRB-AS1	LINC02367	C1orf145	LRRK1
LINC01715	LINC00634	STOX2	LINC02310
ZFHX4-AS1	RPS6KA2-IT1	LINC01003	MIRLET7G
LINC01588	ADGRD1-AS1	SF3B1	STARD13-AS
SVIL-AS1	NPSR1-AS1	DCP1B	LINC02464
LINC01364	WASIR2	LINC01269	PDK1
NECTIN3-AS1	AMMECR1-IT1	WDR19	ITFG2
IGFL2-AS1	LINC02204	SMARCD3	TRMU
TTLL7-IT1	LINC01337	PHKA2-AS1	APOBEC3B-AS1
LINC01341	LINC01312	TCF7L1-IT1	LINC01518
LINC01571	MIR2052HG	SPATA6L	OVCH1
SATB2-AS1	ZBTB46-AS1	ZNF32-AS2	PPIH
CCDC144NL-AS1	LINC01010	ATP1A1-AS1	PNPLA7
CHRM3-AS2	SOCS2-AS1	CRHR1-IT1	LINC01035
TGFA-IT1	LINC00461	PTPRH	C12orf42
LINC00862	LAMA5-AS1	SH3PXD2A-AS1	LY6G6E
LINC01776	ATG10-AS1	IL6R-AS1	LINC01173
LIF-AS1	DDIT4	SLC35D2	DUT
LINC00957	LANCL1-AS1	SCARNA21B	LINC02409
LINC01865	TMEM51-AS1	ZNF213-AS1	TRIO
LINC01763	HOTAIR	COPG2IT1	HEATR9
LINC01293	LINC01978	HEXDC	NUDT15
LINP1	LINC02038	ZNF205-AS1	ESP33
CATIP-AS1	IL20RB-AS1	SNAI3-AS1	UTP6
LINC00643	LINC01933	KDM5C-IT1	PAK3
LINC02275	LNCPRESS2	FUT1	ESCO2
CPEB1-AS1	CASC11	POMT1	ERICH1
PCCA-AS1	KRT7-AS	LINC02473	KDM5D
VLDLR-AS1	ABHD11-AS1	FER1L4	CSNK1G2-AS1
LINC01667	AZIN1-AS1	LINC01115	PDLIM3
FOXN3-AS2	DNAJC9-AS1	SIRPG-AS1	USP2-AS1
ARHGAP31-AS1	LINC02340	C9orf135	PDSS1
P3H2-AS1	HAGLROS	APTR	TTLL7
ST3GAL5-AS1	STXBP5-AS1	FAM234A	UBE2E1-AS1
SPATA3-AS1	ESRG	FAM159A	LINC01126
LINC00452	TM4SF1-AS1	DDIT4-AS1	LINC00852
LINC01692	LINC01714	USP27X-AS1	GLIS3-AS1
ADAMTS9-AS2	LINC00412	GUCY2EP	MUC19
FLG-AS1	LINC01215	STAG1	SPATA13
RASSF8-AS1	LINC01730	C9orf106	PRKCZ-AS1
LINC02009	DSCAS	OSGEPL1-AS1	MED4-AS1
ZNF503-AS2	LINC01395	CCDC18-AS1	SNHG14
TNFRSF14-AS1	UBR5-AS1	LINC00260	KIF15
ZMIZ1-AS1	LINC00578	LINC01731	CEP83
OBSCN-AS1	LINC01132	SNAR-G1	KLHL6-AS1
GATA6-AS1	SLC12A9-AS1	C2orf47	NPM1
TBX2-AS1	MORF4L2-AS1	OSMR-AS1	MAN2B2
LINC00475	HMGN3-AS1	RBPMS-AS1	ARAP1-AS2
EGOT	LINC01123	FBXO36-IT1	TRMT11
LINC02157	HM13-AS1	CDKL3	LCTL
LINC01687	LINC01094	SLC11A2	PROSER2-AS1
FARP1-AS1	LINC00543	HCG27	IL1RL2
KCNQ5-AS1	LINC02354	BLACAT1	UST
DOCK4-AS1	ZFAT-AS1	WBP2NL	ATP1B3-AS1
STARD13-AS	LINC00893	WNK2	ZGRF1
MIR3936HG	LINC00943	CRTC3-AS1	DISC1-IT1
NCBP2-AS1	LINC01800	SPTY2D1-AS1	USP12-AS1
PSMG3-AS1	DDIT4-AS1	LINC00412	CHEK2

PROSER2-AS1
INE2
BOLA3-AS1
LMCD1-AS1
STEAP3-AS1
EHMT2-AS1
RBPMS-AS1
NEXN-AS1
MYLK-AS2
ELFN1-AS1
LINC01881

ERICH6-AS1
LINC00637
PLS3-AS1
LINC02039
LINC01176
RASA2-IT1
LINC00649
LINC01504
LINC01166
LINC00944
LINC00240
LINC01239
LINC01138
RAI1-AS1
SPACA6P-AS
TMEM254-AS1
RDH10-AS1
DDN-AS1
PRICKLE2-AS1
LINC01285
ZNF341-AS1
GLYCTK-AS1
FTX
KLF3-AS1
MIR222HG
PITPNM2-AS1

FXR2
EFCAB6
GEMIN8
ZNF577
HLA-F-AS1
PLCD4
GAA
ZNF337-AS1
BTN3A1
ATG2B
RFPL3S
TMEM63A
ARSD-AS1
ZMYM4
LINC01151
XPO7
ROPN1
NEAT1
CD163L1
CHRM3-AS2
HMGCLL1
IFT27
NHS-AS1
EHHADH-AS1
RBM39
LINC01943
HM13-AS1
FAM27C
LINC00866
ZNF487
ESYT3
LINC00303
PPP1R13B
RHOBTB1
PYROXD2
CDK14
CCDC144NL-AS1
KIAA0125
BRWD1-IT1
RAI1-AS1
LINC01578
MTUS2-AS1
DNAJC7
NOXA1
MDH2
SNAR-F
BTAF1
SFXN3
GATAD1
PSTK
MIRLET7B
NSMCE4A
SLC5A4-AS1
MAP4K2
CGN
HCFC2
PLB1
PINK1-AS

CCDC138
EXOC3-AS1
CASK-AS1
PRKX-AS1
OPA1-AS1
CCAT2
PVRL3-AS1
SDCCAG3
RBSN
LMBR1
EML6
LINC01422
ASPHD1
TMEM51-AS1
SMCHD1
PSMC3IP
FAM155A-IT1
MALT1
SPNS3
SLCO4A1
DLGAP1-AS5
MORN1
CCDC17
GHRLOS
LINC01489
PDZD8
SLC25A26
CD101
VIPR1-AS1
TRAPPC12-AS1
FOXI1
NUBPL
HDDC2
EED
TSEN2
BACH1
CIITA
LINC02133
HMMR-AS1
SPRR2C
TSTD3
LRRC25
LINC01568
PSMD6-AS2
CNTLN
PITRM1

PRH1
LINC01509
PHF21A
CLK2
HCG8
C7orf13
PLEKHH2
MMP24-AS1
PYCARD-AS1
ARHGAP26-IT1
LINC01055
CEP164
TUBBP5
DIRC2
EXOSC1
LINC01964
HCP5
BET1
SMYD3-IT1
ZNF516
TBC1D2B
MHRT
USP24
MDGA1
LINC01251
ZNF252P
OSBPL7
LMF1
ZNF436-AS1
HLA-H
MMP17
CCSER2
SEMA4F
SKIL
FAM13A-AS1
ZNF33B
DENND3
POLE2
CDC42-IT1
SNHG20
ERC1
FAM186B
SMAD1-AS1
ZBTB46-AS1
HACD2
RSPH3
GNMT
FRRS1
ELMOD2
VPS54
HHATL-AS1
ZNF890P
FAM225A
CHKA
WDFY3-AS1
LINC00476
LINC01348
DDX39B-AS1

PLD2
RNF165
SLC25A43
ENTPD1-AS1
DTNB
SLC35E2B
BLOC1S1
TRAPPC2B
MYO16-AS1
LNCSRLR
ZNF252P-AS1
ZSWIM8-AS1
NAV2-AS3
