# Supplementary figures and images for: NEAT1_1 confers gefitinib resistance in lung adenocarcinoma through promoting AKR1C1-mediated ferroptosis defence
Source: Cell Death Discov. 2024 Mar 12;10:131. doi: 10.1038/s41420-024-01892-w (PMC10933475; doi:10.1038/s41420-024-01892-w)

Figure 2C

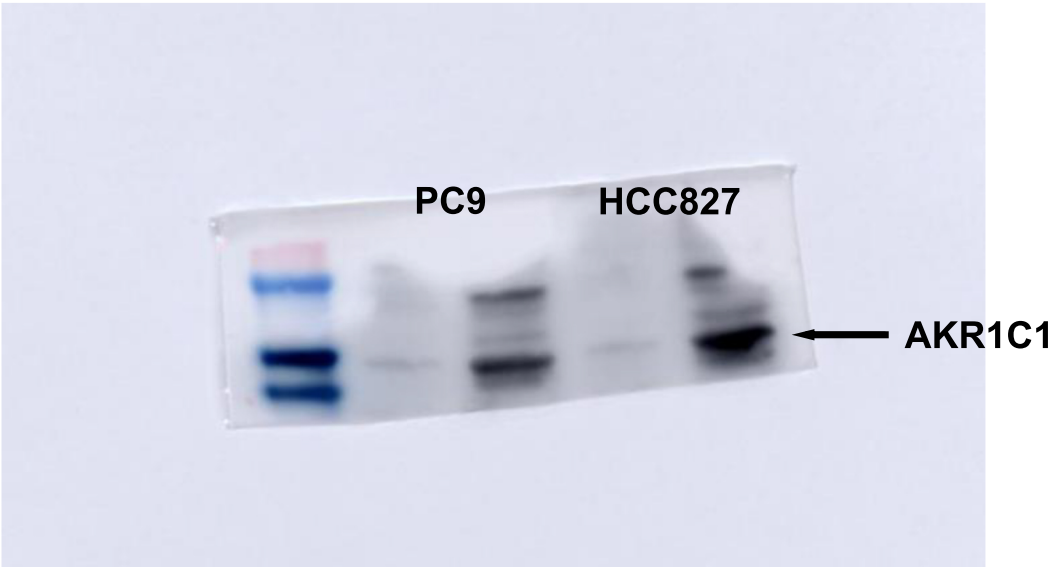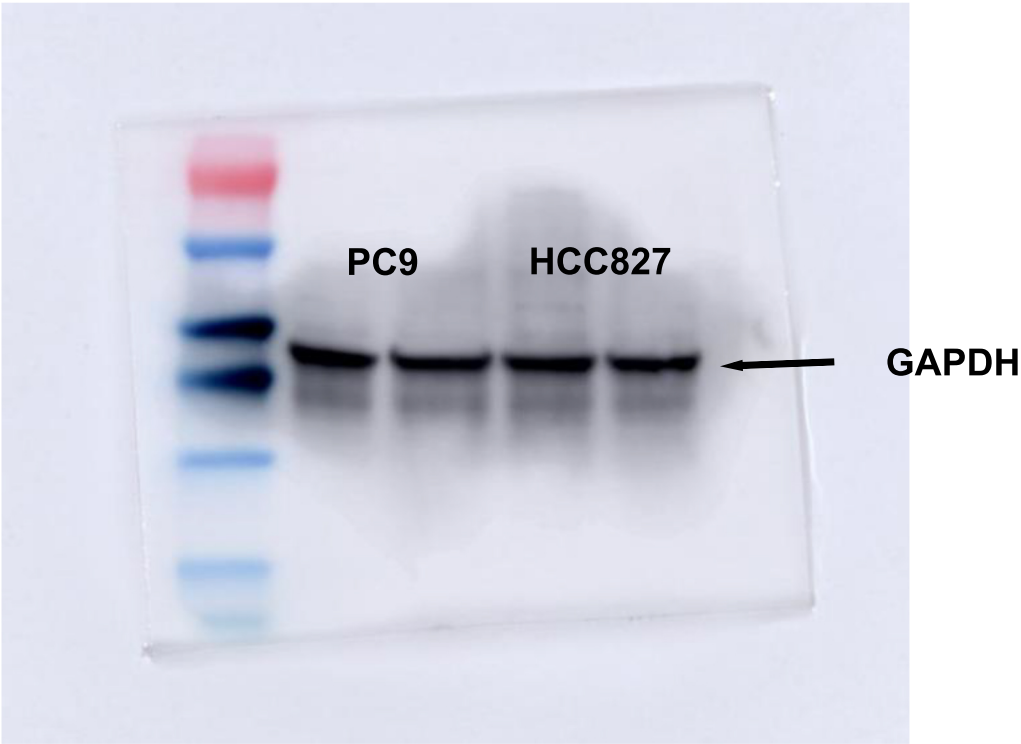

Figure 2D

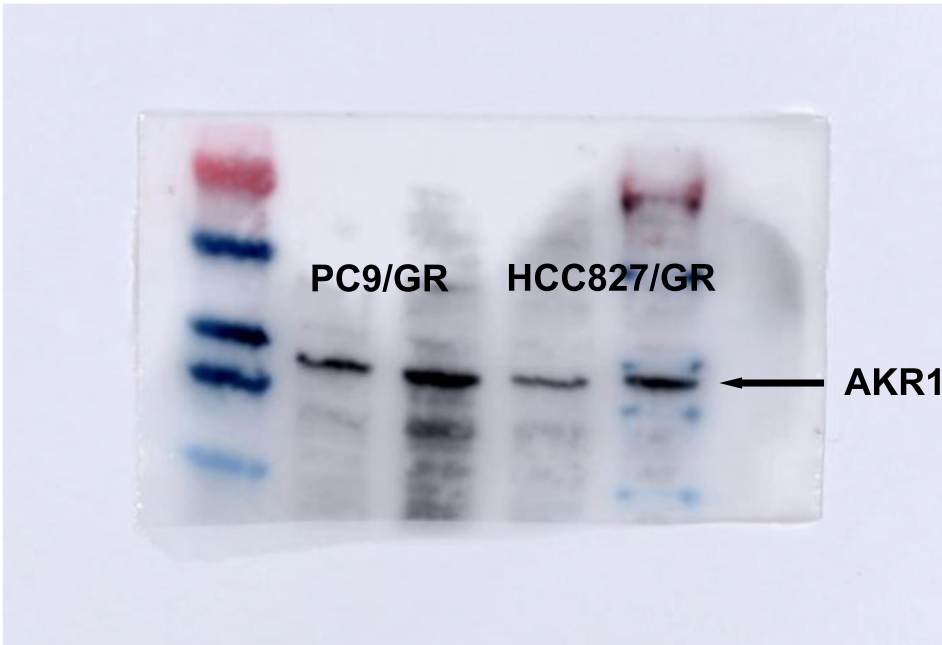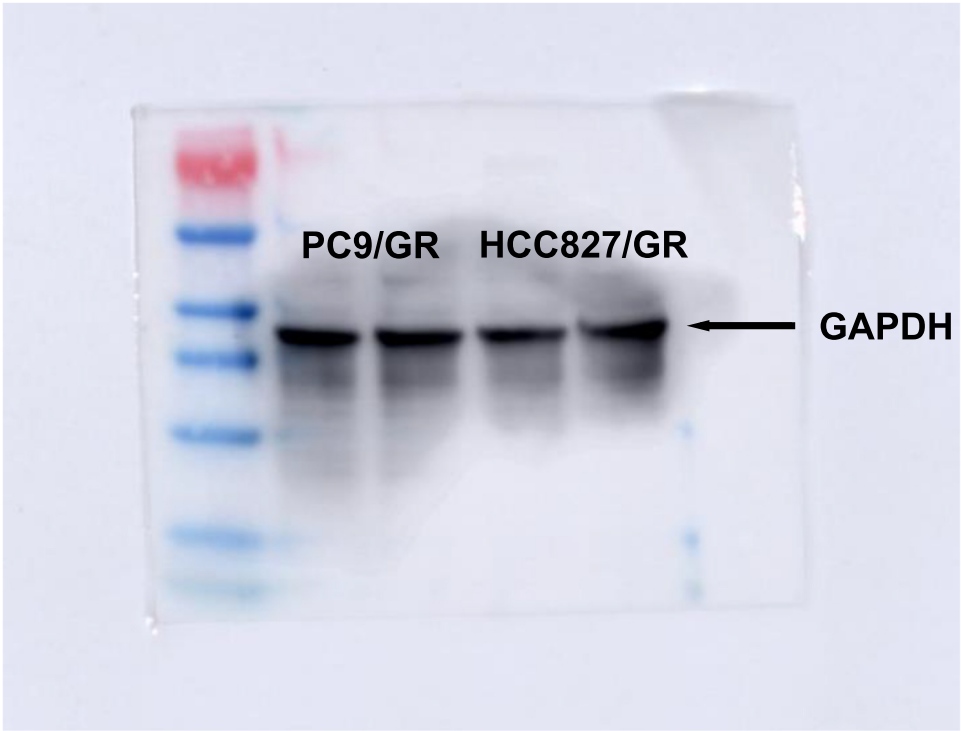

Figure 2E

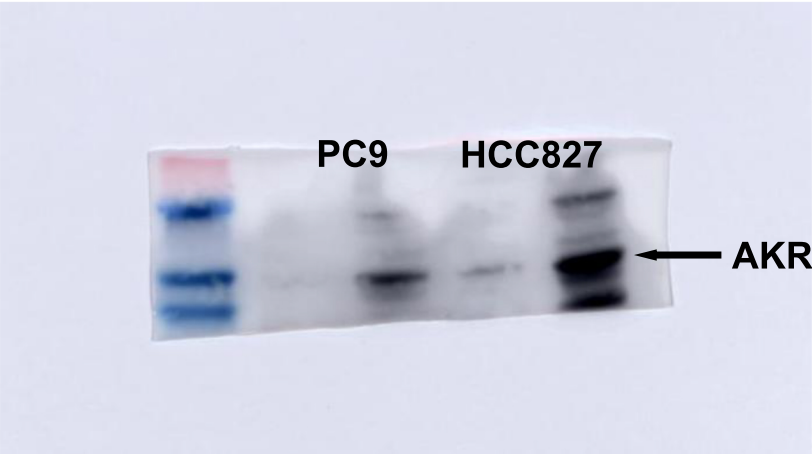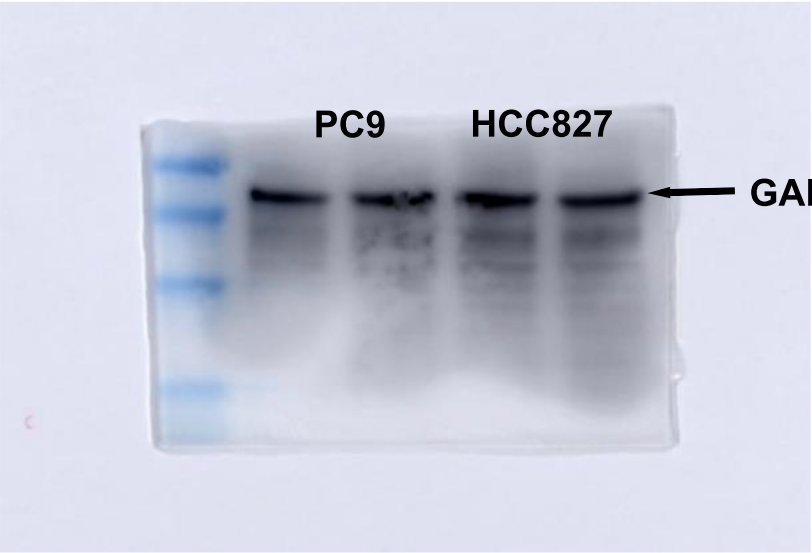

Figure 5L

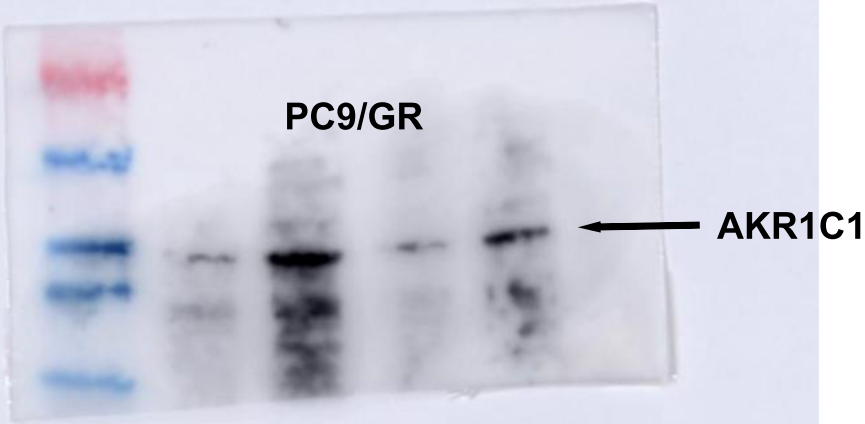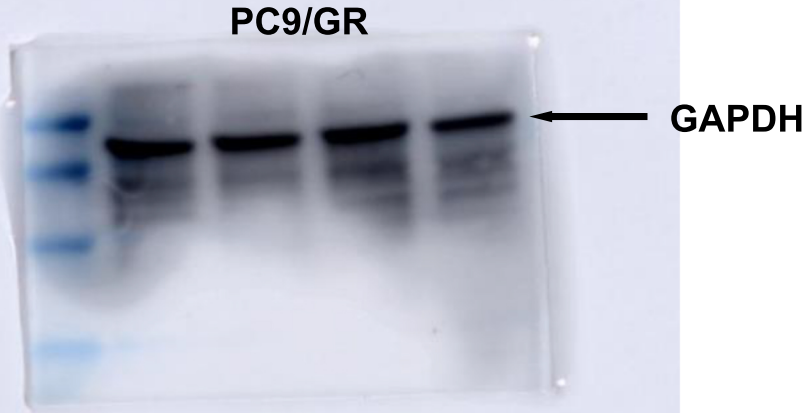

Figure 5M

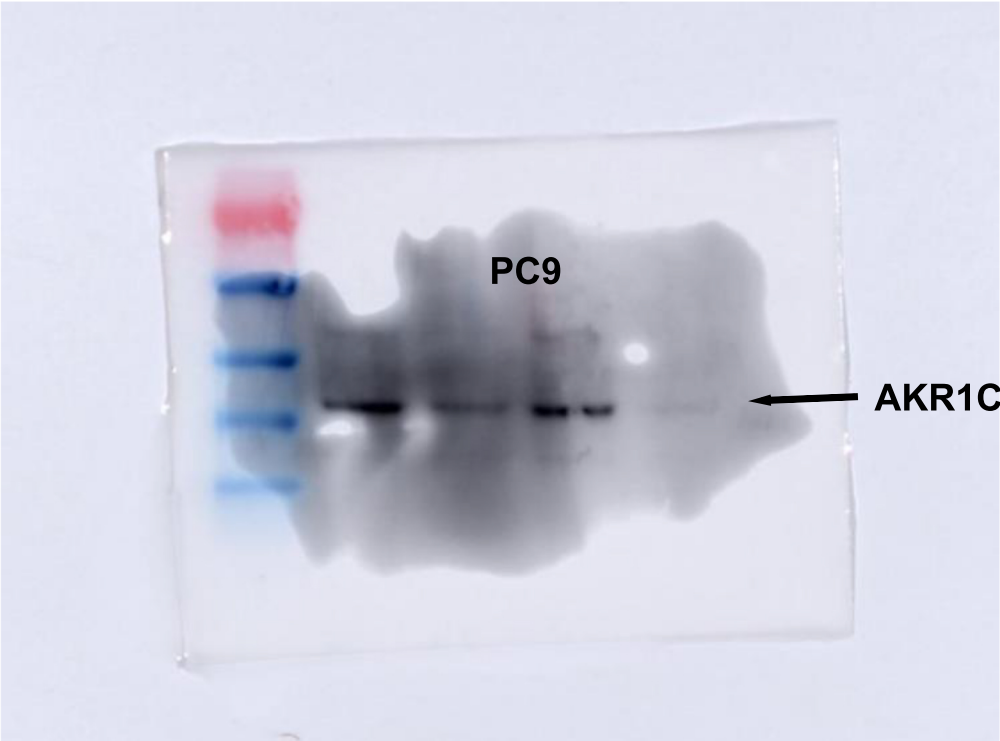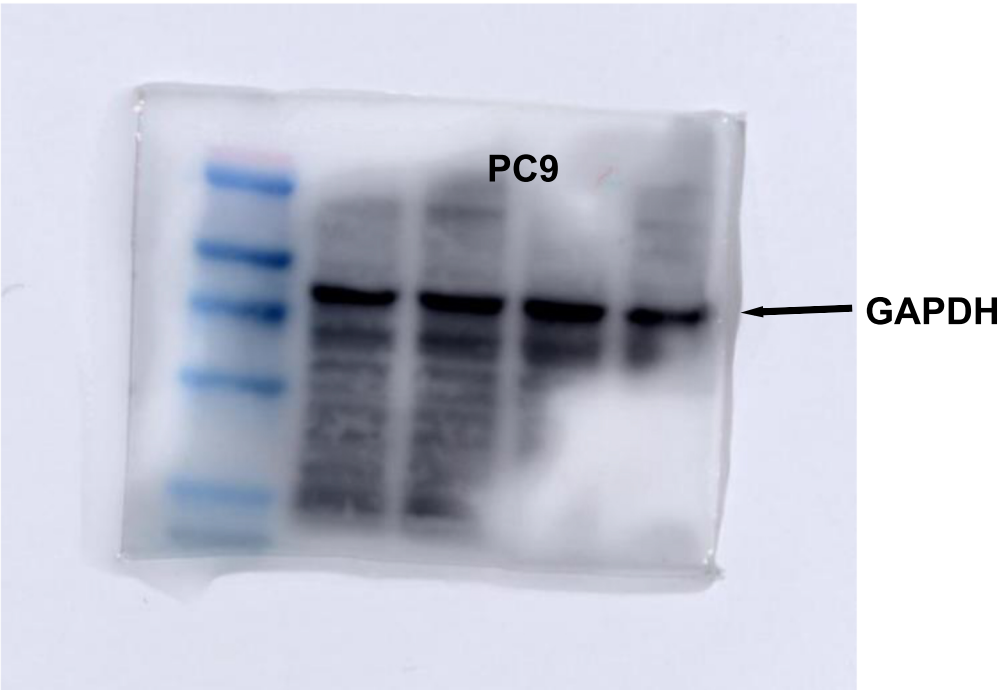

Figure S5E

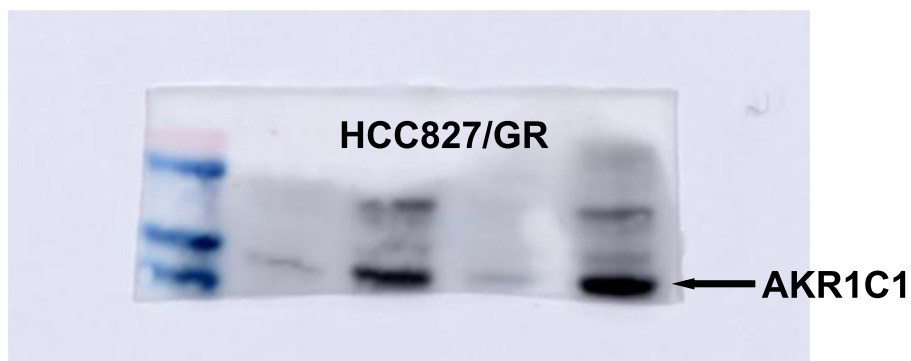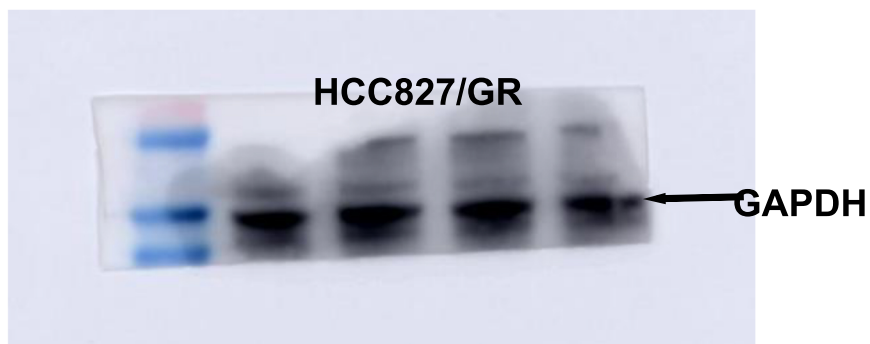

Figure S5F

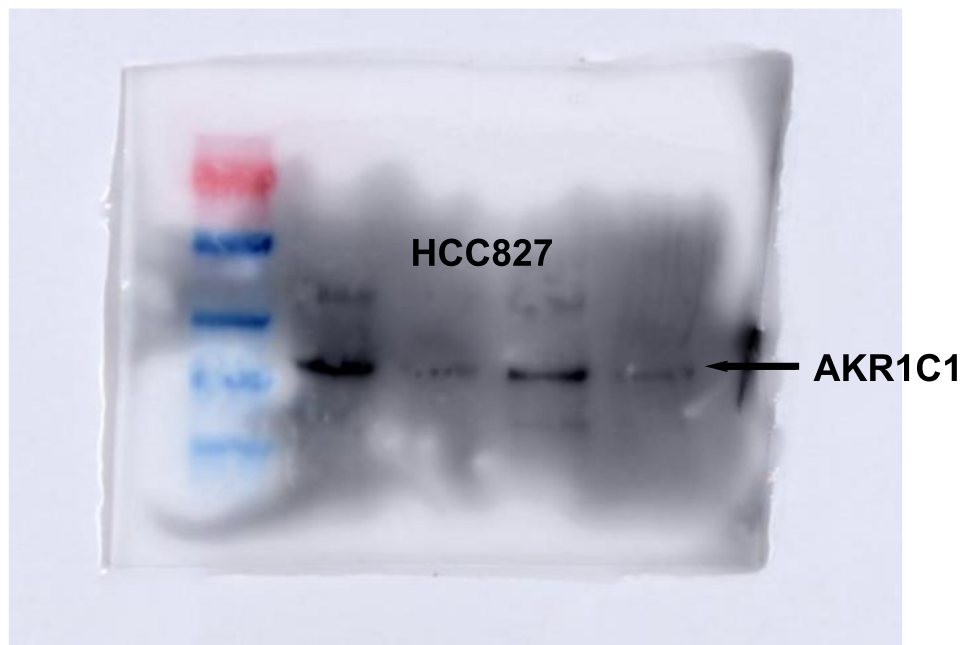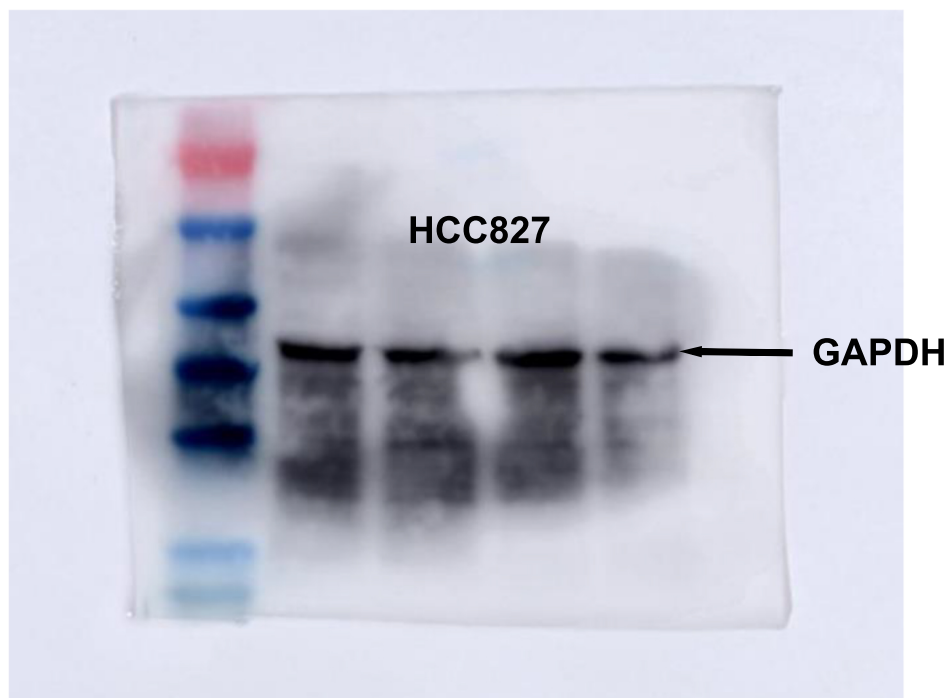

Supplement: Supplementary file 2 — uncropped original western blots [file 41420_2024_1892_MOESM2_ESM.pdf]
